# First-in-Class Dual EZH2-HSP90 Inhibitor Eliciting Striking Antiglioblastoma Activity *In Vitro* and *In Vivo*

**DOI:** 10.1021/acs.jmedchem.3c02053

**Published:** 2024-01-29

**Authors:** Sachin Sharma, Shao-An Wang, Wen-Bin Yang, Hong-Yi Lin, Mei-Jung Lai, Hsien-Chung Chen, Tzu-Yuan Kao, Feng-Lin Hsu, Kunal Nepali, Tsung-I Hsu, Jing-Ping Liou

**Affiliations:** † School of Pharmacy, College of Pharmacy, Taipei Medical University, Taipei 110, Taiwan; ‡ School of Respiratory Therapy, College of Medicine, Taipei Medical University, Taipei 110, Taiwan; § TMU Research Center of Neuroscience, Taipei Medical University, Taipei 110, Taiwan; ∥ Graduate Institute of Medical Sciences, College of Medicine, Taipei Medical University, Taipei 110, Taiwan; ⊥ TMU Research Center for Drug Discovery, Taipei Medical University, Taipei 110, Taiwan; # Department of Neurosurgery, Shuang Ho Hospital, Taipei Medical University, Taipei 110, Taiwan; ∇ Ph.D. Program in Medical Neuroscience, College of Medical Science and Technology, Taipei Medical University and National Health Research Institutes, Taipei 110, Taiwan; ○ International Master Program in Medical Neuroscience, College of Medical Science and Technology, Taipei Medical University, Taipei 110, Taiwan; ◆ TMU Research Center of Cancer Translational Medicine, Taipei 110 Taiwan; ¶ Ph.D. Program in Drug Discovery and Development Industry, College of Pharmacy, Taipei Medical University, Taipei 110, Taiwan

## Abstract

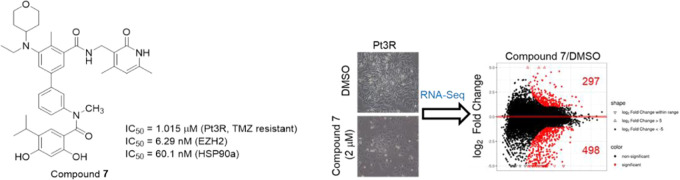

Structural analysis of tazemetostat, an FDA-approved EZH2 inhibitor, led us to pinpoint a suitable site for appendage with a pharmacophoric fragment of second-generation HSP90 inhibitors. Resultantly, a magnificent dual EZH2/HSP90 inhibitor was pinpointed that exerted striking cell growth inhibitory efficacy against TMZ-resistant Glioblastoma (GBM) cell lines. Exhaustive explorations of chemical probe **7** led to several revelations such as (i) compound **7** increased apoptosis/necrosis-related gene expression, whereas decreased M phase/kinetochore/spindle-related gene expression as well as CENPs protein expression in Pt3R cells; (ii) dual inhibitor **7** induced cell cycle arrest at the M phase; (iii) compound **7** suppressed reactive oxygen species (ROS) catabolism pathway, causing the death of TMZ-resistant GBM cells; and (iv) compound **7** elicited substantial *in vivo* anti-GBM efficacy in experimental mice xenografted with TMZ-resistant Pt3R cells. Collectively, the study results confirm the potential of dual EZH2-HSP90 inhibitor **7** as a tractable anti-GBM agent.

## Introduction

Glioblastoma (GBM), also known as grade IV astrocytoma, is characterized by a genetically unstable and highly infiltrative population of cells with a high degree of invasion.^1−5^ GBM tumor primarily originates from abnormal astrocytic cells,^6^ predominantly found in the frontal lobe, and can also metastasize to other parts of the brain via the ventricular system or corpus callosum, with sporadic incidents of spreading to the spinal cord.^7,8^ Surgical debulking flanked by radiotherapy and chemotherapy represents the currently recommended multimodality approach for the treatment of GBM; however, this trimodal therapy has been unable to attain the desired survival benefits.^9−12^ Lack of targeted agents, methylguanine DNA methyltransferase-mediated innate resistance to Temozolomide (oral alkylating agent, only first-line agent for GBM), cytological heterogeneity of GBM, and the existence of a highly tumorigenic population of cells (glioma stem cells, GSCs) are the main factors that hurdle the clinical success of the trimodal therapy.^13,14^ Given the unmet clinical needs for GBM treatment, new anti-GBM scaffolds that can target the GBM cancer cells and GSCs are highly desired.

**Figure 1:**
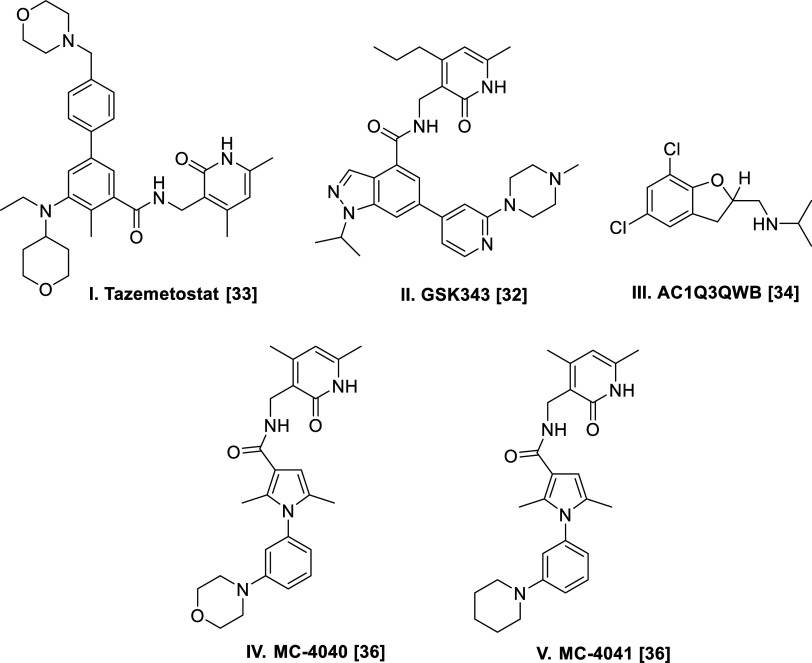
EZH2 Inhibitors with anti-GBM activity.

Our laboratory has been striving hard to enrich the chemical toolbox of anti-GBM scaffolds via the pragmatic design of epigenetic inhibitors.^15−19^ In continuation of our ongoing attempts, we planned to expand the program via the design of new structural assemblages that can address emerging GBM targets. An extensive literature survey led us to arrive at the emerging candidature of EZH2 as an epigenetic target for GBM. EZH2, a crux subunit of the Polycomb Repressive Complex (PRC2), is responsible for methylating lysine 27 (mono-, di-, and trimethylation) in histone H3 (H3K27) and H3K27me3 is more frequently interlinked with transcriptional repression.^20−22^ Notably, EZH2 is overexpressed in cancer stem cells of malignant tumors and plays a critical role in cancer stem cell expansion and maintenance.^23−28^ Several key revelations ascertain the involvement of EZH2 in GBM initiation and progression, such as (i) involvement of miR-206/Twist axis in EZH2-regulated malignancy of GBM cells,^29^ (ii) pro-oncogenic actions of Estradiol (proliferation, migration, and invasion) by EZH2 in GBM cells,^30^ and (iii) direct transcriptional regulation of *c-myc* by EZH2 leading to the maintenance of GSCs.^31^ To validate EZH2 as a therapeutic target in GBM and capitalize on the aforementioned revelations, several EZH2 inhibitors (Figure 1) were evaluated (monotherapy/combination therapy)^32−37^ and optimistic results were attained, for instance, (i) GSK343, a highly potent and selective EZH2 inhibitor, demonstrated the ability to counteract GBM progression via modulation of canonical/noncanonical *NF*-κB/IκBα pathways and immune response;^32^ (ii) a cocktail of tazemetostat (EZH2 inhibitor) and PI-103 (PI3K inhibitor) exerted significant reduction in GBM progression (U-87 cells);^33^ (iii) HOTAIR-EZH2 inhibitor AC1Q3QWB in combination with GSK-LSD1 inhibitor elicited enhanced antitumor efficacy in GBM patient-derived xenograft (PDX) models;^34^ (iv) combination of HOTAIR-EZH2 inhibitor (AQB) and palbociclib inhibitors suppressed the cell cycle in gliomas with high expression of EZH2 and low expression of CWF19L1;^35^ and (v) treatment with EZH2 inhibitors MC4040 and MC4041 led to impairment of primary GBM cell viability and weakened the aggressive malignant phenotype by reducing angiogenesis.^36^

Lately, researchers have tried to expand the activity spectrum of EZH2 inhibitors by appending their structural templates with other antitumor pharmacophores. Resultantly, dual-targeting EZH2 inhibitors, viz., solid tumor targeting dual EZH2/BRD4 inhibitors,^37^ dual PARP/EZH2 inhibitors for triple-negative breast cancer with wild-type BRCA,^38^ and dual G9A/EZH2 inhibitors for amplifying the antitumor immune response in ovarian high-grade serous carcinoma,^39^ were furnished. Much to the delight, a bifunctional EZH2 inhibitor (EZH2-HDAC dual inhibitor) demonstrating anti-GBM efficacy was also reported that hampered epithelial-to-mesenchymal transition by increasing the E-cadherin expression in GBM U87 cells.^40^

**Figure 2:**
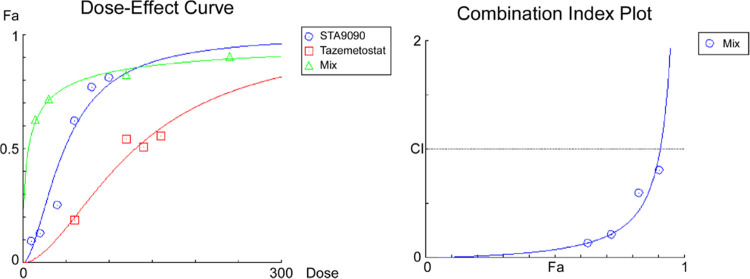
Pt#3R cells were treated with STA9090 and Tazemetostat to study the synergistic effect and dose–effect curve (DEC) and the combination index (CI) were analyzed by CompuSyn software. The effects (FA-fraction affected) of the indicated treatment combination were calculated using cell viability. Then, indicated concentrations and effects (FA-fraction affected) were input to the CompuSyn software and combination index (CI) values at different FA levels were analyzed automatically based on the Chou–Talalay method. The treatment combination effect was determined by the combination index (CI) as follows: combination index value <1, synergistic effect; = 1, additive effect; >1, antagonistic effect.

The preliminary/preclinical success of the aforementioned multitargeting agents strengthened our concievement to extract anti-GBM potential through logical stitching of the EZH2 inhibitory template with mechanistically diverse antitumor pharmacophores. Albeit the idea was clear, the design of dual inhibitors is a daunting task as selecting the second target needs a solid foundation. This made us turn to a campaign running in our laboratory that evaluates the anticancer efficacy of cocktails of epigenetic inhibitors and mechanistically diverse agents. Delightfully, synergistic effects were observed with the combination of Tazemetostat (EZH2 inhibitor) and STA9090 (HSP90 inhibitor) (Figure 2) and this served as the point of inception for this endeavor. As such, Hsp90 is an ATP-dependent molecular chaperone that regulates protein conformation, stability, and degradation.^41,42^ Several HSP90 inhibitors, viz., AUY922,^43^ YZ129,^44^ NW457,^45^ and BIIB021,^46^ have demonstrated anti-GBM efficacy at the preliminary level or the preclinical level. The pieces of evidence (our preliminary investigation and literature precedents) propelled us to design dual EZH2-HSP90 inhibitory chemical probes as anti-GBM agents in the present study.

## Rational Design

**Figure 3:**
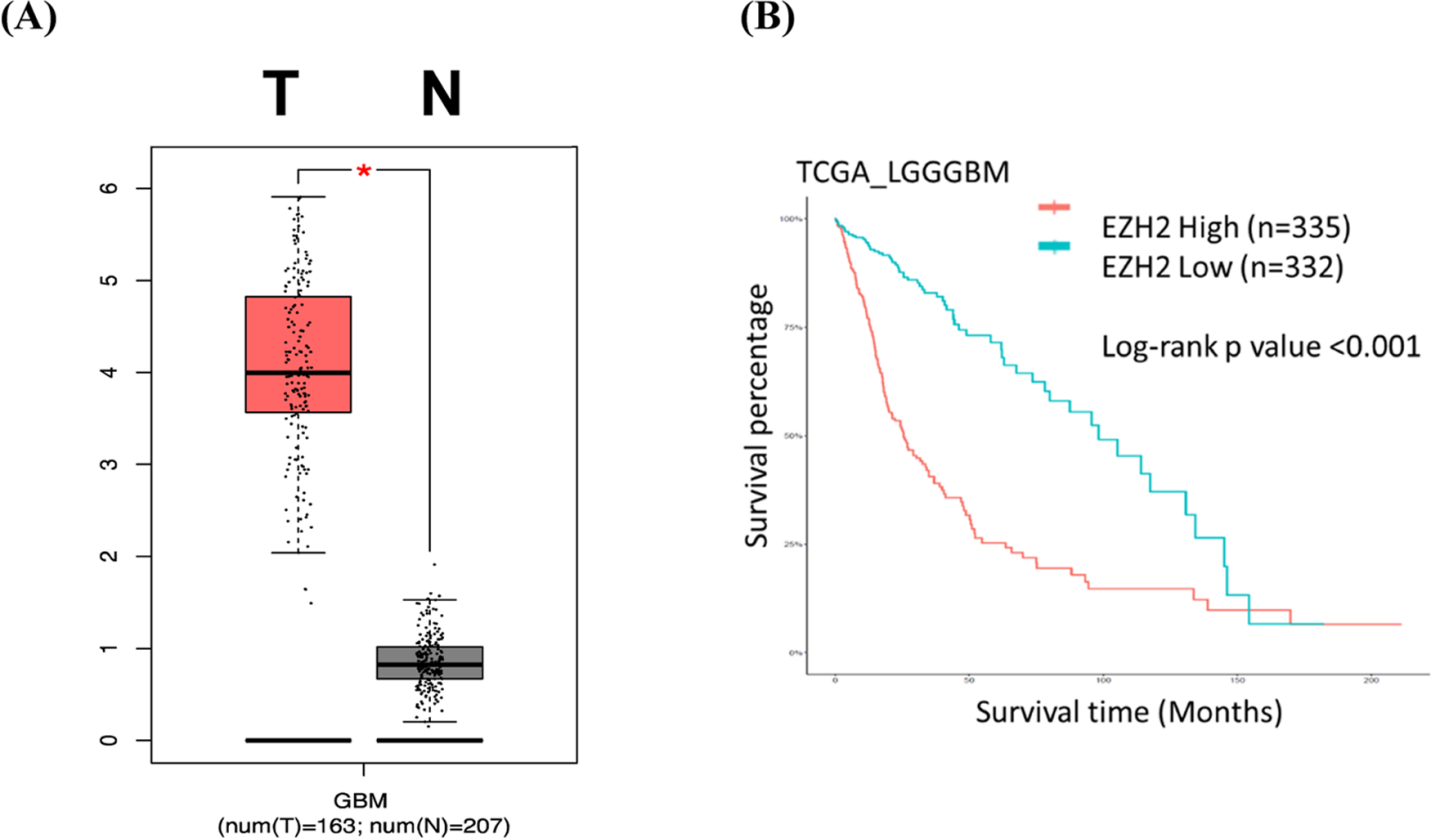
(A) Expression of EZH2 in normal brain tissues (N) and GBM tissues (T), and (B) survival analysis of EZH2 were performed using the Gepia and GlioVis website.

Before the commencement of the task (dual inhibitor hybrid scaffold design), we conducted a preliminary assessment of EZH2 expression in GBM tissues. As shown in Figure 3A, EZH2 was significantly increased in GBM tissues compared to normal brain tissues. Additionally, high expression of EZH2 was significantly correlated with a shorter survival time (Figure 3B), suggesting that EZH2 is an important oncogene in GBM pathogenesis. However, the prognostic value of HSP90AA1 with respect to the survival period in TCGA_LGGGBM data set lacks statistical significance (data not shown).

Then, the pursuit of capacitating the chemical architecture of tazemetostat with anti-GBM activity via the design of Tazemetostat-based bifunctional EZH2 inhibitory scaffolds was initiated. Structural analysis of Tazemetostat^47^ revealed that benzyl morpholine ring was a suitable site to accommodate the HSP90 inhibitory pharmacophore. In the context of the structural attributes of HSP90 inhibitors, resorcinol fragment was identified as a crucial structural unit that serves as a key binder associated with the ATP binding site of HSP90 proteins through an appropriate fit in the hydrophilic and hydrophobic regions of the protein.^48^

**Figure 4:**
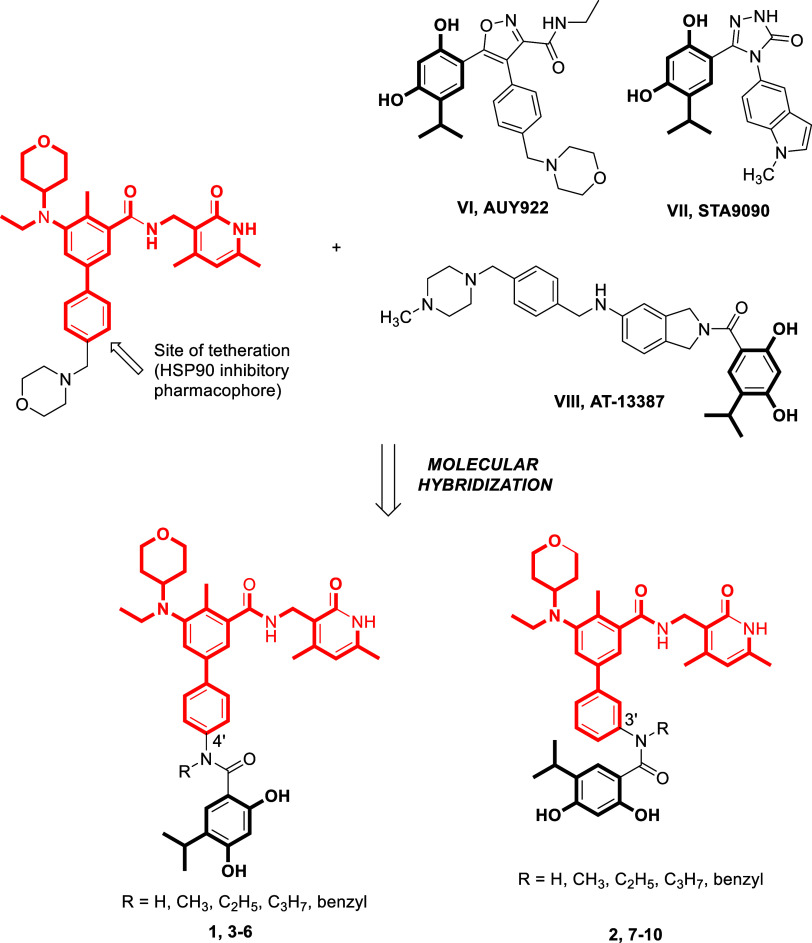
Design strategy.

With this background, a series of EZH2-HSP90 dual inhibitors (Tazemetostat-resorcinol hybrids) was designed (Figure 4) and synthesized via multistep synthetic routes. An exhaustive exploration of the designed adducts culminated in the identification of a strikingly potent hybrid structure that exerted magnificent cell growth inhibitory effects against TMZ-resistant GBM cells via balanced inhibition of EZH2 and HSP90. Also, the dual inhibitor suppressed kinetochore- and DNA repair-related gene expression and increased reactive oxygen species (ROS) accumulation through redox homeostasis disruption in mitochondria. Delightfully, the impressive *in vitro* anti-GBM profile of the dual inhibitor was also translated to the *in vivo* studies.

## Results

### Chemistry

**Scheme 1:**
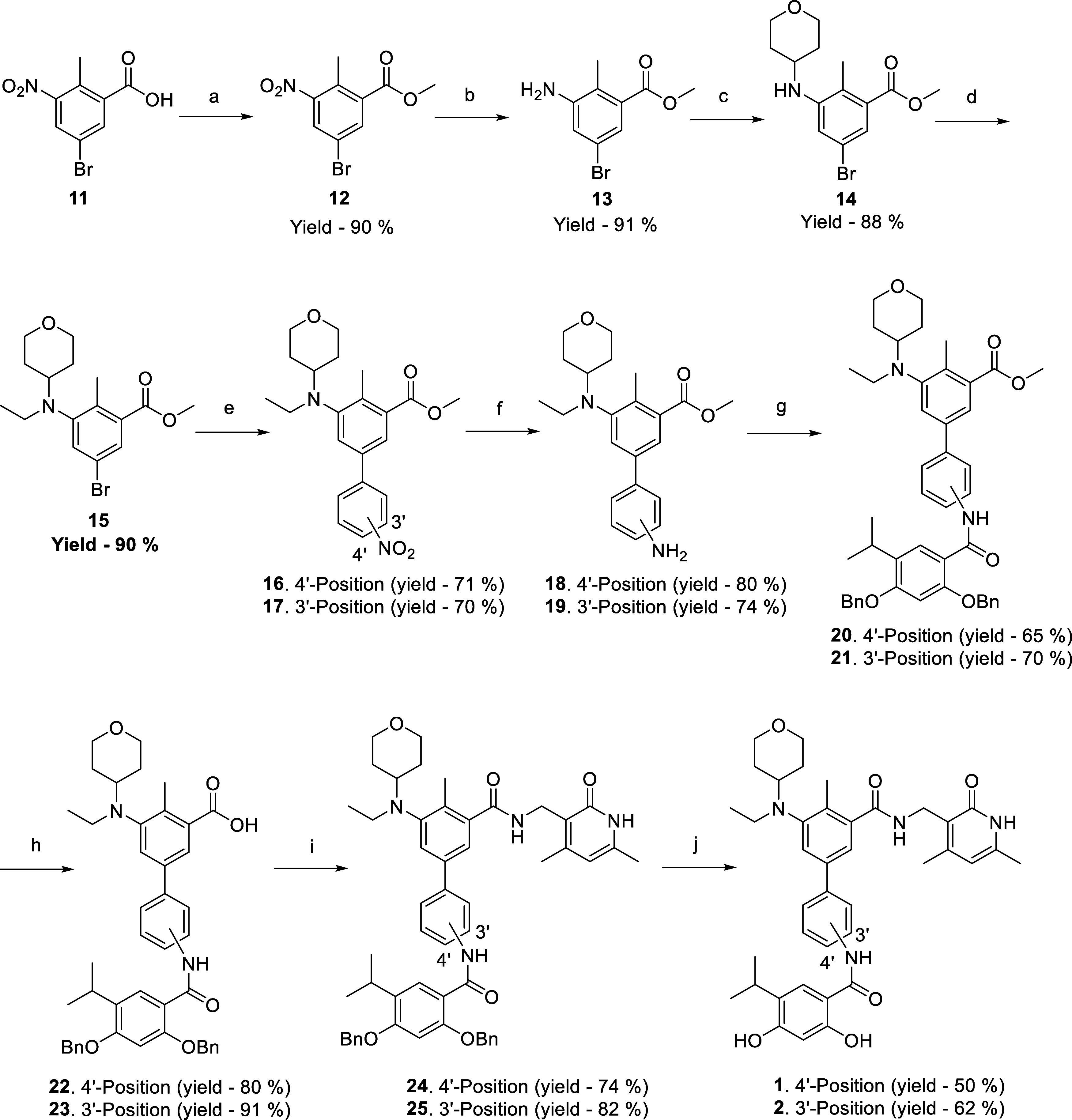
Reagents and Conditions: (a) CH_3_I, K_2_CO_3_, *N*,*N*-Dimethylformamide (DMF), rt, Overnight; (b) Fe, NH_4_Cl, EtOH/H_2_O (9:1), 100 °C, 2 h; (c) Tetrahydro-4*H*-pyran-4-one, CH_3_COOH, NaBH_3_CN, MeOH, Reflux, Overnight; (d) Acetaldehyde, CH_3_COOH, Na(CH_3_COO)_3_BH, 1,2-Dichloroethane (DCE), rt, 5 h; (e) 4-Nitrophenyl Boronic Acid or 3-Nitrophenyl Boronic Acid, Pd(PPh_3_)_3_, Na_2_CO_3_, Dioxane/Water (9:1), 60 °C, 2 h; (f) Fe, NH_4_Cl, EtOH/H_2_O (9:1), 100 °C, 2 h, (g) 2,4-Bis(benzyloxy)-5-isopropylbenzoic Acid, EDC·HCl, HOBT, *N*,*N*-Diisopropylethylamine (DIPEA), DMF, rt, 3 h; (h) LiOH(aq), Dioxane, rt, 3 h; (i) 3-(Aminomethyl)-4,6-dimethylpyridin-2(1*H*)-one, EDC·HCl, HOBT, DIPEA, DMF, rt, 3 h; (j) Pd/C, H_2_, MeOH, rt, 4 h

Central to our strategy of accomplishing a dual EZH2-HSP90 inhibitor was optimizing an efficient and robust multistep synthetic route. Thus, attempts were made to develop a high-yielding synthetic route for generating the designed compounds. For the accomplishment of unsubstituted amide-bearing hybrid scaffolds **1** and **2**, a reaction sequence outlined in Scheme 1 was leveraged. The route commenced with the high-yielding esterification of 5-bromo-2-methyl-3-nitrobenzoic acid (**11**) to generate the ester 1**2** (yield—90%). The nitro group bearing aromatic ester was then subjected to Fe/NH_4_Cl mediated reduction to afford the primary amine (**13**, yield—91%) which was reductively aminated consecutively, first to produce the pyran bearing intermediate (**14**, yield—88%) and then to furnish *N*-ethyl substitution bearing tertiary amine (**15**, yield—90%). Organopalladium catalyzed Suzuki arylation of intermediate **15** with (4-nitrophenyl)boronic acid and (3-nitrophenyl)boronic acid generated the biphenyl framework (**16**, yield—71% and **17**, yield—70%). Fe/NH_4_Cl-mediated nitro reduction transformed the nitro group containing biphenyls **16** and **17** to amine functionality bearing biphenyls **18** (yield—80%) and **19** (yield—74%). The biphenyls **18** and **19** were further amidated with 2,4-dihydroxy-5-isopropylbenzoic acid employing the carbodiimide-mediated methodology to accomplish the intermediates **20** (yield—65%) and **21** (yield—70%). Then, the ester functionality located on the trisubstituted phenyl ring of the biphenyl **20** and **21** was hydrolyzed using lithium hydroxide to produce the carboxylic acids **22** (yield—80%) and **23** (yield—91%). The acids were subsequently subjected to EDC/HOBt assisted amidation employing 3-(aminomethyl)-4,6-dimethylpyridin-2(1*H*)-one as the amine to obtain the intermediates **24** (yield—74%) and **25** (yield—82%). The intermediates **24** and **25** were then debenzylated using 10% Pd/C in methanol in a hydrogenation vessel with H_2_ at 40–42 psi to attain the hybrid scaffold **1** (yield—50%) and **2** (yield—62%).

**Scheme 2:**
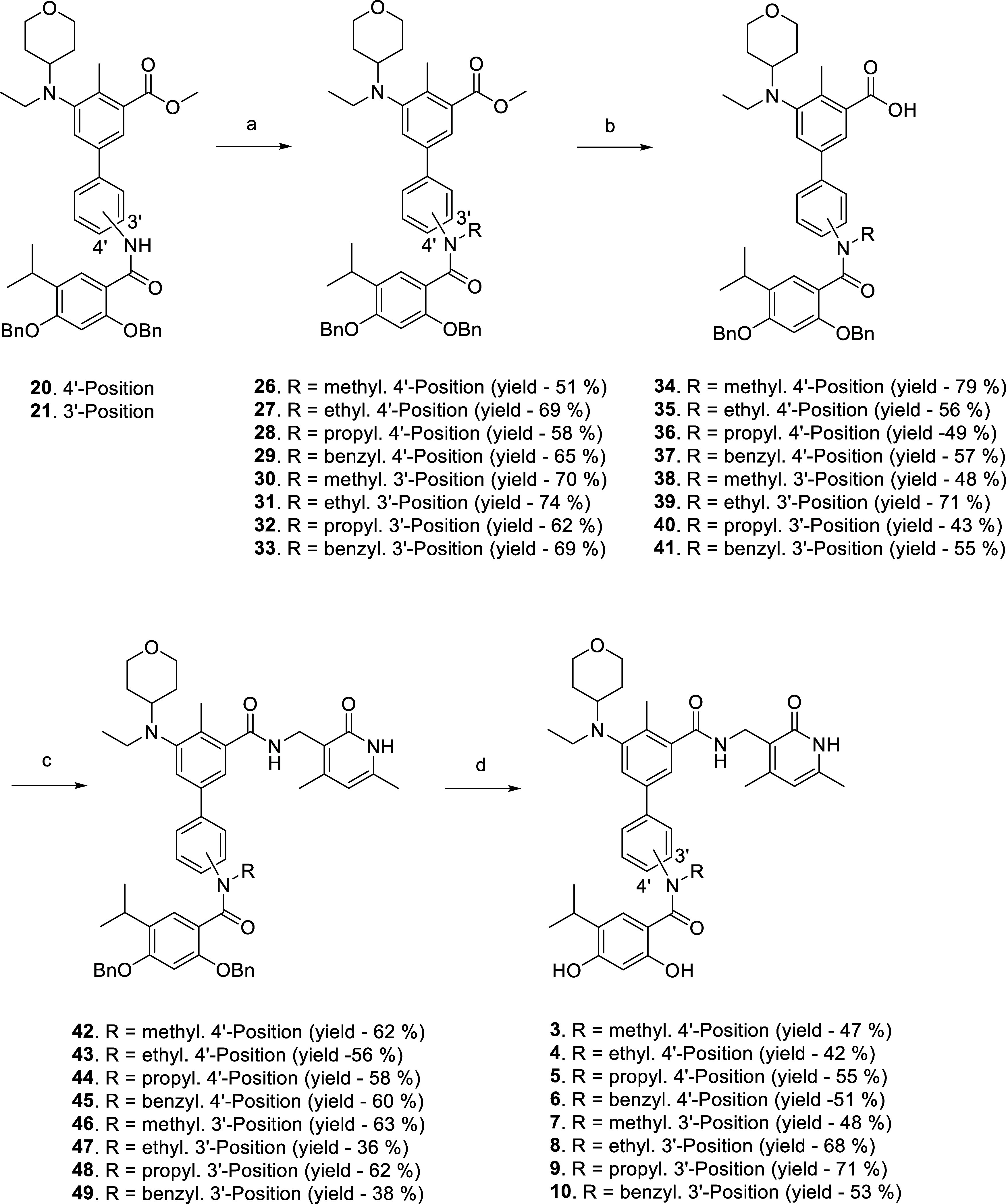
Reagents and Conditions: (a) Alkyl Iodide and Benzyl Bromide, NaH, DMF, rt, 4 h; (b) LiOH(aq), Dioxane, rt, 3 h; (c) 3-(Aminomethyl)-4,6-dimethylpyridin-2(1*H*)-one, EDC·HCl, HOBT, DIPEA, DMF, rt, 3 h; (d) for **42**–**44** and **46**–**48**, Pd/C, H_2_, MeOH, rt, 4 h; for **45** and **49**, BCl_3_, Dichloromethane (DCM), rt, 3 h

The synthetic route to the target scaffolds **3**–**10** is depicted in Scheme 2. The adducts **20** and **21** were utilized as versatile starting materials to obtain the *N*-substituted hybrid templates **3**–**10**. *N*-alkylation/benzylation of the intermediates **20** and **21** with alkyl iodides and benzyl bromides was afforded by exploiting the catalytic efficiency of NaH as a base at room temperature. The resulting intermediates **26**–**33** (yield—51–74%) were subjected to lithium hydroxide-assisted ester hydrolysis to get the carboxylic acids **34**–**41** (yield—43–79%) that were subsequently amidated with 3-(aminomethyl)-4,6-dimethylpyridin-2(1*H*)-one to generate intermediates **42**–**49** (yield—36–63%). The amides **42**–**44** and **46**–**48** were debenzylated using 10% Pd/C to accomplish the target hybrids **3**–**5** (yield—42–55%) and **7**–**9** (yield—48–71%). Noteworthy to mention that debenzylation of intermediates **45** and **49** was also attempted through palladium-mediated hydrogenation protocol; however, the methodology proved too capricious. It was intriguing to observe that even at room temperature a competition between *N*-debenzylation and *O*-debenzylation was observed (not shown in the scheme). Thus, a different tactic to selectively debenzylate the *O*-benzyl groups was optimized and BCl_3_ was used to attain such selectivity and furnish **6** (yield—51%) and **10** (yield—53%). Delightfully, both the palladium-based and the BCl_3_-based methodologies led to satisfactory yields of the target compounds.

### EZH2 Inhibition

After the successful construction of the target adducts (**1**–**10**), they were profiled for EZH2 inhibitory potential. The results of the study are depicted in Table 1. Careful observation of the results gave us some insights regarding the structure—EZH2 inhibition relationship. Five adducts (**1–4, 7**) elicited outstanding EZH2 inhibitory potential with IC_50_ values in the single-digit nanomolar range. The key notion generated from the correlation of the structural features of the adducts with EZH2 inhibitory activity revealed that tetheration of the resorcinol fragment via amide bond at 3′ position was favored over the tetheration at 4′ position as compound **2** (resorcinol fragment, 3′ position) manifested a striking EZH2 inhibitory activity with an IC_50_ value of 2.06 nM. Notably, compound **2** was 3-fold more potent in inhibiting EZH2 than its counterpart **1** (resorcinol fragment, 4′ position). To test the tolerance of *N*-alkylation/benzylation (amide NH), the generated adducts **3**–**10** were also evaluated. Notably, the impact of *N*-alkylation/benzylation was quite apparent to be detrimental for the EZH2 inhibitory potential and the trend of relatively reduced enzyme inhibition activity was evidenced in regio-counterparts (resorcinol fragment at 4′ as well as 3′ position). A dig into the enzyme inhibitory profile of compound **3**–**10** perspicuously ascertains the aforementioned trend as compound **1** outshines its *N*-alkylated/benzylated derivatives (**3**–**6**), Likewise, compound **2** was observed to be stupendously more effective in inhibiting the enzymatic catalytic subunit of polycomb repressive complex 2 than its *N*-alkylated/benzylated derivatives (**7**–**10**). The other key structural notions generated from the assay were the highest degree of decrease in EZH2 inhibitory activity observed with *N*-benzylation (*N*, amide bearing the resorcinol fragments) in both cases (amide-tethered at position 4′ and amide tethered hybrids at position 3′) and the higher magnitude of downward trends witnessed with *N*-substitution in the hybrid scaffolds flanking the resorcinol fragment at position 3′. Albeit, some potent EZH2 inhibitors were pinpointed through this evaluation study, a relative comparison with the EZH2 inhibitory activity of tazemetostat (**I**) indicated that even the best four out of the 10 hybrids furnished were endowed with a slightly inferior EZH2 inhibitory profile. Important to mention that this observation did not prove to be a hindrance in our endeavor as tazemetostat (**I**) despite being a magnificently potent EZH2 inhibitor could not exert cell growth inhibitory potential against the GBM cell lines as per our preliminary screening results, some literature precedents as well as the comparative analysis report of tazemetostat (**I**) vs hybrid scaffolds (**1**–**10**, Table 6). Thus, we anticipated that the balanced concomitant modulation of EZH2 and HSP90 via the furnishing of hybrid scaffolds based on the structural commonalities of pharmacophores of both targets might activate the chemical architecture of tazemetostat to exert anti-GBM effects. In light of this anticipation, our research group was inclined toward the pursuit of identifying a dual EZH2-HSP90 inhibitor and accordingly, proceeded toward the evaluation of the HSP90 inhibition activity of the adducts (**1**–**10**).

**Table 1:** EZH2 Inhibitory Activity of the Synthesized Adducts

compounds	EZH2 IC_50_ (nM)^a^
**1**	6.14
**2**	2.06
**3**	7.21
**4**	7.35
**5**	21.8
**6**	11.7
**7**	6.29
**8**	36.6
**9**	40.1
**10**	641
**Tazemetostat**	0.697

a Conducted by Reaction Biology Corp. (Pennsylvania), the protocol involved the testing of compounds (dissolved in dimethyl sulfoxide) in at least 10-dose IC_50_ mode. Specifically, the compounds (starting at 10 μM) were tested with 3-fold serial dilution, and IC_50_ values were calculated based on the results of a single experiment.

### HSP90 Inhibition

An *in vitro* experiment was performed to assess the inhibitory effect of the synthesized adducts on the heat shock protein 90 (HSP90). Geldanamycin was employed as a standard HSP90 inhibitor. It was a bit intriguing to observe that substitution at the amide NH (bearing the resorcinol fragment) had a galactic influence on the ability of the generated adducts to inhibit the chaperone protein. As such, a reversed pattern of increased HSP90 inhibitory activity was evidenced with *N*-alkylation/benzylation in this assay as compared to the results of the EZH2 inhibitory assay. Hybrid structures **1** and **2** were devoid of HSP90 inhibitory potential; however, methylation and ethylation at the amide nitrogen conferred HSP90 inhibitory potential to the scaffolds (**3**, **4**, **7**, and **8**). Disappointingly, *N*-propyl and -benzyl substitutions (**5**, **6, 9**, and **10**) could not replicate the activity conferring trends as that of the *N*-methyl and ethyl group and it was conceived that the placement of the bulkier substituent was not tolerable at the amide NH (bearing the resorcinol fragment). Notably, the hybrid template featuring the amide bond connector (for the resorcinol fragment) at position 3′ benefited the most in the context of activation of the chemical architectures toward the HSP90 inhibitory activity. *N*-methyl and ethyl substitution at the amide NH of target scaffolds encompassing the resorcinol fragment at position 3′ culminated into two substantially potent HSP90 inhibitors, compounds **7** (IC_50_ value = 60.1 nM) and **8** (IC_50_ value = 63.6 nM) (Table 2). As observed in the EZH2 inhibitory evaluation study, the adducts replicated the trend of not being more potent than the standard employed (geldanamycin, in HSP90 inhibitory assay and tazemetostat in EZH2 inhibitory assay). Unperturbed by the observed inferior profiles of the adducts in both the assays, pinpointing a balanced dual EZH2-HSP90 inhibitor **7** endowed with IC_50_ values of 6.29 nM (EZH2 inhibition) and 60.1 nM (HSP90 inhibition), respectively, was a matter of delight for our research group.

**Table 2:** HSP90 Inhibitory Activity of Adducts **1**–**10**

compound ID	HSP90a IC_50_ (nM)^a^
**1**	-
**2**	-
**3**	343
**4**	823
**5**	-
**6**	>10 000
**7**	60.1
**8**	63.6
**9**	9330
**10**	2840
**Geldanamycin**	20.9

a Conducted by Reaction Biology Corp. (Pennsylvania), the protocol involved the testing of compounds (dissolved in dimethyl sulfoxide) in at least 10-dose IC_50_ mode. Specifically, the compounds (starting at 10 μM) were tested with 3-fold serial dilution, and IC_50_ values were calculated based on the results of a single experiment.

### Itraq-Based Proteomics Analysis

Having identified the desired chemical probe **7**, we assessed global protein expression using Itraq-based proteomics analysis to estimate whether **7** suppresses HSP90 (Pt3R cell lines). As shown in Tables 3–5, HSP family proteins, including HSPA1A, HSPA8, HSP90AA1, HSPB1, HSPH1, and HSPA4, were significantly increased in response to dual inhibitor **7** treatment. This observation is aligned with the results attained with geldanamycin treatment (a well-known HSP90 inhibitor)^49,50^ and confirms the HSP90-suppressive activity of compound **7** in GBM cells.

**Table 3:** Compound 7-Influenced Proteins Grouped Functionally by KEGG Analysis

trend	term in KEGG_PATHWAY	genes	*P*-value
up-regulation	hsa04612:Antigen processing and presentation	HSPA8, HSP90AA1, HSPA4, HSPA1A	7.88 × 10^–04^
up-regulation	hsa04141:Protein processing in endoplasmic reticulum	HSPA8, HSP90AA1, HSPH1, HSPA1A	7.69 × 10^–03^
up-regulation	hsa04144:Endocytosis	ARF3, EHD1, HSPA8, HSPA1A	2.01 × 10^–02^
up-regulation	hsa04915:Estrogen signaling pathway	HSPA8, HSP90AA1, HSPA1A	2.42 × 10^–02^
up-regulation	hsa04390:Hippo signaling pathway	SERPINE1, CTNNB1, ACTB	5.25 × 10^–02^
up-regulation	hsa05164:Influenza A	HSPA8, ACTB, HSPA1A	6.75 × 10^–02^
up-regulation	hsa04510:Focal adhesion	ZYX, CTNNB1, ACTB	9.04 × 10^–02^
down-regulation	hsa03010:Ribosome	RPS6, RPL35, RPL26, RPS10	8.84 × 10^–03^
down-regulation	hsa04110:Cell cycle	MCM7, MCM3, YWHAG	5.89 × 10^–02^
down-regulation	hsa03040:Spliceosome	HNRNPA3, PCBP1, SRSF7	6.66 × 10^–02^

**Table 4:** Proteins Increased by Compound **7** (2 μM) Treatment Analyzed by iTRAC Assay

accession	gene name	score	C7/DMSO
P35222	CTNNB1	137.20	1.550
Q9H4M9	EHD1	59.69	1.555
P34932	HSPA4	312.76	1.561
O00487	PSMD14	57.97	1.579
O15460	P4HA2	105.19	1.588
P36543	ATP6 V1E1	41.77	1.599
Q92598	HSPH1	123.02	1.642
Q9UII2	ATP5IF1	45.22	1.646
P60709	ACTB	1504.25	1.657
P04792	HSPB1	264.25	1.661
P60903	S100A10	243.10	1.664
P30533	LRPAP1	80.20	1.667
O14908	GIPC1	27.10	1.699
P61604	HSPE1	133.46	1.710
Q9NTK5	OLA1	37.59	1.745
P24534	EEF1B2	75.42	1.792
O43242	PSMD3	36.87	1.801
Q15942	ZYX	24.18	1.903
P19367	HK1	88.39	1.921
P61204	ARF3	41.00	1.944
P07900	HSP90AA1	958.86	1.958
Q12792	TWF1	39.76	1.995
P11142	HSPA8	959.46	2.096
Q56VL3	OCIAD2	41.53	2.150
P50454	SERPINH1	113.69	2.186
O00299	CLIC1	74.34	2.598
P0DMV8	HSPA1A	469.78	2.843
P15531	NME1	188.40	3.299
P05121	SERPINE1	50.02	4.203

**Table 5:** Proteins Decreased by Compound **7** (2 μM) Treatment Analyzed by iTRAC Assay

accession	gene name	score	C7/DMSO
Q71DI3	HIST2H3A	93.71	0.240
Q96KK5	HIST1H2AH	157.12	0.245
P16403	H1–2	552.01	0.334
P51991	HNRNPA3	121.56	0.377
P08758	ANXA5	30.37	0.406
Q9ULV4	CORO1C	53.84	0.410
O60814	H2BC12	211.06	0.419
P06899	H2BC11	220.59	0.423
P21589	NT5E	71.63	0.446
P61981	YWHAG	112.04	0.447
O00148	DDX39A	156.38	0.515
P62805	H4C1	598.09	0.556
P68371	TUBB4B	763.06	0.564
O43169	CYB5B	36.86	0.568
O15347	HMGB3	22.25	0.573
P26583	HMGB2	128.00	0.575
Q68CZ2	TNS3	52.27	0.582
P33993	MCM7	50.38	0.586
Q15185	PTGES3	125.85	0.589
P55084	HADHB	35.40	0.591
P26196	DDX6	92.60	0.596
P0C0S5	H2AZ1	180.00	0.603
P49321	NASP	150.44	0.604
Q16629	SRSF7	31.45	0.605
P46783	RPS10	88.48	0.608
Q92804	TAF15	55.54	0.609
P61254	RPL26	82.88	0.619
Q13347	EIF3I	65.65	0.624
P63244	RACK1	121.61	0.627
P62879	GNB2	118.48	0.628
Q15365	PCBP1	80.97	0.628
Q9UQ80	PA2G4	235.25	0.628
P42766	RPL35	20.64	0.629
P35237	SERPINB6	27.22	0.632
Q8NBS9	TXNDC5	22.91	0.635
P25205	MCM3	44.10	0.635
Q99584	S100A13	44.16	0.638
P62753	RPS6	41.92	0.641
Q12906	ILF3	103.00	0.645

### Structure-Based Molecular Docking

**Figure 5:**
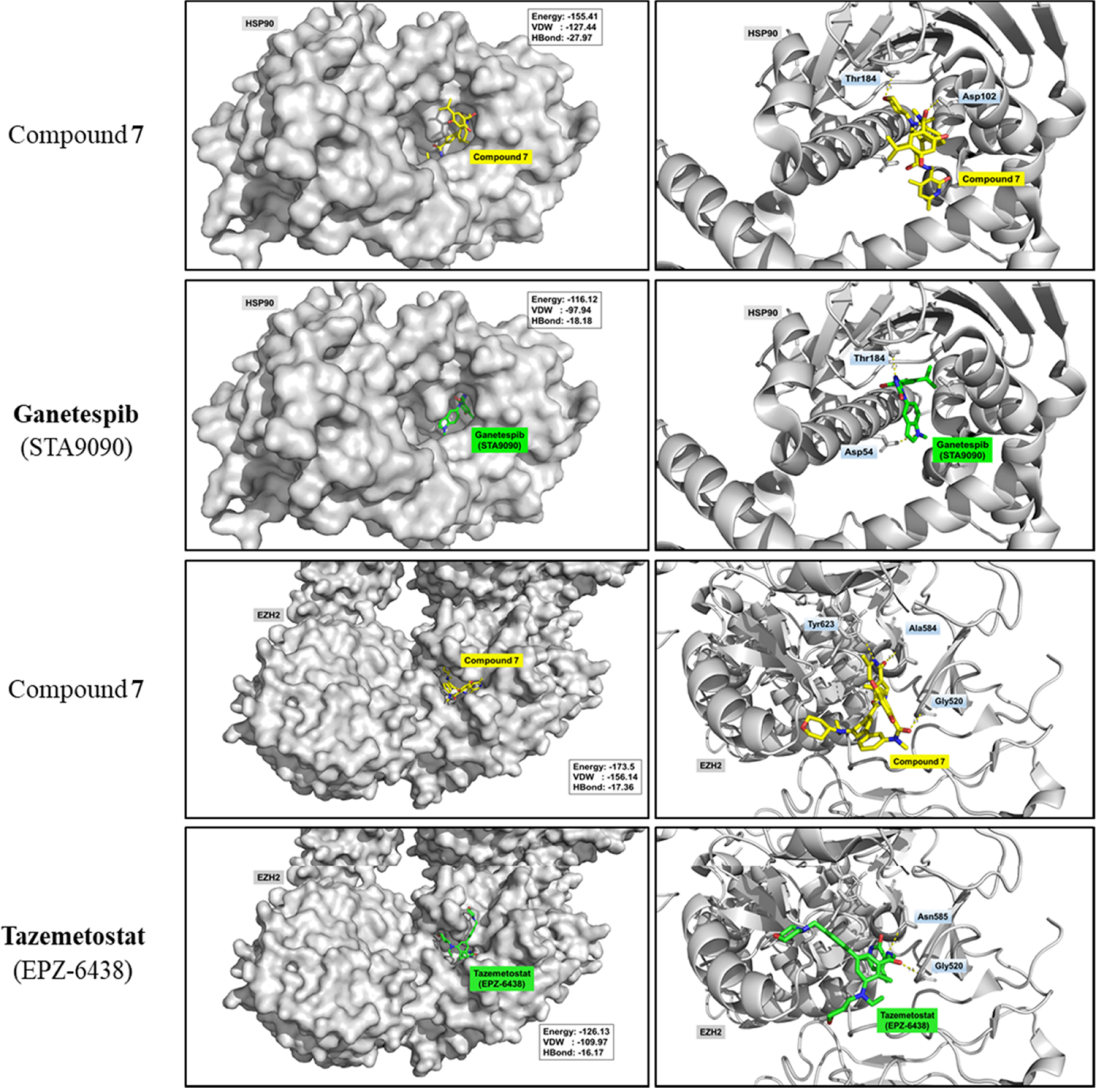
Structure-based molecular docking of compound **7**. Protein structures of HSP90 and EZH2 were obtained from Protein Data Bank (8AGI and 4W2R).

Structure-based molecular docking was performed to rationalize the experimental results of the enzymatic assays (Figure 5). The docking process was carried out between protein and compounds with a population size of 1500, generations of 150, and solutions of 10 through iGEMDOCK version 2.1. Protein structures of HSP90 and EZH2 were obtained from the Protein Data Bank (8AGI and 4W2R). Notably, compound **7** was found to be associated with the ATP binding pocket of HSP90 which is a target of a well-known HSP90 inhibitor, ganetespib (STA9090).^51^ In addition, similar to an EZH2 inhibitor, tazemetostat, compound **7** was observed to be associated with a site that is responsible for mediating the methyl transfer reaction of EZH2.^52^ These results indicated that compound **7** has a high specificity targeting ability to HSP90 and EZH2.

### *In Vitro* Cytotoxicity Studies

**Figure 6:**
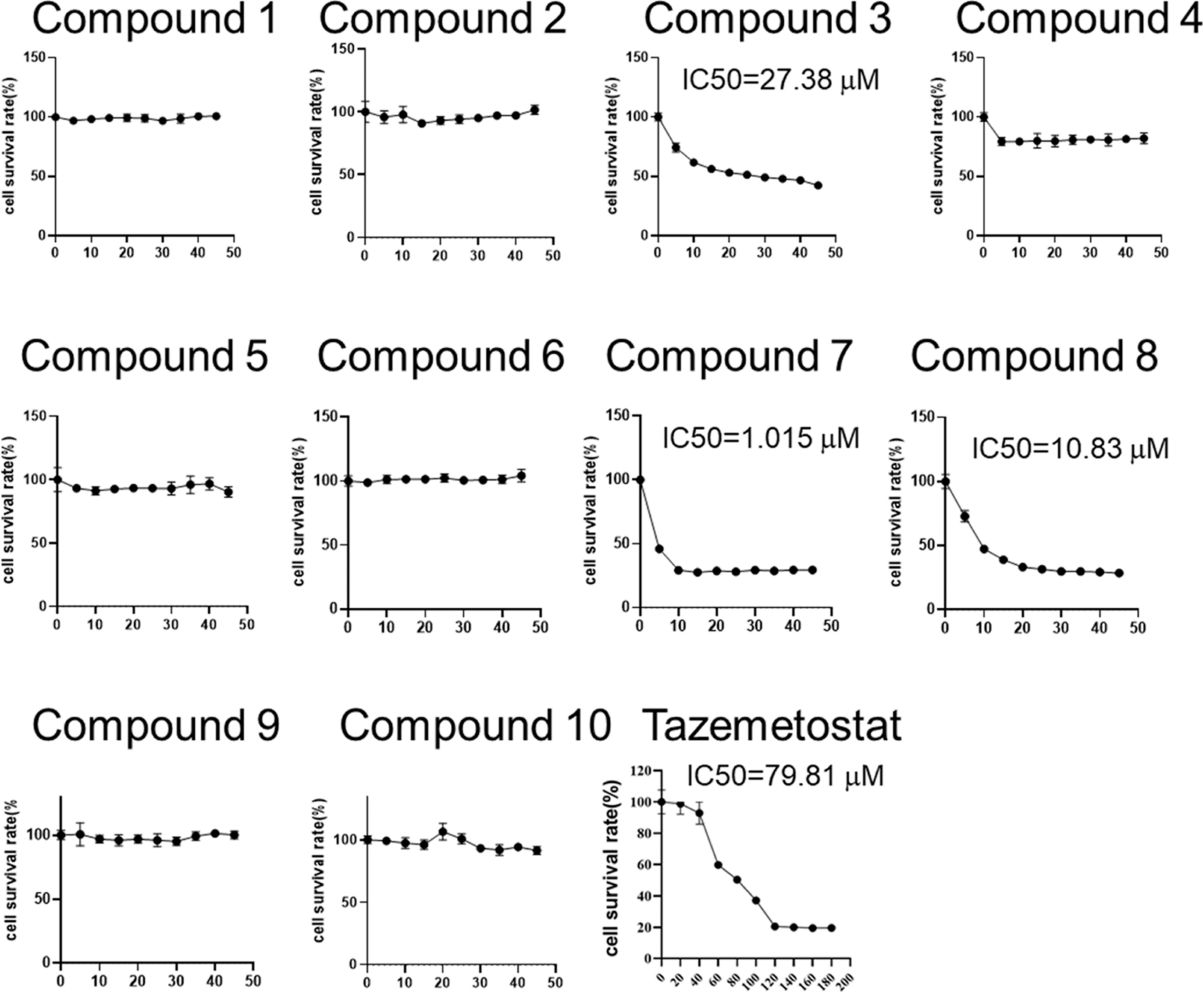
Effects of Epz-6438-derived inhibitors on the viability of Pt3R cells. After treatment for 72 h, cell viability was analyzed using CCK8 assay and IC_50_ was generated using the Prism software.

*In vitro* cytotoxicity studies were carried out to validate whether the hypothesis of extracting anti-GBM effects via balanced dual modulation of EZH2 and HSP90 corroborates with the experimental results (Table 6, Figure 6). Thus, the adducts (**1**–**10**) were evaluated for their ability to inhibit the cell viability of TMZ-resistant cells (Pt3-R). Delightfully, the most potent and balanced EZH2-HSP90 dual inhibitor **7** demonstrated significant cell proliferation inhibitory potential against Pt3-R cell lines with an IC_50_ value of 1.015 μM. A correlation of the results of Tables 1, 2, and 6 indicates that the adducts (**1, 2, 4–6, 9**, and **10**) as well as tazemetostat solely acting as EZH2 inhibitors were unable to exert cell growth inhibitory effects and modulation of both the targets (EZH2 and HSP90) was mandatory for the anti-GBM efficacy. Notably, compounds **3**, **7** and **8** which can be categorized as dual inhibitors were the only ones to exert cell viability inhibitory potential. Among them, compound **7** [the most balanced and potent dual inhibitor of the targets (EZH2-HSP90)] was the most effective against the Pt3R cell. Importantly, compound **7** is able to penetrate blood–brain barrier (BBB) to exhibit anti-GBM efficacy *in vitro* (Figure S1). Given the results, it is perspicuous that the magnificent enzyme inhibitory profile of compound **7** translated into cytotoxicity against the TMZ-resistant cells (Pt3R) GBM cell lines. In a nutshell, it is deduced that cell growth inhibitory effects of compound **7** stem from its ability to modulate EZH2 and HSP90 chaperone in a balanced manner.

**Table 6:** Cell Growth Inhibitory Effects at 72 h of Compounds **1**–**10** in TMZ-Resistant Cells (Pt3R)

compounds	IC_50_ (μM)
**1**	
**2**	
**3**	27.38 ± 1.58
**4**	
**5**	
**6**	
**7**	1.015 ± 0.69
**8**	10.83 ± 1
**9**	
**10**	
**Tazemetostat**	79.81 ± 4.91

### Dual Inhibitor **7** Suppressed Kinetochore- and DNA Repair-Related Gene Expression

**Figure 7:**
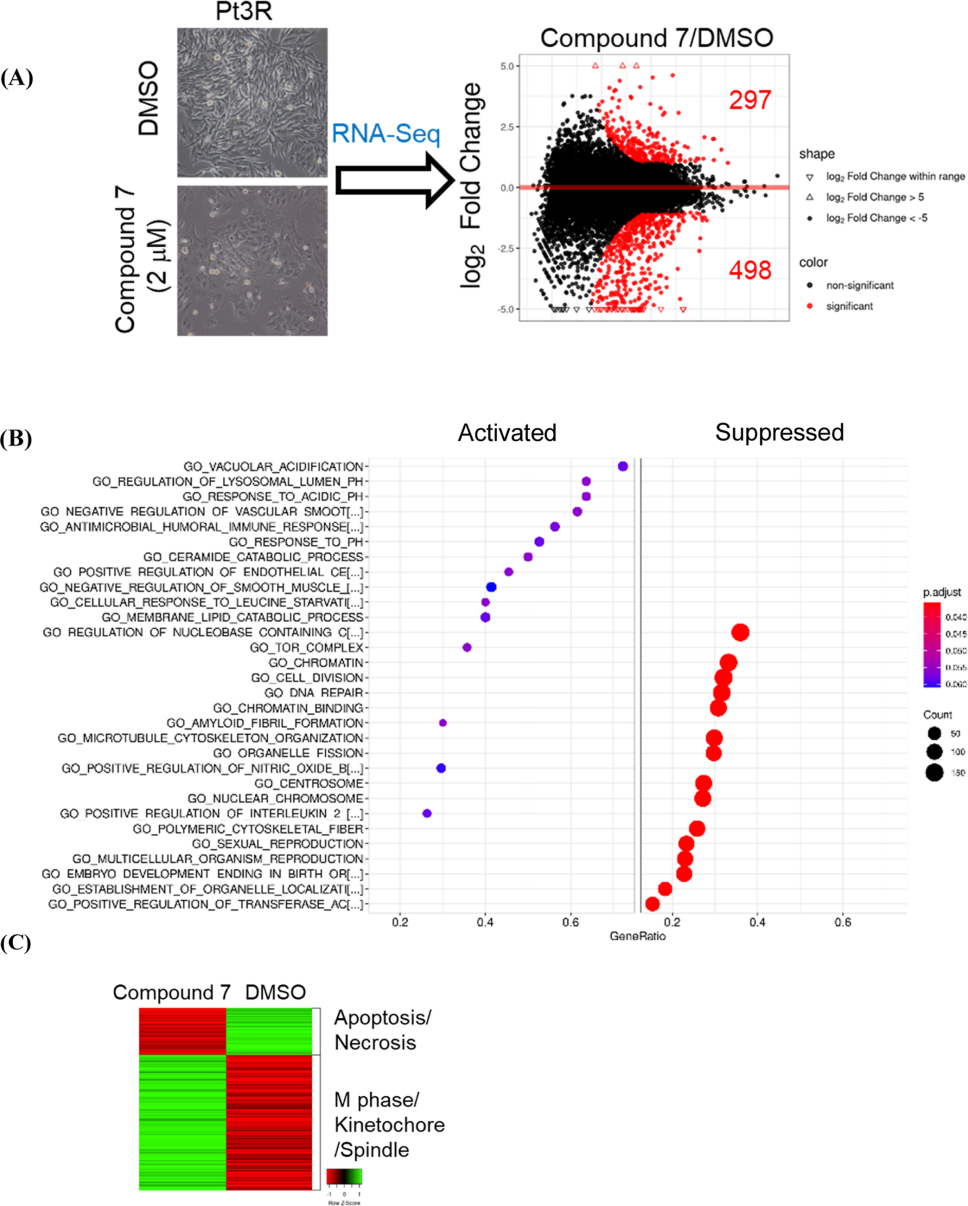
Effect of compound **7** on gene expression profile in TMZ-resistant Pt3R cells. (A) After treatment for 48 h, RNA extracts were collected and subjected to RNA-seq; (left) cell morphology, (right) genes significantly influenced by compound **7**. (B) GSEA functionally grouped genes. (C) Genes involved in apoptosis/necrosis and M phase/Kinetochore/Spindle were clustered in the heatmap.

To gain insights regarding the ability of dual inhibitor **7** to suppress the viability of TMZ-resistant GBM cells, RNA-seq to analyze gene expression profiles was performed. As shown in Figure 7A, treatment of Pt3R with 2 μM of **7** for 48 h significantly inhibited cell proliferation. Also, RNA-seq revealed that compound **7** increased and decreased 297 and 498 gene expressions, respectively. Furthermore, the outcome of Gene Set Enrichment Analysis (GSEA) of compound **7** indicated that cell division and DNA repair were highly suppressed (Figure 7B). Notably, treatment with **7** increased apoptosis/necrosis-related gene expression, whereas decreased M phase/kinetochore/spindle-related gene expression (Figure 7C).

### Compound **7** Decreased CENPs Protein Expression

**Figure 8:**
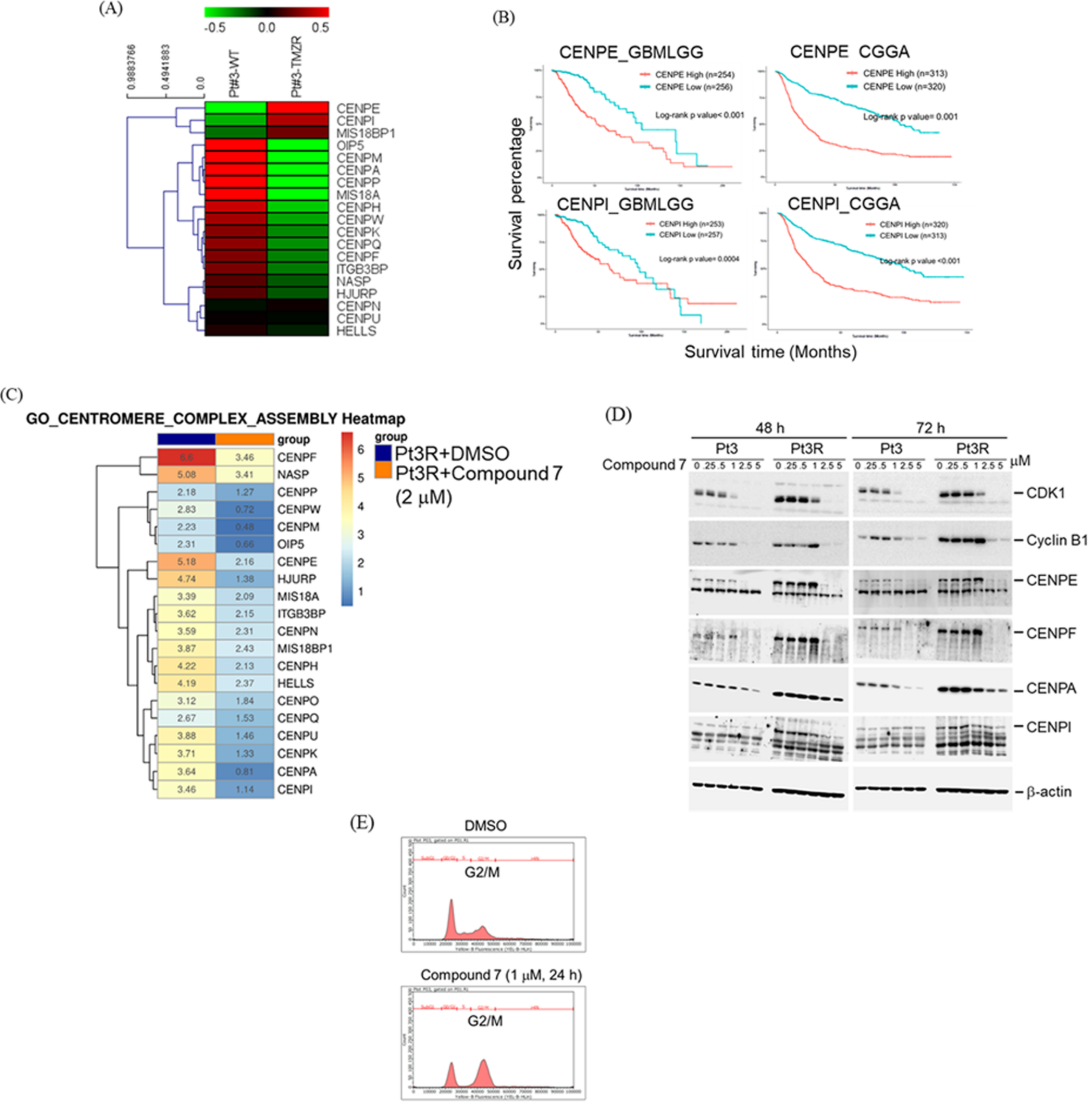
Compound **7** suppresses gene expression of CENP family. (A) Compound **7**-suppressed CENP family. (B) Gene expression of CENP family in Pt3 and TMZ-resistant Pt3R cells. (C) Prognostic value was analyzed using the GlioVis Web site. (D) Western blotting was used to confirm that compound **7** decreased CENPs expression. (E) Cell cycle analysis. After treatment for 24 h, cells were stained with propidium iodide (PI) and subjected to flow cytometry analysis.

Furthermore, the impact of compound **7** treatment on centromere proteins (CENPs) was assessed. As such, CENPs are the main constituent proteins of the kinetochore, which are essential for cell division. CENPs expression is elevated in GBM tissues and correlated with unfavorable overall survival in glioma patients.^53^ Before evaluating the effect of dual inhibitor **7**, a study to assess the expression levels of CENPs was carried out and the upregulated expression of CENPE and CENPI was evidenced in TMZ-resistant GBM cells (Pt3R) compared to patient-derived GBM cells, Pt3 (Figure 8A). It was also observed that CENPE and CENPI were significantly correlated with poor prognosis of GBM patients (Figure 8B), suggesting that CENPs are important for GBM progression. Delightfully, further investigation results revealed that centromere proteins (CENPs) including CENPF, CENPE, CENPA, and CENPI which are important to regulate kinetochore assembly and mitosis were decreased by compound **7** treatment (Figure 8C). Western blot analysis was also performed and the outcome indicated that hybrid scaffold **7** also downregulated the expression of CDK1 and cyclin B1, both of which are required to progress M phase (Figure 8D). Additionally, CENPs protein expression was also decreased by **7** in Pt3R cells (Figure 8D). Also, dual inhibitor **7** induced cell cycle arrest at the M phase (Figure 8E). We found that compound **7** is able to block cell cycle progression at 24 h treatment (Figure 8E), and this inhibitory effect on cell cycle was maintained, at least, for 72 h (Figure 8D) *in vitro*.

### Compound **7** Downregulated the DNA Repair-Related Gene Expression

**Figure 9:**
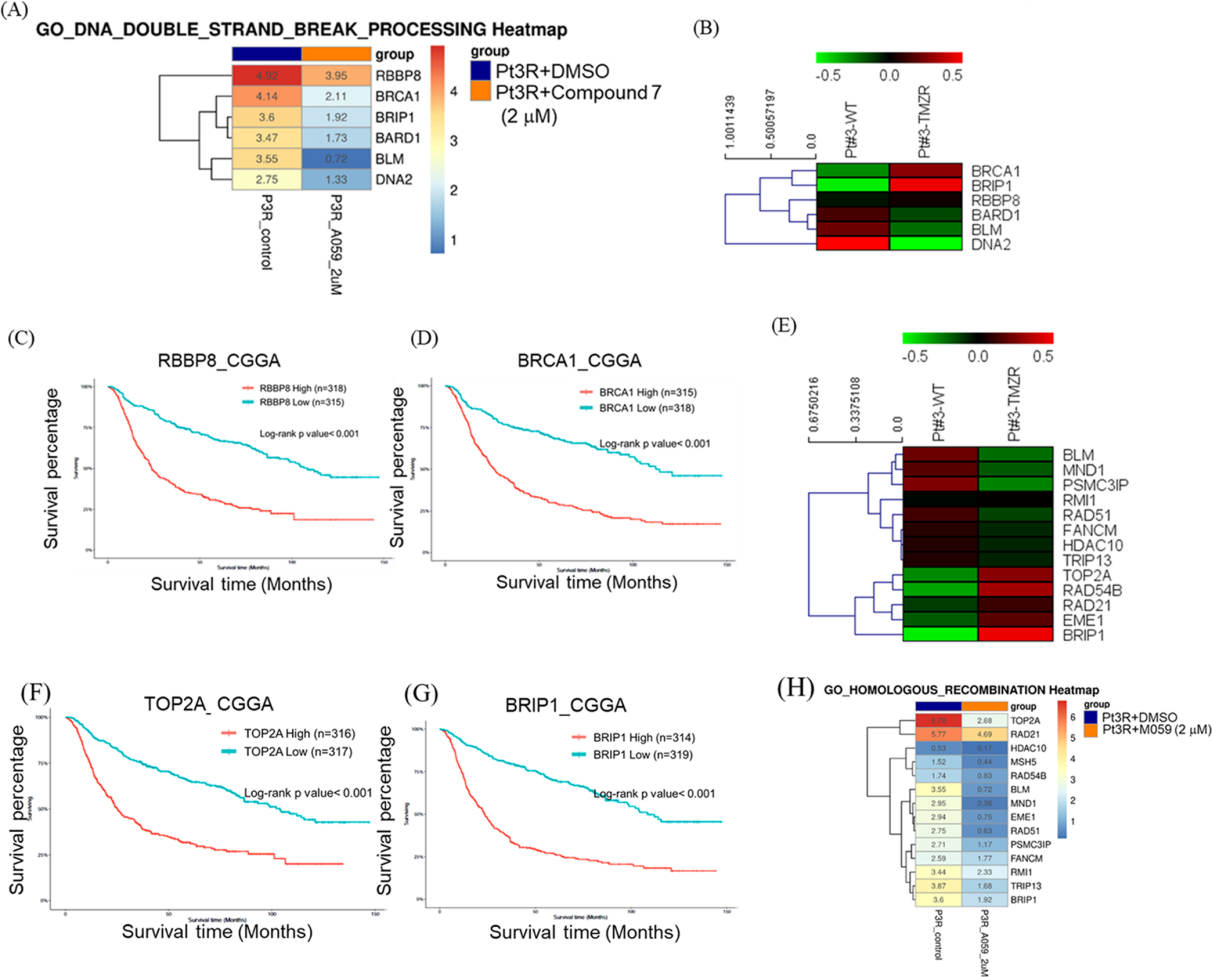
Compound **7** suppressed DNA repair-related genes. (A) Effect of compound **7** on gene expression involved in processing DNA double-strand break. (B) Comparison in gene expression between Pt3 and Pt3R cells. (C, D) Prognostic value of RBBP8 and BRCA1 in GBM patients. (E) Comparison in gene expression between Pt3 and Pt3R cells. (F, G) Prognostic value of TOP2A and BRIP1 in GBM patients. (H) Effect of compound **7** on gene expression involved in homologous recombination.

In addition to cell division, DNA repair capacity was highly suppressed by compound 7. In particular, expression of genes involved in processing DNA double-strand break and in homologous recombination was decreased by **7** (Figure 9A). Further explorations conducted to establish the expression profile of genes in TMZ-resistant Pt3R cells revealed that RB Binding Protein (RBBP8) and BRCA1 expression were increased in TMZ-resistant Pt3R cells (Figure 9B) as per the relative comparison of their expression levels in Pt3 cells. Also, these 2 genes (RBPP8 and BRCA1) were significantly correlated with poor prognosis of GBM patients (Figure 9C,D). Notably, both were suppressed by treatment with compound **7** (Figure 9A). Among the genes regulating homologous recombination, DNA topoisomerase II (TOP2A), DNA repair and recombination protein RAD54B, RAD21, crossover junction endonuclease EME1 and BRIP1 were found to be increased in TMZ-resistant Pt3R cells as compared with Pt3 cells (Figure 9E). Particularly, TOP2A and BRCA1 interacting helicase (BRIP) 1 significantly correlated with poor prognosis (Figure 9F,G). Delightfully, the genes mentioned above regulating homologous recombination were suppressed by compound 7 (Figure 9H). Taken together, these findings highlighted the potential of compound **7** to downregulate the DNA repair-related gene expression which is assumed to be responsible for the anti-GBM efficacy of compound **7**.

### Dual Inhibitor **7** Increased ROS Accumulation by Disrupting Redox Homeostasis in Mitochondria

**Figure 10:**
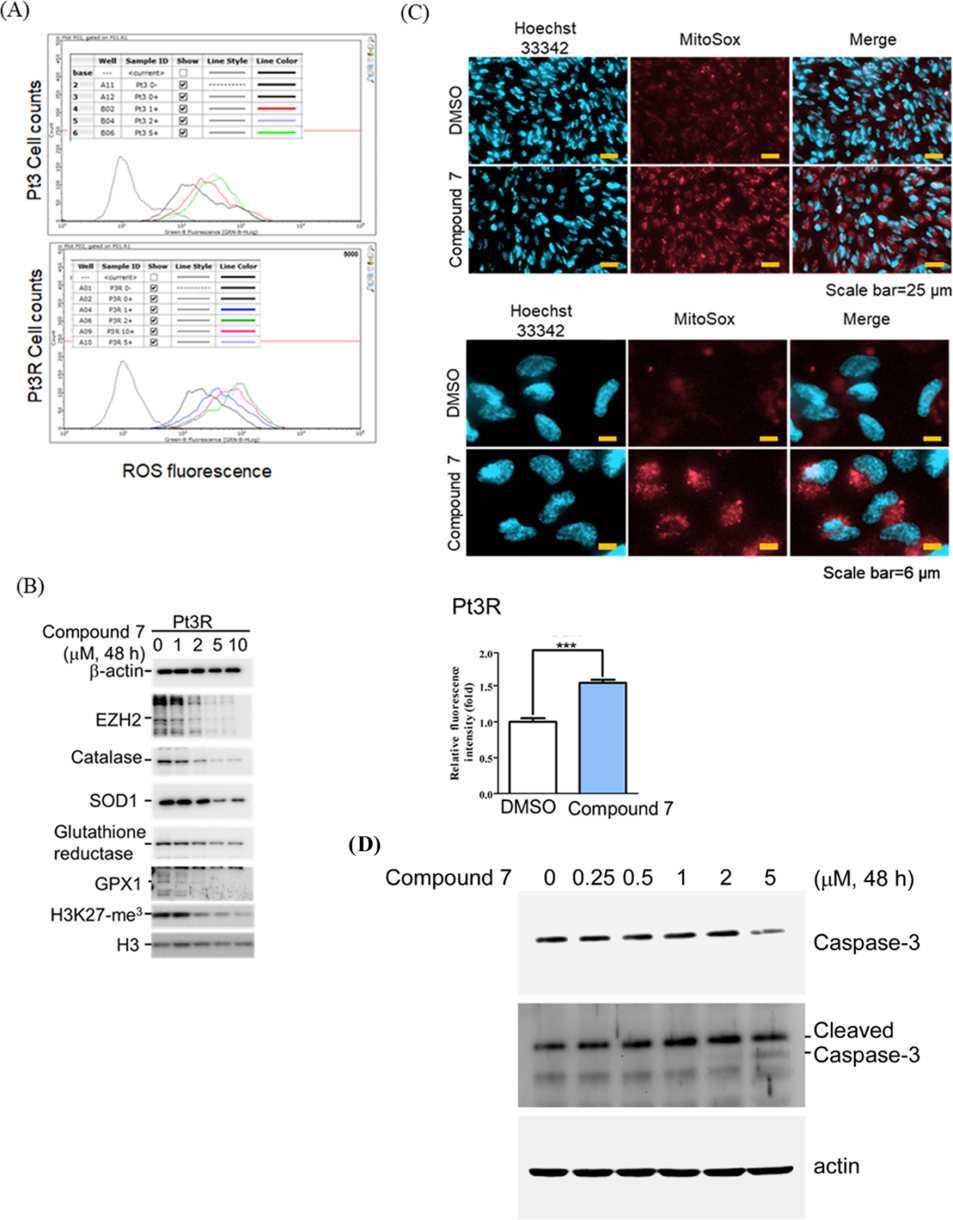
Compound **7** triggers ROS production from mitochondria. (A) After treatment for 48 h, cells were stained with the DHR reagent, and the fluorescent signal was estimated by flow cytometry. Treatment concentration of compound **7** was indicated. (B) Protein lysates were subjected to Western blotting using the indicated antibody. (C) After staining with MitoSOX (red) and Hoechst 33342 (blue), cellular signals were photographed and quantified D. Protein lysates were subjected to Western blotting using the indicated antibody.

Literature precedents revealed that TMZ-resistant GBM cells can survive in the presence of TMZ through enhanced reactive oxygen species (ROS)-cleaning capacity.^54−57^ Thus, the ability of compound **7** to disturb redox homeostasis in TMZ-resistant cells and attain amplified therapeutic efficacy for GBM was evaluated. In both Pt3 and Pt3R cells, treatment with compound **7** for 48 h increased the ROS level (Figure 10A). Moreover, proteins that are involved in cleaning ROS, including catalase, superoxide dismutase (SOD) 1, glutathione reductase, and glutathione peroxidase (GPX) 1, were also decreased by **7** (Figure 10B). In parallel, EZH2 expression and EZH2-modulated H3K27 trimethylation were also inhibited by **7**, indicating its inhibitory effect on EZH2 (Figure 10B). These results indicated that compound **7** suppressed ROS catabolism pathway, causing the death of TMZ-resistant GBM cells. Moreover, it was also observed that compound **7** increased mitochondria-derived ROS. To assess the mitochondria-derived ROS increasing potential of compound **7**, fluorescent MitoSOX labeled mitochondrial ROS was used and it was found that compound **7** remarkably enhanced the red signal representing ROS produced from mitochondria (Figure 10C). Also, compound **7** treatment was found to upregulate the expression of cleaved caspase-3 (Figure 10D).

### Dual Inhibitor **7** Exhibited Magnificent Potential to Inhibit the Growth of TMZ-Resistant GBM *In Vivo*

**Figure 11:**
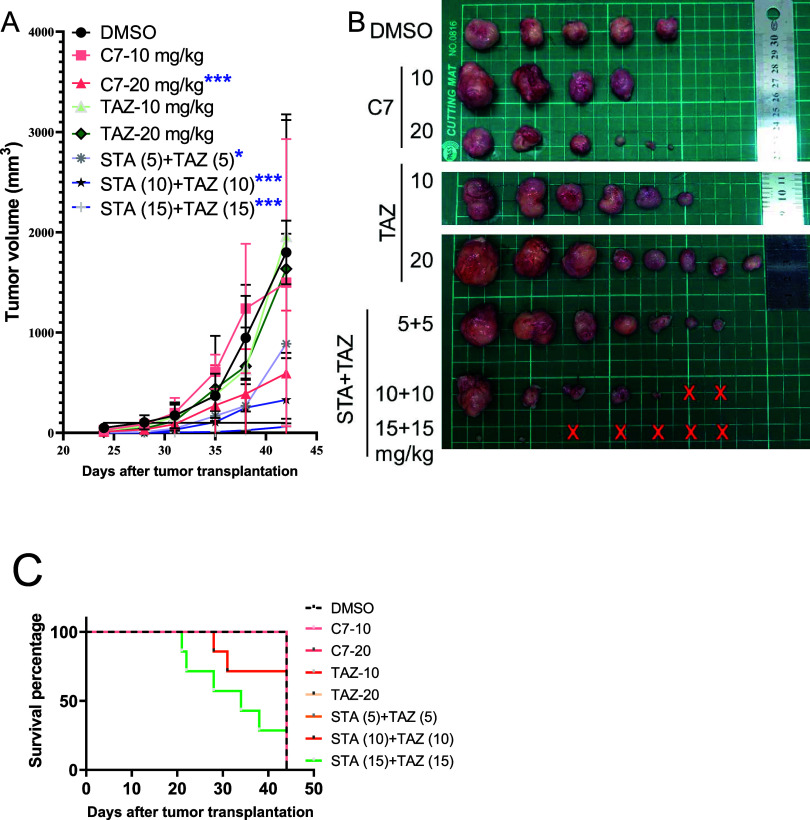
Compound **7** (20 mg/kg) significantly decreased the growth of Pt#3-R-induced tumor (A, B). TAZ failed to suppress tumor growth. Although the combination of TAZ with STA suppressed tumor growth (A, B), experimental mice that received TAZ and STA died earlier due to systemic toxicity (B, C). These results suggest that compound **7** is a potential candidate to suppress GBM growth by targeting EZH2 and HSP90 simultaneously.

*In vivo* anti-GBM efficacy of compound **7** was also evaluated in this study. For the *in vivo* study, experimental *NOD.CB17-Prkdc*^*scid*^*/NCrCrl* mice (8-week-old) were used and Pt3R cells (1 × 10^6^) in 50 μL Dulbecco’s modified Eagle’s medium (DMEM) were injected into the back of mice. After 10 days, the tumor was detectable. As shown in Figure 11, Compound **7** (20 mg/kg) significantly decreased the growth of Pt#3-R-induced tumor (Figure 11A,B); however, tazemetostat (**TAZ**) failed to suppress tumor growth (Figure 11A,B). Notably, the combination of **TAZ** with **STA** suppressed tumor growth (Figure 11A,B), experimental mice that received the cocktail (**TAZ and STA**) died earlier due to the systemic toxicity (Figure 11B,C). Pharmacokinetic data of compound **7** is shown in Table S1. In a nutshell, the outcome of the *in vivo* study ascertains that the remarkable *in vitro* anti-GBM activity profile of dual inhibitor **7** was translated to the *in vivo* potential.

## Conclusion

Numerous precedents coupled with our investigation to assess the expression level of EZH2 tissues advocated for its overexpression in GBM tissues. Also, the high expression of EZH2 significantly correlated with the shorter survival time in patients with GBM ascertaining its standing as an important oncogene in GBM pathogenesis. Intriguingly, the only FDA-approved EZH2 inhibitor, Tazemetostat, did not exhibit anti-GBM efficacy and the idea of stitching another antitumor pharmacophore to the core structure of tazemetostat was conceived as a prudent strategy to activate its chemical architecture to exert anti-GBM effects. Notably, this conceivement was predominantly attributed to the learnings leveraged from our previous dual inhibitor fabrication campaigns. The candidature of the HSP90 chaperone protein inhibitors was deemed suitable for the tetheration to the structural template of Tazemetostat, in light of numerous reports validating the efficacy of HSP90 inhibitors in GBM coupled with our preliminary investigation results confirming the possible attainment of remarkable anti-GBM efficacy through a combination of EZH2 and HSP90 inhibitors. Thus, to extract pronounced anti-GBM effects from balanced modulation of EZH2 and HSP90, hybrid templates comprising structural commonalities of EZH2 and HSP90 inhibitors were constructed via multistep synthetic routes. Encouragingly, a strikingly balanced dual inhibitor **7** was identified through the *in vitro* enzymatic assays and the impact of dual inhibition was evidenced in the cytotoxicity studies. Hybrid template **7** displayed substantial cell growth inhibitory activity against Pt3R that was assumably attributed to its dual EZH2-HSP90 inhibitory potential. Further exhaustive exploration of chemical probe **7** ascertained its ability to (i) suppress kinetochore- and DNA repair-related gene expression (ii) increase ROS accumulation through disrupting redox homeostasis in mitochondria (iii) inhibit the growth of TMZ-resistant GBM *in vivo*. Taken together, the study has resulted in identifying a tractable dual inhibitor that encompasses the requisite features of an emerging therapeutic for treatment-resistant brain tumors.

## Experimental Section

### Chemistry

Chemistry Nuclear magnetic resonance (^1^H NMR and ^13^C NMR) spectra were obtained with a Bruker DRX-500 spectrometer operating at 300 MHz. Chemical shifts are reported in parts per million (ppm, δ) downfield from TMS as an internal standard. High-resolution mass spectra (HRMS) were measured with a JEOL (JMS-700) electron impact (EI) mass spectrometer. The purity of the final compounds was determined using a Hitachi 2000 series HPLC system using C-18 column (Agilent ZORBAX Eclipse XDB-C18 5 mm. 4.6 mm 150 mm) and all compounds are >95% pure by HPLC analysis. Column chromatography was accomplished on silica gel (Merck Kieselgel 60, No. 9385, 230e400 mesh ASTM). All reactions were carried out under an atmosphere of dry nitrogen.

#### Methyl 5-Bromo-2-methyl-3-nitrobenzoate (**12**)

In a 250 mL round-bottom flask (RBF), compound 11 (5 g, 0.019 mol) and potassium carbonate (5.31 g, 0.038 mol) were dissolved in 50 mL of DMF, and iodomethane (3.54 g, 0.024 mol) was added dropwise to the reaction mixture. The resulting mixture was stirred overnight at room temperature and the progress of the reaction was monitored using thin-layer chromatography (TLC). After completion of the reaction, 100 mL of cold water was added to it and the resulting precipitates were filtered under suction, washed with water, and dried. The precipitates were carried out to the next step without further purification with a yield of 90%. ^1^H NMR (300 MHz, DMSO-*d*_6_): δ 7.31(s, 1H), 7.27 (s, 1H), 3.87 (s, 3H), 2.26 (s, 3H).

#### Methyl 3-Amino-5-bromo-2-methylbenzoate (**13**)

Compound **12** (5 g, 0.020 mol), iron powder (5.69 g, 0.102 mol), and ammonium chloride (2.17 g, 0.040 mol) were weighed in a 100 mL RBF and a mixture of ethanol and water (18:2 mL) was added as a solvent. The reaction mixture was refluxed at 100 °C for 2 h, and the progress of the reaction was monitored by TLC. After completion of the reaction, the mixture was diluted with methanol and passed through Celite. The solvent was evaporated *in vacuo*, and the residue was purified by silica gel column chromatography (ethyl acetate:hexane 1:5) with a yield of 91%. ^1^H NMR (300 MHz, CDCl_3_): δ 7.29 (d, *J* = 2.4 Hz, 1H), 6.91 (d, *J* = 2.4 Hz, 1H), 3.88 (s, 3H), 3.80 (s, 2H), 2.24 (s, 3H).

#### Methyl 5-Bromo-2-methyl-3-((tetrahydro-2*H*-pyran-4-yl)amino)benzoate (**14**)

In a 100 mL RBF, compound **13** (5 g, 0.020 mol) and tetrahydro-4*H*-pyran-4-one (4.10 g, 0.040 mol) were dissolved in 100 mL of methanol and acetic acid (1.22 g, 0.020 mol) was added in it. The mixture was stirred at room temperature for 30 min, followed by portion-wise addition of sodium cyanoborohydride (3.83 g, 0.60 mol). The reaction was refluxed overnight, and the progress of the reaction was monitored by using TLC. After completion of the reaction, the resulting precipitates were filtered under suction, washed with methanol, and dried. The compound was carried out to the next step without further purification with a percentage yield of 88%. ^1^H NMR (300 MHz, CDCl_3_): δ 7.23 (d, *J* = 1.2 Hz, 1H), 6.85 (s, 1H), 3.97–4.03 (m, 2H), 3.86 (s, 3H), 3.46–3.56 (m, 3H), 2.23 (s, 3H) 2.03 (d, *J* = 11.4 Hz, 2H), 1.48–1.54 (m, 2H)

#### Methyl 5-Bromo-3-(ethyl(tetrahydro-2*H*-pyran-4-yl)amino)-2-methylbenzoate (**15**)

In a 100 mL RBF, compound **14** (3 g, 0.009 mol), acetaldehyde (0.8 g, 0.018 mol), and acetic acid (0.54 g, 0.009 mol) were dissolved in 60 mL of dichloroethane and the reaction was stirred at room temperature for 30 min, followed by portion-wise addition of sodium triacetoxyborohydride (5.72 g, 0.027 mol) under ice cold conditions. The reaction was continued at room temperature for 5 h. After completion of the reaction (TLC), the reaction mixture was diluted with cold water (100 mL) and the compound was extracted using ethyl acetate (50 mL × 3). The combined organic layer was dried over anhydrous magnesium sulfate and concentrated under reduced pressure. The residue was further purified by performing silica gel column chromatography (ethyl acetate:hexane 1:5) with a yield of 90%; ^1^H NMR (300 MHz, CDCl_3_): δ 7.73 (d, *J* = 1.8 Hz, 1H), 7.38 (d, *J* = 1.8 Hz, 1H), 3.94–4.00 (m, 2H), 3.91 (s, 3H), 3.30–3.38 (m, 2H), 3.04–3.11 (m, 2H), 2.81–3.00 (m, 1H), 2.47 (s, 3H), 1.63–1.74 (m, 4H), 0.89 (t, *J* = 6.9 Hz, 3H).

#### Methyl 5-(Ethyl(tetrahydro-2*H*-pyran-4-yl)amino)-4-methyl-4′-nitro-[1,1′-biphenyl]-3-carboxylate (**16**)

In a 100 mL RBF, compound **15** (4 g, 0.011 mol), corresponding 4-nitrophenyl boronic acid (2.24 g, 0.015 mol), palladium triphenylphosphine (1.15 g, 0.001 mol), and sodium carbonate (3.49 g, 0.033 mol) was dissolved in 40 mL of DMF and the reaction was refluxed at 100 °C for 4 h. After completion of the reaction (TLC), the reaction mixture was diluted with water (100 mL) and the compound was extracted using ethyl acetate (50 mL × 3). The combined organic layer was dried over anhydrous magnesium sulfate and concentrated under reduced pressure. The residue was further purified by performing silica gel column chromatography (ethyl acetate:hexane 1:3) with a yield of 71%. ^1^H NMR (300 MHz, DMSO-*d*_6_): δ 8.31 (d, *J* = 7.2 Hz, 2H), 7.85 (s, 1H), 7.74 (d, *J* = 7.2 Hz, 2H), 7.52 (s, 1H), 3.93–3.95 (m, 5H), 3.02–3.39 (m, 5H), 2.57 (s, 3H), 1.69–1.74 (m, 4H), 0.95 (t, *J* = 6.9 Hz, 3H).

#### Methyl 5-(Ethyl(tetrahydro-2*H*-pyran-4-yl)amino)-4-methyl-3′-nitro-[1,1′-biphenyl]-3-carboxylate (**17**)

Intermediate **17** was synthesized with 70% yield using the same experimental procedure as that described for the synthesis of compound **16**. ^1^H NMR (300 MHz, CDCl_3_): δ 8.41 (t, *J* = 2.1 Hz, 1H), 8.21–8.25 (m, 1H), 7.91–7.93 (m, 1H), 7.86 (d, *J* = 1.8 Hz, 1H), 7.65 (t, *J* = 8.1 Hz, 1H), 7.53 (d, *J* = 1.8 Hz, 1H), 4.02 (s, 1H), 3.97 (s, 4H), 3.33–3.41 (m, 2H), 3.15–3.22 (m, 2H), 2.85–3.10 (m, 1H), 2.60 (s, 3H), 1.69–1.76 (m, 4H), 0.95 (t, *J* = 7.2 Hz, 3H).

#### Methyl 4′-Amino-5-(ethyl(tetrahydro-2*H*-pyran-4-yl)amino)-4-methyl-[1,1′-biphenyl]-3-carboxylate (**18**)

Intermediate **16** (1 g, 0.0025 mol), iron powder (0.69 g, 0.012 mol), and ammonium chloride (0.267 g, 0.005 mol) were weighed in a 100 mL round-bottom flask (RBF) and a mixture of ethanol and water (27:3 mL) was added as a solvent. The reaction mixture was refluxed at 100 °C for 2 h, and the progress of the reaction was monitored by TLC. After completion of the reaction, the reaction mixture was diluted with methanol and passed through Celite. The resulting solvent mixture was evaporated *in vacuo*, and the residue was purified by silica gel column chromatography (ethyl acetate:hexane 1:4) with a yield of 80%. ^1^H NMR (300 MHz, CD_3_OD): δ 7.22–7.26 (m, 3H), 7.08 (d, *J* = 1.5 Hz, 1H), 6.61 (d, *J* = 8.5 Hz, 2H), 3.91–3.94 (m, 5H), 3.06–3.34 (m, 5H), 2.54 (s, 3H), 1.63–1.69 (m, 4H), 0.93 (t, *J* = 6.9 Hz, 3H).

#### Methyl 3′-Amino-5-(ethyl(tetrahydro-2*H*-pyran-4-yl)amino)-4-methyl-[1,1′-biphenyl]-3-carboxylate (**19**)

Intermediate **19** was synthesized with 74% yield, employing **17** as the starting material, using the same experimental procedure as that described for the synthesis of compound **18**. ^1^H NMR (300 MHz, CDCl_3_): δ 7.83 (d, *J* = 2.1 Hz, 1H), 7.51 (s, 1H), 7.24–7.30 (m, 1H), 6.99–7.02 (m, 1H), 6.93 (t, *J* = 1.8 Hz, 1H), 6.72–6.76 (m, 1H), 3.95–4.02 (m, 2H), 3.94 (s, 3H), 3.32–3.40 (m, 2H), 3.17 (d, *J* = 6.9 Hz, 2H), 3.13 (s, 1H), 2.58 (s, 3H), 1.69–1.77 (m, 4H), 0.91–0.96 (m, 3H).

#### Methyl 4′-(2,4-Bis(benzyloxy)-5-isopropylbenzamido)-5-(ethyl(tetrahydro-2*H*-pyran-4-yl)amino)-4-methyl-[1,1′-biphenyl]-3-carboxylate (**20**)

In a 100 mL RBF, the corresponding intermediate **18** (3.2 g, 0.0086 mol), 2,4-bis(benzyloxy)-5-isopropylbenzoic acid (3.27 g, 0.0086 mol), EDC·HCl (3.3 g, 0.017 mol), and HOBT (1.7 g, 0.013 mol) dissolved in 20 mL of DMF, and DIPEA (2.7 g, 0.022 mol) were added dropwise to the reaction mixture. The reaction mixture was continued for 3 h, and the progress of the reaction was monitored by TLC. After completion, the reaction mixture was diluted with water (100 mL) and the compound was extracted using ethyl acetate (50 mL × 3). The combined organic layer was dried over anhydrous magnesium sulfate and concentrated under reduced pressure. The residue was further purified by performing silica gel column chromatography (ethyl acetate:hexane 1:3) with a yield of 65%. ^1^H NMR (300 MHz, CDCl_3_): δ 7.72 (s, 1H), 7.21–7.45 (m, 16H), 6.62 (s, 1H), 5.19 (s, 4H), 4.15 (m, 2H), 3.96 (s, 3H), 3.08–3.31 (m, 5H), 3.29 (m, 1H), 2.54 (s, 3H), 1.61–1.67 (m, 4H), 1.26 (d, *J* = 7.2 Hz, 6H), 0.95 (t, *J* = 6.9 Hz, 3H).

#### Methyl 3′-(2,4-Bis(benzyloxy)-5-isopropylbenzamido)-5-(ethyl(tetrahydro-2*H*-pyran-4-yl)amino)-4-methyl-[1,1′-biphenyl]-3-carboxylate (**21**)

Intermediate **21** was synthesized in 70% yield, employing **19** as the starting material, using the same experimental procedure as that described for the synthesis of compound **20**. ^1^H NMR (300 MHz, CDCl_3_): δ 9.97 (s, 1H), 8.24 (s, 1H), 7.72 (s, 1H), 7.19–7.53 (m, 15H), 6.67 (s, 1H), 5.20 (s, 2H), 5.18 (s, 2H), 3.96–4.15 (m, 5H), 3.33–3.41 (m, 3H), 3.14 (s, 3H), 2.59 (s, 3H), 1.72–1.76 (m, 4H), 1.27–1.32 (m, 6H), 0.91–0.96 (m, 3H).

#### 4′-(2,4-Bis(benzyloxy)-5-isopropylbenzamido)-5-(ethyl(tetrahydro-2*H*-pyran-4-yl)amino)-4-methyl-[1,1′-biphenyl]-3-carboxylic Acid (**22**)

Intermediate **20** (1 g, 0.0014 mol) was dissolved in 20 mL of dioxane and lithium hydroxide (0.29 g, 0.0068 equiv) was added slowly to the reaction mixture. The reaction was continued for 3 h and the progress of the reaction was monitored by TLC. After completion of the reaction, pH was adjusted to 5 using 3 N HCl and the resulting precipitates were filtered, washed, and dried. The resulting acid was carried to the next step without further purification with a yield of 80%.

#### 3′-(2,4-Bis(benzyloxy)-5-isopropylbenzamido)-5-(ethyl(tetrahydro-2*H*-pyran-4-yl)amino)-4-methyl-[1,1′-biphenyl]-3-carboxylic Acid (**23**)

Intermediate **23** was synthesized in 91% yield from starting material **21** like that described for the synthesis of **22**. The resulting acid was carried to the next step without further purification.

#### 4′-(2,4-Bis(benzyloxy)-5-isopropylbenzamido)-*N*-((4,6-dimethyl-2-oxo-1,2-dihydropyridin-3-yl)methyl)-5-(ethyl(tetrahydro-2*H*-pyran-4-yl)amino)-4-methyl-[1,1′-biphenyl]-3-carboxamide (**24**)

Intermediate **22** (1 g, 0.0014 mol), 3-(aminomethyl)-4,6-dimethylpyridin-2(1*H*)-one (0.21 g, 0.0014 mol), EDC·HCl (0.53 g, 0.0028 mol), and HOBT (0.28 g, 0.0021 mol) were dissolved in 10 mL of DMF and DIPEA (0.45 g, 0.0035 mol) was added dropwise to the reaction mixture. The reaction mixture was continued for 3 h and the progress of the reaction was monitored by TLC. After completion, the reaction mixture was diluted with water (100 mL) and the compound was extracted using ethyl acetate (50 mL × 3). The combined organic layer was dried over anhydrous magnesium sulfate and concentrated under reduced pressure. The residue was further purified by performing silica gel column chromatography (ethyl acetate:hexane 1:2) with a yield of 74%. ^1^H NMR (300 MHz, CD_3_OD): δ 7.79 (s, 1H), 7. 09–7.38 (m, 16H), 6.62 (s, 1H), 6.11 (s, 1H), 5.19 (s, 4H), 4.49 (s, 2H), 4.15 (m, 2H), 3.08–3.31 (m, 5H), 3.29 (s, 1H), 2.53 (s, 3H), 2.33 (s, 3H), 2.28 (s, 3H), 1.61–1.67 (m, 4H), 1.26 (d, *J* = 7.2 Hz, 6H), 0.95 (t, *J* = 6.9 Hz, 3H).

#### 3′-(2,4-Bis(benzyloxy)-5-isopropylbenzamido)-*N*-((4,6-dimethyl-2-oxo-1,2-dihydropyridin-3-yl)methyl)-5-(ethyl(tetrahydro-2*H*-pyran-4-yl)amino)-4-methyl-[1,1′-biphenyl]-3-carboxamide (**25**)

Intermediate **25** was synthesized in 82% yield from starting material **23** like that described for the synthesis of **23**. ^1^H NMR (300 MHz, CD_3_OD): δ 7.98 (s, 1H), 7.79 (s, 1H), 7.34–7.65 (m, 15 H), 6.54 (s, 1H), 6.17 (s, 1H), 5.11 (s, 2H), 5.07 (s, 2H), 4.66 (s, 2H), 3.95 (bs, 2H), 3.12–3.33 (m, 6H), 2.51 (s, 3H), 2.35 (s, 3H), 2.26 (s, 3H), 1.59–1.76 (m, 4H), 1.29 (d, *J* = 6.6 Hz, 6H), 0.97 (t, *J* = 7.2 Hz, 3H).

#### 4′-(2,4-Fihydroxy-5-isopropylbenzamido)-*N*-((4,6-dimethyl-2-oxo-1,2-dihydropyridin-3-yl)methyl)-5-(ethyl(tetrahydro-2*H*-pyran-4-yl)amino)-4-methyl-[1,1′-biphenyl]-3-carboxamide (**1**)

In a 100 mL RBF, intermediate **24** (0.5 g, 0.0006 mol) was dissolved in 20 mL of methanol and catalytic Pd/C was added. The reaction mixture was continued at room temperature for 4 h under hydrogen gas conditions and the progress of the reaction was monitored by using TLC. After completion of the reaction, the mixture was diluted with methanol and passed through Celite. The solvent was evaporated *in vacuo* and the residue was purified by silica gel column chromatography (ethyl acetate:hexane 1:1) with a yield of 50%. HPLC purity: 98.73%; mp: 166–168 °C; ^1^H NMR (600 MHz, CD_3_OD): δ 7.82 (s, 1H), 7.56 (d, *J* = 8.4 Hz, 2H), 7.47 (d, *J* = 9 Hz, 2H), 7.41 (d, *J* = 1.2 Hz, 1H), 7.23 (d, *J* = 1.8 Hz, 1H), 6.34 (s, 1H), 6.10 (s, 1H), 4.49 (s, 2H), 3.90 (d, *J* = 10.8 Hz, 2H), 3.45–3.50 (m, 1H), 3.32–3.62 (m, 2H), 3.19–3.24 (m, 1H), 3.10–3.14 (m, 2H), 2.42 (s, 3H), 2.30 (s, 3H), 2.25 (s, 3H), 1.73 (d, *J* = 12 Hz, 2H), 1.58–1.65 (m, 2H), 1.26 (d, *J* = 7.2 Hz, 6H), 0.87 (t, *J* = 7.2 Hz, 3H). ^13^C NMR (150 MHz, CD_3_OD): δ 168.23, 160.13, 159.49, 152.05, 149.21, 143.46, 138.92, 137.98, 137.42, 136.04, 132.84, 127.32, 126.53, 126.09, 123.60, 121.69, 120.65, 109.64, 107.43, 102.24, 71.79, 70.64, 66.85, 60.82, 58.45, 41.78, 35.38, 31.45, 30.47, 26.56, 21.68, 18.87, 18.33, 17.18, 13.78, 12.81, 11.73. HRMS (ESI) for C_39_H_47_N_4_O_6_ (M + H^+^): calcd; 667.3496 found, 667.3474 (M + H^+^).

#### 3′-(2,4-Fihydroxy-5-isopropylbenzamido)-*N*-((4,6-dimethyl-2-oxo-1,2-dihydropyridin-3-yl)methyl)-5-(ethyl(tetrahydro-2*H*-pyran-4-yl)amino)-4-methyl-[1,1′-biphenyl]-3-carboxamide (**2**)

Title compound **2** was synthesized in 62% yield from starting material **25** in a manner similar to that described for the synthesis of **1**. HPLC purity: 97.25%; mp: 170–172 °C; ^1^H NMR (600 MHz, CD_3_OD): δ 7.89 (s, 1H), 7.76 (s, 1H), 7.58 (dd, *J* = 8.4 and 1.2 Hz, 1H), 7.48 (d, *J* = 1.8 Hz, 1H), 7.40 (t, *J* = 7.8 Hz, 1H), 7.34 (m, 2H), 6.34 (s, 1H), 6.09 (s, 1H), 4.48 (s, 2H), 3.90 (d, *J* = 10.2 Hz, 2H), 3.33–3.36 (m, 2H), 3.09–3.21 (m, 4H), 2.38 (s, 3H), 2.32 (s, 3H), 2.22 (s, 3H), 1.74 (d, *J* = 11.4 Hz, 2H), 1.59–1.66 (m, 2H), 1.24 (d, *J* = 6.6 Hz, 6H), 0.89 (t, *J* = 7.2 Hz, 3H). ^13^C NMR (150 MHz, CD_3_OD): δ 171.65, 168.42, 164.21, 160.16, 159.54, 152.20, 149.33, 143.43, 140.88, 139.05, 138.68, 138.38, 133.19, 128.91, 127.28, 126.12, 123.99, 122.42, 121.32, 120.86, 120.36, 119.68, 109.67, 107.54, 102.31, 66.84, 58.36, 41.80, 35.19, 30.50, 26.61, 21.64, 18.30, 17.16, 13.69, 11.71. HRMS (ESI) for C_39_H_47_N_4_O_6_ [M + H^+^]: calcd; 667.3496; found, 667.3489.

#### Methyl 4′-(2,4-Bis(benzyloxy)-5-isopropyl-*N*-methylbenzamido)-5-(ethyl(tetrahydro-2*H*-pyran-4-yl)amino)-4-methyl-[1,1′-biphenyl]-3-carboxylate (**26**)

In a 100 mL RBF, the intermediate **20** (6 g, 0.0082 mol) and methyl iodide (3.49 g, 0.025 equiv) were dissolved in 30 mL of DMF and portion-wise addition of sodium hydride (0.39 g, 0.016 equiv) was done. The reaction was stirred at room temperature under nitrogen conditions for 4 h and the progress of the reaction was monitored by using TLC. After completion, the reaction mixture was diluted with water (100 mL) and the compound was extracted using ethyl acetate (50 mL × 3). The combined organic layer was dried over anhydrous magnesium sulfate and concentrated under reduced pressure. The residue was further purified by performing silica gel column chromatography (ethyl acetate:hexane 1:1) with a yield of 51%. ^1^H NMR (300 MHz, CD_3_OD): δ 7.75 (s, 1H), 7.29–7.38 (m, 16H), 6.29 (s, 1H), 5.19 (s, 2H), 5.16 (s, 2H), 4.18 (m, 2H), 3.91 (s, 3H), 3.41 (s, 3H), 3.29 (s, 1H), 3.11–3.21 (m, 5H), 2.51 (s, 3H), 1.58–1.63 (m, 4H), 1.29 (d, *J* = 7.2 Hz, 6H), 0.92 (t, *J* = 6.9 Hz, 3H).

#### Methyl 4′-(2,4-Bis(benzyloxy)-*N*-ethyl-5-isopropylbenzamido)-5-(ethyl(tetrahydro-2*H*-pyran-4-yl)amino)-4-methyl-[1,1′-biphenyl]-3-carboxylate (**27**)

Intermediate **27** was synthesized in 69% yield from starting material **20** using ethyl iodide employing the experimental procedure in a manner similar to that described for compound **26**. ^1^H NMR (300 MHz, CD_3_OD): δ 7.68 (s, 1H), 7. 32–7.39 (m, 16H), 6.59 (s, 1H), 5.11 (s, 2H), 5.09 (s, 2H), 3.98 (m, 4H), 3.87 (s, 3H), 3.11–3.27 (m, 6H), 2.46 (s, 3H), 1.58–1.63 (m, 4H), 1.29 (d, *J* = 7.2 Hz, 6H), 1.21 (t, *J* = 7.2 Hz, 3H), 0.89 (t, *J* = 6.9 Hz, 3H).

#### Methyl 4′-(2,4-Bis(benzyloxy)-5-isopropyl-*N*-propylbenzamido)-5-(ethyl(tetrahydro-2*H*-pyran-4-yl)amino)-4-methyl-[1,1′-biphenyl]-3-carboxylate (**28**)

Intermediate **28** was synthesized in 58% yield from starting material **20** using propyl iodide like that described for compound **26**. ^1^H NMR (300 MHz, CD_3_OD): δ 7.59 (s, 1H), 7. 32–7.39 (m, 16H), 6.64 (s, 1H), 5.14 (s, 2H), 5.11 (s, 2H), 3.88–3.92 (m, 4H), 3.89 (s, 3H), 3.17–3.22 (m, 6H), 2.41 (s, 3H), 1.47–1.62 (m, 6H), 1.29 (d, *J* = 7.2 Hz, 6H), 1.18 (t, *J* = 7.2 Hz, 3H), 0.94 (t, *J* = 6.9 Hz, 3H).

#### Methyl′-(*N*-Benzyl-2,4-bis(benzyloxy)-5-isopropylbenzamido)-5-(ethyl(tetrahydro-2*H*-pyran-4-yl)amino)-4-methyl-[1,1′-biphenyl]-3-carboxylate (**29**)

Intermediate **29** was synthesized in 65% yield from starting material **20** using benzyl iodide in a manner similar to that described for compound **26**. ^1^H NMR (300 MHz, CD_3_OD): δ 7.52 (s, 1H), 7.24–7.41 (m, 21H), 6.62 (s, 1H), 5.11 (s, 2H), 5.08 (s, 2H), 5.04 (s, 2H), 3.93 (m, 2H), 3.91 (s, 3H), 3.11–3.20 (m, 6H), 2.39 (s, 3H), 1.43–1.57 (m, 4H), 1.29 (d, *J* = 7.2 Hz, 6H), 0.94 (t, *J* = 6.9 Hz, 3H).

#### Methyl 3′-(2,4-Bis(benzyloxy)-5-isopropyl-*N*-methylbenzamido)-5-(ethyl(tetrahydro-2*H*-pyran-4-yl)amino)-4-methyl-[1,1′-biphenyl]-3-carboxylate (**30**)

Intermediate **30** was synthesized in 70% yield from starting material **21** like that described for the synthesis of compound **26**. ^1^H NMR (300 MHz, CDCl_3_): δ 7.46 (s, 1H), 7.13–7.28 (m, 14H), 6.90–6.96 (m, 2H), 6.20 (s, 1H), 4.83 (s, 2H), 4.79 (s, 2H), 3.85 (m, 2H), 3.81 (s, 3H), 3.38 (s, 3H), 2.88–3.20 (m, 6H), 2.42 (s, 3H), 1.55–1.67 (m, 4H), 0.95 (d, *J* = 5.7 Hz, 6H), 0.88 (t, *J* = 3.6 Hz, 3H).

#### Methyl 3′-(2,4-Bis(benzyloxy)-*N*-ethyl-5-isopropylbenzamido)-5-(ethyl(tetrahydro-2*H*-pyran-4-yl)amino)-4-methyl-[1,1′-biphenyl]-3-carboxylate (**31**)

Intermediate **31** was synthesized in 74% yield from starting material **21** using ethyl iodide in a manner similar to that described for the synthesis of compound **26**. ^1^H NMR (300 MHz, CDCl_3_): δ 7.49 (s, 1H), 7.17–7.22 (m, 14H), 6.93–6.95 (m, 2H), 6.16 (s, 1H), 4.87 (s, 2H), 4.71 (s, 2H), 3.98 (m, 4H), 3.91 (s, 3H), 3.15–3.32 (m, 6H), 2.42 (s, 3H), 1.51–1.68 (m, 4H), 1.32 (d, *J* = 7.2 Hz, 6H), 1.34 (t, *J* = 7.2 Hz, 3H), 0.91 (t, *J* = 6.9 Hz, 3H).

#### Methyl 3′-(2,4-Bis(benzyloxy)-5-isopropyl-*N*-propylbenzamido)-5-(ethyl(tetrahydro-2*H*-pyran-4-yl)amino)-4-methyl-[1,1′-biphenyl]-3-carboxylate (**32**)

Intermediate **32** was synthesized in 62% yield from starting material **21** using propyl iodide like that described for the synthesis of compound **26**. ^1^H NMR (300 MHz, CDCl_3_): δ 7.44 (s, 1H), 7.19–7.25 (m, 14H), 6.97–6.99 (m, 2H), 6.21 (s, 1H), 4.97 (s, 2H), 4.95 (s, 2H), 3.95–3. 97 (m, 4H), 3.89 (s, 3H), 3.21–3.29 (m, 6H), 2.49 (s, 3H), 1.52–1.67 (m, 6H), 1.31 (d, *J* = 7.2 Hz, 6H), 1.21 (t, *J* = 7.2 Hz, 3H), 0.97 (t, *J* = 6.9 Hz, 3H).

#### Methyl 3′-(*N*-Benzyl-2,4-bis(benzyloxy)-5-isopropylbenzamido)-5-(ethyl(tetrahydro-2*H*-pyran-4-yl)amino)-4-methyl-[1,1′-biphenyl]-3-carboxylate (**33**)

Intermediate **33** was synthesized in 69% yield from starting material **21** using benzyl bromide in a manner similar to that described for compound **26**. ^1^H NMR (300 MHz, CDCl_3_): δ 7.42 (s, 1H), 7.21–7.28 (m, 19H), 6.98–7.01 (m, 2H), 6.25 (s, 1H), 5.01 (s, 2H), 4.98 (s, 2H), 4.95 (s, 2H), 3. 99 (m, 2H), 3.89 (s, 3H), 3.18–3.29 (m, 6H), 2.39 (s, 3H), 1.49–1.51 (m, 4H), 1.32 (d, *J* = 7.2 Hz, 6H), 0.98 (t, *J* = 6.9 Hz, 3H).

#### 4′-(2,4-Bis(benzyloxy)-5-isopropyl-*N*-methylbenzamido)-5-(ethyl(tetrahydro-2*H*-pyran-4-yl)amino)-4-methyl-[1,1′-biphenyl]-3-carboxylic Acid (**34**)

Intermediate **26** (6 g, 0.0081 mol) was dissolved in 30 mL of dioxane and lithium hydroxide solution (1.66 g, 0.04 mol) was added dropwise to the reaction mixture. The reaction was continued for 3 h at room temperature and the progress of the reaction was monitored using TLC. After completion of the reaction, pH was adjusted to 5 using 3 N HCl and the resulting precipitates were filtered, washed, and dried. The resulting acid was carried to the next step without further purification with a yield of 79%.

#### 4′-(2,4-Bis(benzyloxy)-*N*-ethyl-5-isopropylbenzamido)-5-(ethyl(tetrahydro-2*H*-pyran-4-yl)amino)-4-methyl-[1,1′-biphenyl]-3-carboxylic Acid (**35**)

Intermediate **35** was synthesized in 56% yield from starting material **27** like that described for compound **34**. The carboxylic acid was used for the next step without purification.

#### 4′-(2,4-Bis(benzyloxy)-5-isopropyl-*N*-propylbenzamido)-5-(ethyl(tetrahydro-2*H*-pyran-4-yl)amino)-4-methyl-[1,1′-biphenyl]-3-carboxylic Acid (**36**)

Intermediate **36** was synthesized in 49% yield from starting material **28** like that described for compound **34**. The carboxylic acid was used for the next step without purification.

#### 4′-(*N*-Benzyl-2,4-bis(benzyloxy)-5-isopropylbenzamido)-5-(ethyl(tetrahydro-2*H*-pyran-4-yl)amino)-4-methyl-[1,1′-biphenyl]-3-carboxylic Acid (**37**)

Intermediate **37** was synthesized in 57% yield from starting material **29** like that described for compound **34**. The carboxylic acid was used for the next step without purification.

#### 3′-(2,4-Bis(benzyloxy)-5-isopropyl-*N*-methylbenzamido)-5-(ethyl(tetrahydro-2*H*-pyran-4-yl)amino)-4-methyl-[1,1′-biphenyl]-3-carboxylic Acid (**38**)

Intermediate **38** was synthesized in 48% yield from starting material **30** like that described for compound **34**. The carboxylic acid was used for the next step without purification.

#### 3′-(2,4-Bis(benzyloxy)-*N*-ethyl-5-isopropylbenzamido)-5-(ethyl(tetrahydro-2*H*-pyran-4-yl)amino)-4-methyl-[1,1′-biphenyl]-3-carboxylic Acid (**39**)

Intermediate **39** was synthesized in 71% yield from starting material **31** like that described for compound **34**. The carboxylic acid was used for the next step without purification.

#### 3′-(2,4-Bis(benzyloxy)-5-isopropyl-*N*-propylbenzamido)-5-(ethyl(tetrahydro-2*H*-pyran-4-yl)amino)-4-methyl-[1,1′-biphenyl]-3-carboxylic Acid (**40**)

Intermediate **40** was synthesized in 43% yield from starting material **32** in a manner similar to compound **34**. The carboxylic acid was used for the next step without purification.

#### 3′-(*N*-Benzyl-2,4-bis(benzyloxy)-5-isopropylbenzamido)-5-(ethyl(tetrahydro-2*H*-pyran-4-yl)amino)-4-methyl-[1,1′-biphenyl]-3-carboxylic Acid (**41**)

Intermediate **41** was synthesized in 55% yield from starting material **33** in a manner similar to that described for compound **34**. The carboxylic acid was used for the next step without purification

#### 4′-(2,4-Bis(benzyloxy)-5-isopropyl-*N*-methylbenzamido)-*N*-((4,6-dimethyl-2-oxo-1,2-dihydropyridin-3-yl)methyl)-5-(ethyl(tetrahydro-2*H*-pyran-4-yl)amino)-4-methyl-[1,1′-biphenyl]-3-carboxamide (**42**)

In a 100 mL RBF, the intermediate **34** (5.3 g, 0.0073 mol), 3-(aminomethyl)-4,6-dimethylpyridin-2(1*H*)-one (1.37 g, 0.0073 mol), EDC·HCl (2.79 g, 0.0145 mol), and HOBT (1.48 g, 0.011 mol) were dissolved in 30 mL of DMF, and DIPEA (2.35 g, 0.018 mol) was added dropwise in the reaction mixture. The reaction was continued for 3 h, and the progress was monitored by TLC. After completion, the reaction mixture was diluted with water (100 mL) and the compound was extracted using ethyl acetate (50 mL × 3). The combined organic layer was dried over anhydrous magnesium sulfate and concentrated under reduced pressure. The residue was further purified by performing silica gel column chromatography (ethyl acetate:hexane 1:1) with a yield of 62%. ^1^H NMR (300 MHz, CD_3_OD): δ 7.73 (s, 1H), 7. Eleven -7.29 (m, 16H), 6.66 (s, 1H), 6.15 (s, 1H), 5.23 (s, 2H), 5.15 (s. 2H), 4.53 (s, 2H), 3.99 (q, *J* = 6.6 Hz, 2H), 3.31 (m, 1H), 3.11–3.21 (m, 5H), 2.49 (s, 3H), 2.31 (s, 3H), 2.22 (s, 3H), 1.66–1.71 (m, 4H), 1.23 (d, *J* = 7.2 Hz, 6H), 0.91 (t, *J* = 6.9 Hz, 3H).

#### 4′-(2,4-Bis(benzyloxy)-*N*-ethyl-5-isopropylbenzamido)-*N*-((4,6-dimethyl-2-oxo-1,2-dihydropyridin-3-yl)methyl)-5-(ethyl(tetrahydro-2*H*-pyran-4-yl)amino)-4-methyl-[1,1′-biphenyl]-3-carboxamide (**43**)

The intermediate **43** was obtained in 56% yield from starting material **35** in a manner similar to that described for the synthesis of compound **42**. ^1^H NMR (300 MHz, CD_3_OD): δ 7.69 (s, 1H), 7.22–7.38 (m, 16H), 6.51 (s, 1H), 6.11 (s, 1H), 5.15 (s, 2H), 5.11 (s, 2H), 4.51 (bs, 2H) 3.91–3.95 (m, 4H), 3.29 (s, 3H), 3.15–3.25 (m, 6H), 2.49 (s, 3H), 2.32 (s, 3H), 2.24 (s, 3H), 1.63–1.69 (m, 4H), 1.31 (d, *J* = 7.2 Hz, 6H), 1.25 (t, *J* = 7.2 Hz, 3H), 0.92 (t, *J* = 6.9 Hz, 3H).

#### 4′-(2,4-Bis(benzyloxy)-5-isopropyl-*N*-propylbenzamido)-*N*-((4,6-dimethyl-2-oxo-1,2-dihydropyridin-3-yl)methyl)-5-(ethyl(tetrahydro-2*H*-pyran-4-yl)amino)-4-methyl-[1,1′-biphenyl]-3-carboxamide (**44**)

The intermediate **44** was obtained in 58% yield from starting material **36** in a manner similar to that described for the synthesis of compound **42.**^1^H NMR (300 MHz, CD_3_OD): δ 7.61 (s, 1H), 7. 32–7.39 (m, 16H), 6.68 (s, 1H), 6.12 (s, 1H), 5.17 (s, 2H), 5.18 (s, 2H), 4.43 (bs, 2H), 3.92–3.95 (m, 4H), 3.21–3.29 (m, 6H), 2.49 (s, 3H), 2.31 (s, 3H), 2.25 (s, 3H), 1.45–1.67 (m, 6H), 1.31 (d, *J* = 7.2 Hz, 6H), 1.16 (t, *J* = 7.2 Hz, 3H), 0.92 (t, *J* = 6.9 Hz, 3H).

#### 4′-(*N*-Benzyl-2,4-bis(benzyloxy)-5-isopropylbenzamido)-*N*-((4,6-dimethyl-2-oxo-1,2-dihydropyridin-3-yl)methyl)-5-(ethyl(tetrahydro-2*H*-pyran-4-yl)amino)-4-methyl-[1,1′-biphenyl]-3-carboxamide (**45**)

The intermediate **45** was obtained in 60% yield from starting material **37** in a manner similar to that described for the synthesis of compound **42.**^1^H NMR (300 MHz, CD_3_OD): δ 7.56 (s, 1H), 7.21–7.38 (m, 22H), 6.64 (s, 1H), 5.13 (s, 2H), 5.09 (s, 2H), 5.08 (s, 2H), 4.51 (bs, 2H), 3.92 (m, 2H), 3.15–3.25 (m, 6H), 2.49 (s, 3H), 2.29 (s, 3H), 2.21 (s, 3H), 1.48–1.54 (m, 4H), 1.25 (d, *J* = 7.2 Hz, 6H), 0.91 (t, *J* = 6.9 Hz, 3H).

#### 3′-(2,4-Bis(benzyloxy)-5-isopropyl-*N*-methylbenzamido)-*N*-((4,6-dimethyl-2-oxo-1,2-dihydropyridin-3-yl)methyl)-5-(ethyl(tetrahydro-2*H*-pyran-4-yl)amino)-4-methyl-[1,1′-biphenyl]-3-carboxamide (**46**)

The intermediate **46** was obtained in 63% yield from starting material **38** in a manner similar to that described for the synthesis of compound **42.**^1^H NMR (300 MHz, DMSO-*d*_6_) δ 11.46 (s, 1H), 8.20 (s, 1H), 7.33–7.45 (m, 13H), 7.21 (s, 1H), 7.09 (d, *J* = 7.2 Hz, 1H), 7.04 (s, 1H), 6.88 (s, 1H), 6.77 (s, 1H), 5.85 (s, 1H), 5.11 (s, 2H), 5.05 (s, 2H), 4.33 (d, *J* = 4.5 Hz, 2H), 3.81 (d, *J* = 10.5 Hz, 2H), 3.22 (t, *J* = 10.8 Hz, 2H), 3.04 (m, 4H), 2.22 (d, *J* = 4.2 Hz, 5H), 2.10 (s, 3H), 2.07 (s, 3H), 1.62 (d, *J* = 11.1 Hz, 2H), 1.52 (d, *J* = 11.1 Hz, 2H), 1.25 (s, 2H), 0.93 (s, 5H), 0.79 (t, *J* = 6.6 Hz, 3H),

#### 3′-(2,4-Bis(benzyloxy)-*N*-ethyl-5-isopropylbenzamido)-*N*-((4,6-dimethyl-2-oxo-1,2-dihydropyridin-3-yl)methyl)-5-(ethyl(tetrahydro-2*H*-pyran-4-yl)amino)-4-methyl-[1,1′-biphenyl]-3-carboxamide (**47**)

The intermediate **47** was obtained in 36% yield from starting material **39** in a manner similar to that described for the synthesis of compound **42.**^1^H NMR (300 MHz, CD_3_OD): δ 7.51 (s, 1H), 7.19–7.23 (m, 14H), 6.94 (m, 2H), 6.16 (s, 1H), 6.09 (s, 1H), 5.03 (s, 2H), 5.01 (s, 2H), 4.68 (s, 2H), 3.95–3.98 (m, 4H), 3.89 (s, 3H), 3.19–3.21 (m, 6H), 2.49 (s, 3H), 2.33 (s, 3H), 2.21 (s, 3H), 1.58–1.71 (m, 4H), 1.38 (d, *J* = 7.2 Hz, 6H), 1.31 (t, *J* = 7.2 Hz, 3H), 0.89 (t, *J* = 6.9 Hz, 3H).

#### 3′-(2,4-Bis(benzyloxy)-5-isopropyl-*N*-propylbenzamido)-*N*-((4,6-dimethyl-2-oxo-1,2-dihydropyridin-3-yl)methyl)-5-(ethyl(tetrahydro-2*H*-pyran-4-yl)amino)-4-methyl-[1,1′-biphenyl]-3-carboxamide (**48**)

Intermediate **48** was synthesized in 62% yield from starting material **40** in a manner similar to that described for compound **42.**^1^H NMR (300 MHz, CDCl_3_): δ 7.49 (s, 1H), 7.21–7.27 (m, 14H), 6.94–6.97 (m, 2H), 6.11 (s, 1H), 4.97 (s, 2H), 4.95 (s, 2H), 4.51 (d, *J* = 5.4 Hz, 2H), 3.96–3. 99 (m, 4H), 3.91 (s, 3H), 3.25–3.31 (m, 6H), 2.51 (s, 3H), 2.31 (s, 3H), 2.21 (s, 3H), 1.55–1.72 (m, 6H), 1.23 (d, *J* = 7.2 Hz, 6H), 1.21 (t, *J* = 7.2 Hz, 3H), 0.97 (t, *J* = 6.9 Hz, 3H).

#### 3′-(*N*-Benzyl-2,4-bis(benzyloxy)-5-isopropylbenzamido)-*N*-((4,6-dimethyl-2-oxo-1,2-dihydropyridin-3-yl)methyl)-5-(ethyl(tetrahydro-2*H*-pyran-4-yl)amino)-4-methyl-[1,1′-biphenyl]-3-carboxamide (**49**)

Intermediate **49** was synthesized in 38% yield from starting material **41** in a manner similar to that described for compound **42**. ^1^H NMR (300 MHz, CDCl_3_): δ 7.51 (s, 1H), 7.28–7.31 (m, 19H), 7.03 (m, 2H), 6.27 (s, 1H), 5.02 (s, 2H), 4.99 (s, 2H), 4.97 (s, 2H), 4.44 (d, *J* = 5.4 Hz, 2H), 4.01 (s, 2H), 3.92 (s, 3H), 3.21–3.25 (m, 6H), 2.41 (s, 3H), 2.31 (s, 3H), 2.25 (s, 3H), 1.52–1.56 (m, 4H), 1.29 (d, *J* = 7.2 Hz, 6H), 0.99 (t, *J* = 6.9 Hz, 3H).

#### 4′-(2,4-Dihydroxy-5-isopropyl-*N*-methylbenzamido)-*N*-((4,6-dimethyl-2-oxo-1,2-dihydropyridin-3-yl)methyl)-5-(ethyl(tetrahydro-2*H*-pyran-4-yl)amino)-4-methyl-[1,1′-biphenyl]-3-carboxamide (**3**)

In a 100 mL RBF, intermediate **42** (0.6 g, 0.0007 mol) was dissolved in 20 mL of methanol and catalytic Pd/C was added. The reaction was continued at room temperature for 4 h, under hydrogen gas conditions and the progress of the reaction was monitored by using TLC. After completion, the mixture was diluted with methanol and passed through Celite. The solvent was evaporated *in vacuo*, and the residue was purified by silica gel column chromatography (ethyl acetate:hexane 1:1) with yields of 47%. HPLC purity: 96.16%; mp: 184–186 °C; ^1^H NMR (600 MHz, DMSO-*d*_6_): δ 11.43 (s, 1H), 10.69 (s, 1H), 9.72 (s, 1H), 8.15 (t, *J* = 5.4 Hz, 1H), 7.54 (d, *J* = 8.4 Hz, 2H), 7.27 (d, *J* = 1.2 Hz, 1H), 7.23 (d, *J* = 8.4 Hz, 2H), 7.14 (d, *J* = 1.8 Hz, 1H), 6.51 (s, 1H), 6.18 (s,1H), 5.82 (s, 1H), 4.25 (d, *J* = 5.4 Hz, 2H), 3.79 (d, *J* = 10.2 Hz, 2H), 3.33 (s, 3H), 3.21 (t, *J* = 10.8 Hz, 2H), 3.01–3.04 (m, 2H), 2.95–3.00 (m, 1H), 2.77–2.82 (m, 1H), 2.21 (s, 3H), 2.17 (s, 3H), 2.07 (s, 3H), 1.62 (d, *J* = 12.6 Hz, 2H), 1.44–1.51 (m, 2H), 0.78 (t, *J* = 7.2 Hz, 3H), 0.68 (d, *J* = 6.6 Hz, 6H). ^13^C NMR (150 MHz, DMSO-*d*_6_): δ 170.76, 170.67, 169.39, 163.44, 158.22, 157.97, 149.94, 149.27, 144.84, 143.18, 140.06, 138.55, 136.85, 133.30, 128.22, 127.84, 127.43, 125.09, 123.47, 122.05, 121.21, 109.76, 107.78, 102.72, 66.75, 60.18, 58.14, 41.56, 38.47, 35.29, 30.69, 25.46, 22.64, 21.19, 19.36, 18.62, 14.96, 14.52, 12.98. HRMS (ESI) for C_40_H_49_N_4_O_6_ [M + H^+^]: calcd; 681.3652 found, 681.3625.

#### *N*-((4,6-Dimethyl-2-oxo-1,2-dihydropyridin-3-yl)methyl)-5-(ethyl(tetrahydro-2*H*-pyran-4-yl)amino)-4′-(*N*-ethyl-2,4-dihydroxy-5-isopropylbenzamido)-4-methyl-[1,1′-biphenyl]-3-carboxamide (**4**)

The title compound **4** was synthesized in 42% yield from starting material **43** in a manner similar to that described for compound **3**. HPLC purity: 98.38%; mp: 191–193 °C; ^1^H NMR (600 MHz, DMSO-*d*_6_): δ 11.44 (s, 1H), 10.89 (s, 1H), 9.74 (s, 1H), 8.15 (t, *J* = 4.8 Hz, 1H), 7.56 (d, *J* = 8.4 Hz, 2H), 7.28 (d, *J* = 1.2 Hz, 1H), 7.21 (d, *J* = 8.4 Hz, 2H), 7.15 (d, *J* = 1.2 Hz, 1H), 6.48 (s, 1H), 6.18 (s,1H), 5.82 (s, 1H), 4.26 (d, *J* = 4.8 Hz, 2H), 3.81–3.85 (m, 2H), 3.79 (d, *J* = 10.2 Hz, 2H), 3.21 (t, *J* = 11.4 Hz, 2H), 3.01–3.05 (m, 2H), 2.77 (t, *J* = 6.6 Hz, 1H), 2.21 (s, 3H), 2.17 (s, 3H), 2.07 (s, 3H), 1.56 (d, *J* = 12 Hz, 2H), 1.44–1.51 (m, 2H), 1.09 (t, *J* = 7.2 Hz, 4H), 0.78 (t, *J* = 7.2 Hz, 3H), 0.66 (d, *J* = 6.6 Hz, 6H). ^13^C NMR (150 MHz, DMSO-*d*_6_): δ 170.39, 169.39, 163.45, 158.06, 149.96, 149.28, 143.19, 143.08, 140.05, 138.82, 136.83, 133.36, 128.40, 128.26, 127.93, 125.03, 123.51, 122.05, 121.26, 107.80, 102.71, 66.75, 58.14, 45.42, 41.58, 35.29, 30.70, 25.43, 23.16, 22.62, 19.36, 18.62, 14.97, 13.05, 12.97. HRMS (ESI) for C_41_H_51_N_4_O_6_ [M + H^+^]: calcd; 695.3809; found, 695.3757 (M + H^+^).

#### 4′-(2,4-Dihydroxy-5-isopropyl-*N*-propylbenzamido)-*N*-((4,6-dimethyl-2-oxo-1,2-dihydropyridin-3-yl)methyl)-5-(ethyl(tetrahydro-2*H*-pyran-4-yl)amino)-4-methyl-[1,1′-biphenyl]-3-carboxamide (**5**)

The title compound **5** was synthesized in 55% yield from starting material **44** in a manner similar to that described for compound **3**. HPLC purity: 95.75%; mp: 155–157 °C; ^1^H NMR (600 MHz, CD_3_OD): δ 7.54 (d, *J* = 8.4 Hz, 2H), 7.38 (s, 1H), 7.26 (s, 1H), 7.21 (d, *J* = 7.8 Hz, 2H), 6.55 (s, 1H), 6.19 (s, 1H), 6.10 (s, 1H), 4.48 (s, 2H), 3.87–3.92 (m, 4H), 3.35 (t, *J* = 11.4 Hz, 2H), 3.11–3.14 (m, 2H), 3.06–3.09 (m, 1H), 2.83–2.88 (m, 1H), 2.38 (s, 3H), 2.31 (s, 3H), 2.23 (s, 3H), 1.73 (d, *J* = 12 Hz, 2H), 1.60–1.68 (m, 4H), 0.94 (t, *J* = 7.8 Hz, 3H), 0.87 (t, *J* = 7.2 Hz, 3H), 0.70 (d, *J* = 6.6 Hz, 6H). ^13^C NMR (150 MHz, CD_3_OD): δ 171.53, 164.21, 159.03, 158.12, 152.14, 149.29, 143.44, 143.41, 139.17, 139.08, 137.66, 133.27, 128.17, 127.71, 127.55, 125.36, 124.02, 121.33, 120.73, 109.63, 108.62, 101.89, 66.83, 58.27, 51.88, 41.70, 35.16, 30.42, 25.35, 21.46, 20.41, 18.27, 17.14, 13.62, 11.59, 10.22. HRMS (ESI) for C_42_H_53_N_4_O_6_ [M + H^+^]: calcd; 709.3965; found, 709.3976.

#### 4′-(*N*-Benzyl-2,4-dihydroxy-5-isopropylbenzamido)-*N*-((4,6-dimethyl-2-oxo-1,2-dihydropyridin-3-yl)methyl)-5-(ethyl(tetrahydro-2*H*-pyran-4-yl)amino)-4-methyl-[1,1′-biphenyl]-3-carboxamide (**6**)

Intermediate **45** (0.5 g, 0.0006 mol) was dissolved in 20 mL of DCM and 2.3 mL of 1 M BCl_3_ solution in DCM (0.27 g, 0.0023 mol) was added dropwise to the solution. The reaction mixture was stirred for 2 h and the progress of the reaction was monitored. After completion (TLC), the reaction mixture was quenched with water and extraction was done with DCM (50 mL × 3). The separated organic layer was evaporated using a rotary evaporator. The residue was purified by performing silica gel column chromatography (ethyl acetate:hexane 2:1) to give the target compound **6** in 51% yield. HPLC purity: 97.24%; mp: 135–137 °C; ^1^H NMR (600 MHz, DMSO-*d*_6_): δ 11.41 (s, 1H), 10.54 (s, 1H), 8.10 (t, *J* = 4.8 Hz, 1H), 7.46 (d, *J* = 8.4 Hz, 2H), 7.27–7.30 (m, 4H), 7.24 (s, 2H), 7.19 (t, *J* = 7.2 Hz, 1H), 7.13 (d, *J* = 8.4.2 Hz, 2H), 7.10 (d, *J* = 1.8 Hz, 1H), 6.57 (s,1H), 6.20 (s, 1H), 5.81 (s, 1H), 5.07 (s, 2H), 4.24 (d, *J* = 4.8 Hz, 2H), 3.77 (d, *J* = 10.8 Hz, 2H), 3.34 (m, 1H), 3.19 (t, *J* = 10.8 Hz, 2H), 2.98–3.02 (m, 2H), 2.77–2.82 (m, 1H), 2.19 (s, 3H), 2.15 (s, 3H), 2.07 (s, 3H), 1.59 (d, *J* = 11.4 Hz, 2H), 1.42–1.46 (m, 2H), 0.75 (t, *J* = 7.8 Hz, 3H), 0.69 (d, *J* = 6.6 Hz, 6H). ^13^C NMR (150 MHz, DMSO-*d*_6_): δ 170.71, 169.36, 163.42, 157.97, 149.92, 149.27, 143.17, 139.99, 138.35, 137.99, 136.64, 133.33, 128.79, 128.16, 127.95, 127.53, 125.20, 123.39, 122.04, 121.17, 107.75, 102.74, 72.44, 70.41, 66.73, 60.69, 58.11, 53.25, 35.28, 31.79, 30.71, 29.45, 25.52, 22.88, 22.65, 22.52, 19.36, 19.26, 18.62, 14.96, 14.23, 12.97. HRMS (ESI) for C_46_H_53_N_4_O_6_ [M + H^+^]: calcd; 757.3965; found, 757.3932.

#### 3′-(2,4-Dihydroxy-5-isopropyl-*N*-methylbenzamido)-*N*-((4,6-dimethyl-2-oxo-1,2-dihydropyridin-3-yl)methyl)-5-(ethyl(tetrahydro-2*H*-pyran-4-yl)amino)-4-methyl-[1,1′-biphenyl]-3-carboxamide (**7**)

The title compound **7** was synthesized in 48% yield from starting material **46** in a manner similar to that described for compound **3**. HPLC purity: 95.14%; mp: 161–163 °C; ^1^H NMR (600 MHz, CD_3_OD): δ 7.46 (t, *J* = 7.8 Hz, 1H), 7.39 (d, *J* = 7.8 Hz, 1H), 7.29 (d, *J* = 6.6 Hz, 1H), 7.16 (d, *J* = 1.8 Hz, 1H), 7.11 (t, *J* = 1.8 Hz, 1H), 6.98 (d, *J* = 1.8 Hz, 1H), 6.57 (s, 1H), 6.27 (s,1H), 6.10 (s, 1H), 4.47 (s, 2H), 3.90 (d, *J* = 10.8 Hz, 2H), 3.47 (s, 3H), 3.33–3.37 (m, 2H), 3.02–3.06 (m, 2H), 2.37 (s, 3H), 2.28 (s, 3H), 2.23 (s, 3H), 1.68 (d, *J* = 11.4 Hz, 2H), 1.57–1.62 (m, 2H), 1.29 (d, *J* = 5.4 Hz, 2H) 0.82 (t, *J* = 7.2 Hz, 3H), 0.68 (d, *J* = 6.6 Hz, 6H). ^13^C NMR (150 MHz, CD_3_OD): δ 171.98, 171.52, 164.21, 158.72, 158.21, 152.23, 149.26, 146.14, 143.47, 141.67, 139.08, 137.76, 133.41, 129.76, 128.31, 125.86, 125.54, 124.67, 124.27, 124.16, 121.26, 120.68, 109.70, 108.59, 101.99, 66.81, 58.22, 41.71, 37.24, 35.20, 30.42, 25.32, 21.50, 18.27, 17.15, 13.58, 11.60. HRMS (ESI) for C_40_H_49_N_4_O_6_ [M + H^+^]: calcd; 681.3652; found, 681.3623.

#### *N*-((4,6-Dimethyl-2-oxo-1,2-dihydropyridin-3-yl)methyl)-5-(ethyl(tetrahydro-2*H*-pyran-4-yl)amino)-3′-(*N*-ethyl-2,4-dihydroxy-5-isopropylbenzamido)-4-methyl-[1,1′-biphenyl]-3-carboxamide (**8**)

The title compound **8** was synthesized in 68% yield from starting material **47** in a manner similar to that described for compound **3**. HPLC purity: 95.02%; mp: 151–153 °C; ^1^H NMR (600 MHz, CD_3_OD): δ 7.46 (t, *J* = 7.8 Hz, 1H), 7.40 (d, *J* = 7.8 Hz, 1H), 7.29 (d, *J* = 7.8 Hz, 1H), 7.16 (d, *J* = 1.8 Hz, 1H), 7.08 (s, 1H), 6.98 (s, 1H), 6.57 (s, 1H), 6.26 (s,1H), 6.10 (s, 1H), 4.47 (s, 2H), 4.00 (s, 2H), 3.91 (d, *J* = 10.8 Hz, 2H), 3.35 (t, *J* = 10.2 Hz, 2H), 3.03–3.06 (m, 2H), 2.97–3.00 (m, 1H), 2.83–2.87 (m, 1H), 2.37 (s, 3H), 2.28 (s, 3H), 2.23 (s, 3H), 1.68 (d, *J* = 11.4 Hz, 2H), 1.56–1.62 (m, 2H), 1.21 (t, *J* = 6.6 Hz, 3H) 0.82 (t, *J* = 7.2 Hz, 3H), 0.68 (d, *J* = 7.2 Hz, 6H). ^13^C NMR (150 MHz, CD_3_OD): δ 171.57, 158.90, 158.14, 152.23, 149.28, 144.42, 143.47, 141.70, 139.07, 137.78, 133.43, 129.75, 129.42, 128.25, 126.88, 125.48, 124.83, 124.76, 124.29, 120.72, 109.69, 108.72, 101.95, 66.82, 58.25, 45.05, 41.72, 35.19, 35.12, 31.63, 30.42, 25.32, 22.30, 21.50, 18.26, 17.14, 13.58, 13.00, 11.63, 11.61. HRMS (ESI) for C_41_H_51_N_4_O_6_ [M + H^+^]: calcd; 695.3809; found, 695.3773.

#### 3′-(2,4-Dihydroxy-5-isopropyl-*N*-propylbenzamido)-*N*-((4,6-dimethyl-2-oxo-1,2-dihydropyridin-3-yl)methyl)-5-(ethyl(tetrahydro-2*H*-pyran-4-yl)amino)-4-methyl-[1,1′-biphenyl]-3-carboxamide (**9**)

The title compound **9** was synthesized in 71% yield from starting material **48** in a manner similar to that described for compound **3.** HPLC purity: 97.52%; mp: 172–175 °C; ^1^H NMR (600 MHz, DMSO-*d*_6_): δ 11.43 (s, 1H), 10.56 (s, 1H), 9.69 (s, 1H), 8.11 (t, *J* = 4.8 Hz, 1H), 7.36–7.40 (m, 2H), 7.26 (s, 1H), 7.16 (d, *J* = 7.2 Hz, 1H), 7.10 (d, *J* = 1.2 Hz, 1H), 7.03 (s, 1H), 6.50 (s,1H), 6.21 (s, 1H), 5.83 (s, 1H), 4.26 (d, *J* = 4.8 Hz, 2H), 3.79 (t, *J* = 7.8 Hz, 4H), 3.22 (t, *J* = 10.8 Hz, 2H), 2.97–3.01 (m, 2H), 2.89–2.94 (m, 1H), 2.74–2.79 (m, 1H), 2.19 (s, 3H), 2.17 (s, 3H), 2.07 (s, 3H), 1.59 (d, *J* = 11.4 Hz, 2H), 1.46–1.53 (m, 4H), 0.83 (t, *J* = 7.2 Hz, 3H), 0.76 (t, *J* = 7.2 Hz, 3H), 0.65 (d, *J* = 7.2 Hz, 6H). ^13^C NMR (150 MHz, DMSO-*d*_6_): δ 170.55, 169.37, 163.45, 157.80, 150.03, 149.28, 144.65, 141.62, 140.09, 137.05, 133.41, 130.16, 128.00, 126.44, 126.05, 125.22, 125.03, 123.65, 122.02, 121.34, 110.13, 107.85, 102.70, 66.73, 65.34, 58.18, 51.56, 41.56, 35.31, 30.69, 25.41, 22.70, 20.84, 19.37, 18.62, 15.60, 14.95, 13.05, 11.64. HRMS (ESI) for C_42_H_53_N_4_O_6_ [M + H^+^]: calcd; 709.3965 found, 709.3956.

#### 3′-(*N*-Benzyl-2,4-dihydroxy-5-isopropylbenzamido)-*N*-((4,6-dimethyl-2-oxo-1,2-dihydropyridin-3-yl)methyl)-5-(ethyl(tetrahydro-2*H*-pyran-4-yl)amino)-4-methyl-[1,1′-biphenyl]-3-carboxamide (**10**)

The title compound **10** was synthesized in 53% yield from starting material **49** in a manner similar to that described for compound **6**. HPLC purity: 95.26%; mp: 146–148 °C; ^1^H NMR (600 MHz, CD_3_OD): δ 7.36 (t, *J* = 7.2 Hz, 1H), 7.32 (t, *J* = 9 Hz, 3H), 7.25 (t, *J* = 7.2 Hz, 2H), 7.19 (t, *J* = 7.2 Hz, 2H), 7.05 (d, *J* = 0.6 Hz, 1H), 6.91 (d, *J* = 9 Hz, 2H), 6.64 (s, 1H), 6.27 (s,1H), 6.10 (s, 1H), 5.16 (s, 2H), 4.47 (s, 2H), 3.90 (d, *J* = 11.4 Hz, 2H), 3.34 (t, *J* = 10.8 Hz, 2H), 3.01–3.04 (m, 2H), 2.94–2.98 (m, 1H), 2.83–2.88 (m, 1H), 2.37 (s, 3H), 2.26 (s, 3H), 2.23 (s, 3H), 1.65 (d, *J* = 12 Hz, 2H), 1.54–1.61 (m, 2H), 0.80 (t, *J* = 7.2 Hz, 3H), 0.68 (d, *J* = 6.6 Hz, 6H). ^13^C NMR (150 MHz, CD_3_OD): δ 171.79, 171.50, 164.22, 158.78, 158.25, 152.23, 149.20, 144.41, 143.47, 141.41, 139.04, 137.70, 137.43, 133.34, 129.54, 128.13, 128.10, 128.04, 127.05, 126.85, 125.57, 124.84, 124.81, 124.13, 121.28, 120.65, 109.71, 108.80, 102.00, 66.81, 58.22, 54.64, 52.91, 41.67, 35.17, 30.40, 28.11, 25.36, 21.50, 18.29, 17.15, 13.56, 11.60. HRMS (ESI) for C_46_H_53_N_4_O_6_ [M + H^+^]: calcd; 757.3965 found, 757.3998.

### EZH2 Inhibition Assay

The EZH2 inhibitory ability of the synthesized adduct was assessed by Reaction Biology Corporation Malvern, PA. (http://www.reactionbiology.com).

### *In Vitro* Hsp90 Assay

The HSP90 inhibitory activity of the synthesized adduct was assessed by Reaction Biology Corporation Malvern, PA. (http://www.reactionbiology.com).

### Cell culture

Pt3 GBM cells were isolated from a male GBM patient as described in our previous study.^54,58^ Temozolomide (TMZ)-resistant phenotype of Pt3R cells was validated previously.^53−59^ Pt3 cells were cultured in DMEM-supplemented with 10% fetal bovine serum, 100 μg/mL streptomycin, and 100 μg/mL penicillin G. Pt3R cells were acquired from culturing in the presence of 100 μM TMZ (MilliporeSigma Corporate, St. Louis, MO) for, at least, 6 months, and the TMZ-resistant characteristics were confirmed in the previous studies.^54,58,59^

### Cell Viability CCK8 Assay

The CCK8 reagent was purchased from TargetMOI (Wellesley Hills, MA) and used according to the manufacturer’s instruction.

### Molecular Docking

Structure-based molecular docking was performed to screen compound **7** based on the binding energy. The docking process was carried out between protein and compounds with a population size of 1500, generations of 150, and solutions of 10 through iGEMDOCK version 2.1. Protein structures of HSP90 and EZH2 were obtained from Protein Data Bank (8AGI and 4W2R).

### RNA-seq and Proteomic Assay

RNA was isolated using the RNA extracting kit (Zymo Research, Irvine, CA) and subjected to RNA-seq serviced by BIOTOOLS Co., Ltd. (New Taipei City, Taiwan). Cell pellets were collected and subjected to proteomic analysis serviced by BIOTOOLS Co., Ltd. The comparison in gene expression between Pt3 and Pt3R was performed using RNA seq and described previously.^60^

### ROS Assay and MitoSOX Assay

Dihydrorhodamine 123 (DHR; Thermo Fisher Scientific, Waltham, MA) was used to label cellular ROS detected by the flow cytometry,^54^ and the MitoSOX reagent (Thermo Fisher Scientific) was used to label mitochondrial ROS.^55^

### Western Blotting

The detailed procedure was described in our previous study.^54,55,58,59^ Primary antibodies, anti-CENPA, anti-CENPE, anti-CENPF, and anti-CENPI antibodies, were purchased from abcam (Cambridge, U.K.); anti-CDK1 and anticyclin B1 antibodies were purchased from Santa Cruz Biotechnology, Inc. (Dallas, TX); and anti-EZH2 and anti-H3K27^me3^ antibodies were purchased from GeneTex International Corporation (HsinChu, Taiwan), anti-Caspase-3 was purchased from GeneTex International Corporation; anti-cleaved Caspase-3 was purchased from Cell Signaling Technology (Danvers, Massachusetts).

### Bioinformatic Analysis

Gepia and GlioVis websites were employed to generate expression and survival plots.

### Animal Experiment

Animal experiments were approved by the institutional animal care and use committee of Taipei Medical University (IACUC: LAC2022-0288). Male *NOD.CB17-Prkdc*^*scid*^*/NCrCrl* mice (8-week-old) were purchased from BioLASCO Taiwan Co., Ltd. (Taipei, Taiwan). Pt3R cells (1 × 10^6^) in 50 μL of DMEM were injected into the back of mice. Experimental mice were grouped as follows (Figure 11): DMSO (*n* = 5), C7–10 mg/kg (*n* = 4), C7–20 mg/kg (*n* = 6), TAZ-10 mg/kg (*n* = 6), TAZ-20 mg/kg (*n* = 8), STA-5+TAZ-5 mg/kg (*n* = 7), STA-10+TAZ-10 mg/kg (*n* = 7) and STA-15+TAZ-15 mg/kg (*n* = 7). After 10 days, the tumor was touchable. Mice were injected intraperitoneally with DMSO (the control group) or compounds twice per week.

### Statistical Analysis

Experiments were performed 3 times independently, and data were expressed as mean ± s.e.m. *P* value <0.05 was considered as the significant difference.

## Supporting Information Available

Characterization data of compounds and HPLC purity data for compounds (PDF)
Molecular formula strings (CSV)
EZH2(4w2r)_Compound **7**(CO7) (PDB)
EZH2(4w2r)_Tazemetostat(TAZ) (PDB)
HSP90(8agi)_Compound **7**(CO7) (PDB)
HSP90(8agi)_Ganetespib(TUH) (PDB)

## Supplementary Material

jm3c02053_si_002.csv

jm3c02053_si_003.pdb

jm3c02053_si_004.pdb

jm3c02053_si_005.pdb

jm3c02053_si_006.pdb
