# Supplementary material for: First-in-Class Dual EZH2-HSP90 Inhibitor Eliciting Striking Antiglioblastoma Activity In Vitro and In Vivo
Source: J Med Chem. 2024 Jan 29;67(4):2963–85. doi: 10.1021/acs.jmedchem.3c02053 (PMC10895674; doi:10.1021/acs.jmedchem.3c02053)
Supplement: Supplementary file 1 — jm3c02053_si_001.pdf [file jm3c02053_si_001.pdf]

Supporting Information

First-in-class dual EZH2-HSP90 inhibitor eliciting striking anti-glioblastoma activity *in vitro* and *in vivo*

Sachin Sharma[a#], Shao-An Wang[b#], Wen-Bin Yang[c], Hong-Yi Lin[d], Mei-Jung Lai[e], Hsien-Chung Chen[c, f, g], Tzu-Yuan Kao[a,g], Feng-Lin Hsu[b], Kunal Nepali [a, e, j]*, Tsung-I Hsu[c, e, g, h, i]* Jing-Ping Liou [a, e, i, j]*

[#]Contributed equally to this work.

[a] School of Pharmacy, College of Pharmacy, Taipei Medical University, Taipei 110, Taiwan.
[b] School of Respiratory Therapy, College of Medicine, Taipei Medical University, Taipei 110, Taiwan.
[c] TMU Research Center of Neuroscience, Taipei Medical University, Taipei 110, Taiwan
[d] Graduate Institute of Medical Sciences, College of Medicine, Taipei Medical University, Taipei 110, Taiwan
[e] TMU Research Center for Drug Discovery, Taipei Medical University, Taipei 110, Taiwan.
[f] Department of Neurosurgery, Shuang Ho Hospital, Taipei Medical University, Taipei 110, Taiwan
[g] Ph.D. Program in Medical Neuroscience, College of Medical Science and Technology, Taipei Medical University and National Health Research Institutes, Taipei 110, Taiwan
[h] International Master Program in Medical Neuroscience, College of Medical Science and Technology, Taipei Medical University, Taipei 110, Taiwan
[i] TMU Research Center of Cancer Translational Medicine, Taipei 110, Taiwan
[j] Ph.D. Program in Drug Discovery and Development Industry, College of Pharmacy, Taipei Medical University, Taipei 110, Taiwan.

Corresponding authors

Kunal Nepali - School of Pharmacy, College of Pharmacy, Taipei Medical University, Taipei, Taiwan.
TMU Research Center for Drug Discovery, Taipei Medical University, Taipei 110, Taiwan.
Ph.D. Program in Drug Discovery and Development Industry, College of Pharmacy, Taipei Medical University, Taipei 110, Taiwan.
Email – nepali@tmu.edu.tw

Tsung-I Hsu - Ph.D. Program in Medical Neuroscience, College of Medical Science and Technology, Taipei Medical University and National Health Research Institutes, Taipei 110, Taiwan
TMU Research Center of Neuroscience, Taipei Medical University, Taipei 110, Taiwan
International Master Program in Medical Neuroscience, College of Medical Science and Technology, Taipei Medical University, Taipei 110, Taiwan
TMU Research Center of Cancer Translational Medicine, Taipei 110, Taiwan
E-mail - dabiemhsu@tmu.edu.tw

Jing Ping Liou - School of Pharmacy, College of Pharmacy, Taipei Medical University, Taipei 110, Taiwan
TMU Research Center for Drug Discovery, Taipei Medical University, Taipei 110, Taiwan.
Ph.D. Program in Drug Discovery and Development Industry, College of Pharmacy, Taipei Medical University, Taipei 110, Taiwan.
E-mail - jpl@tmu.edu.tw

Contents:

1. **^{1}H NMR for compounds 1-10……………………………………………………………S2-S6**
2. **^{13}C NMR for compounds 1-10…………………………………………………………..S7-S11**
3. **HPLC purity data for compounds 1-10……………………………………………….S12-S21**
4. **HRMS for compounds 1-10…………………………………………………………S22-S26**
5. **BBB permeability of compound 7………………………………………………………….S27**
6. **Pharmacokinetic data of compound 7……………………………………….……….S28**

^{1}H NMR of compound 1

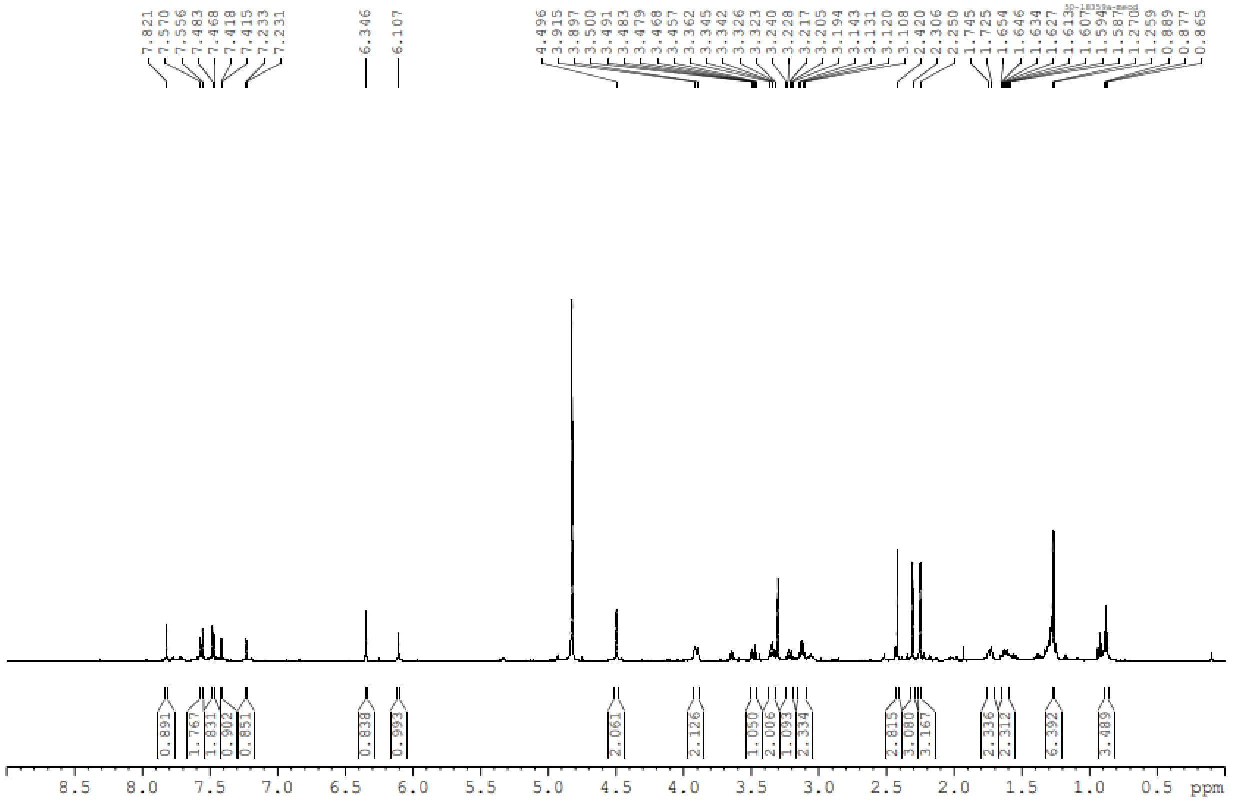

^{1}H NMR of compound 2

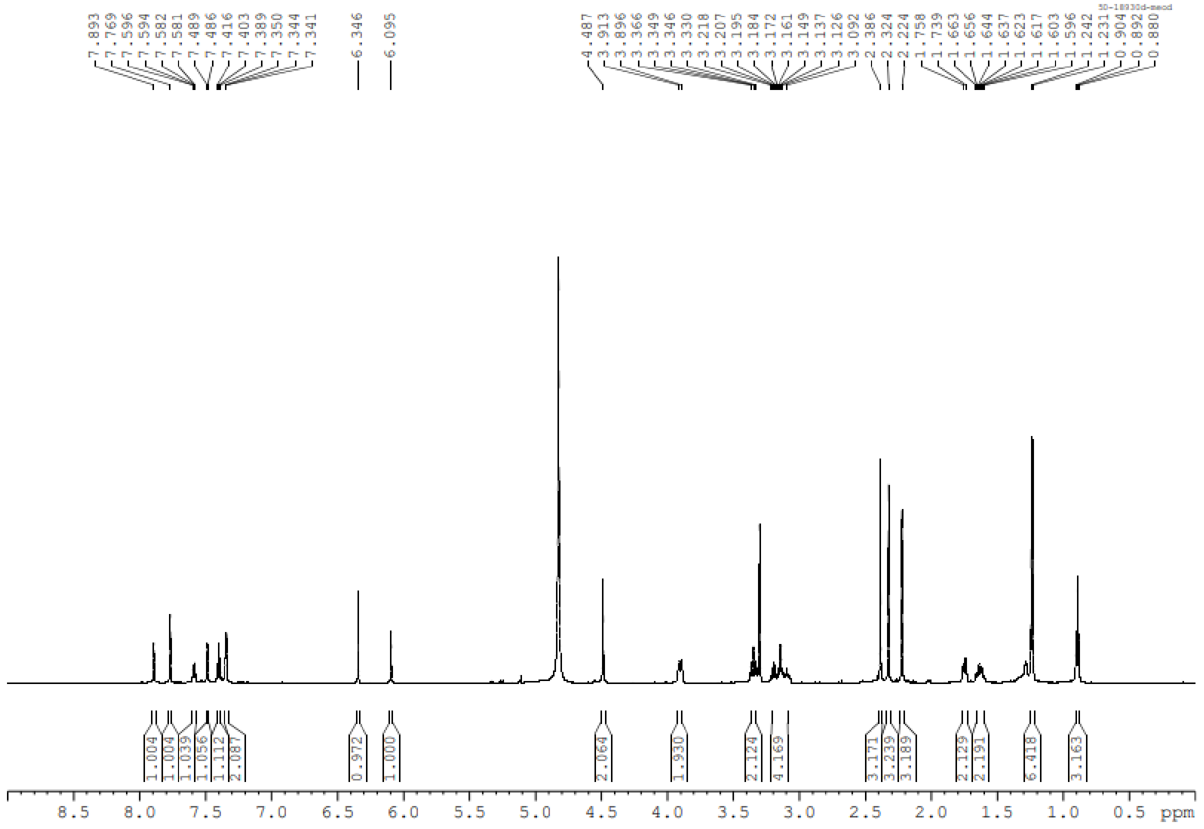

^{1}H NMR of compound 3

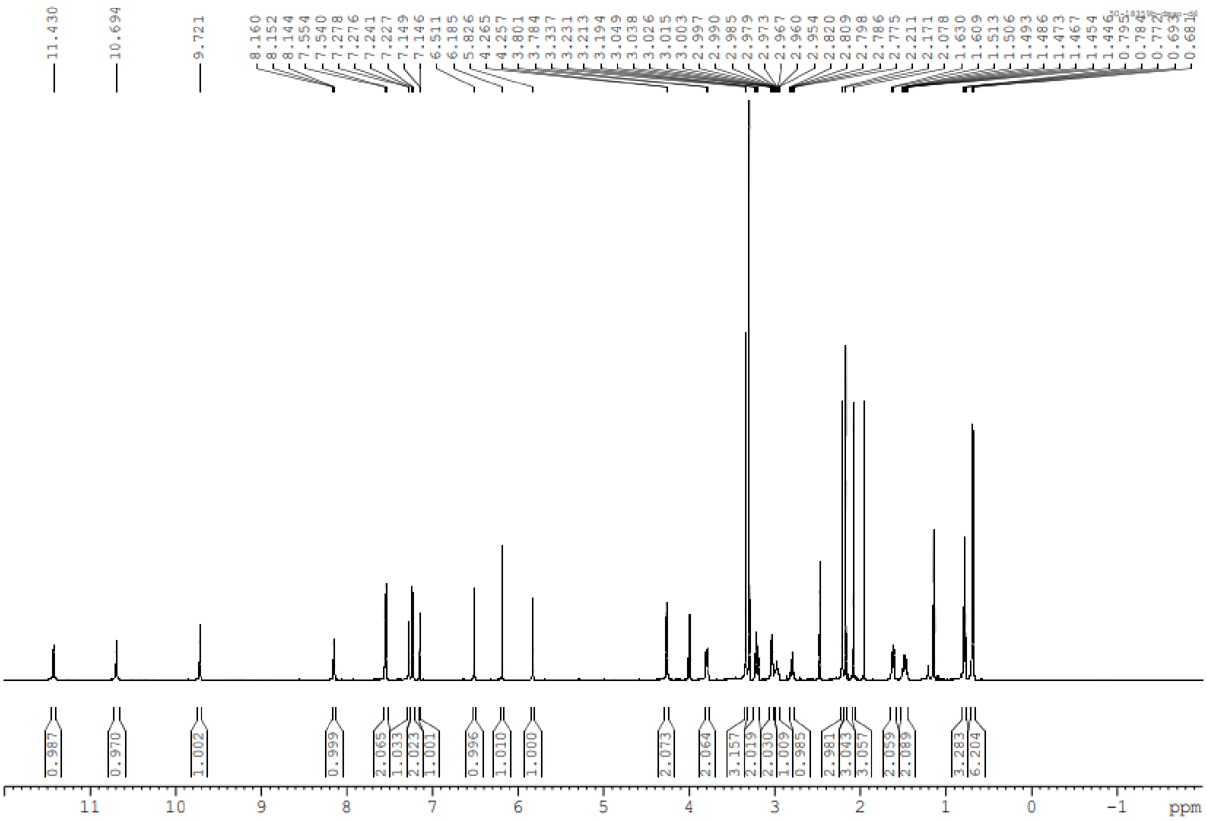

^{1}H NMR of compound 4

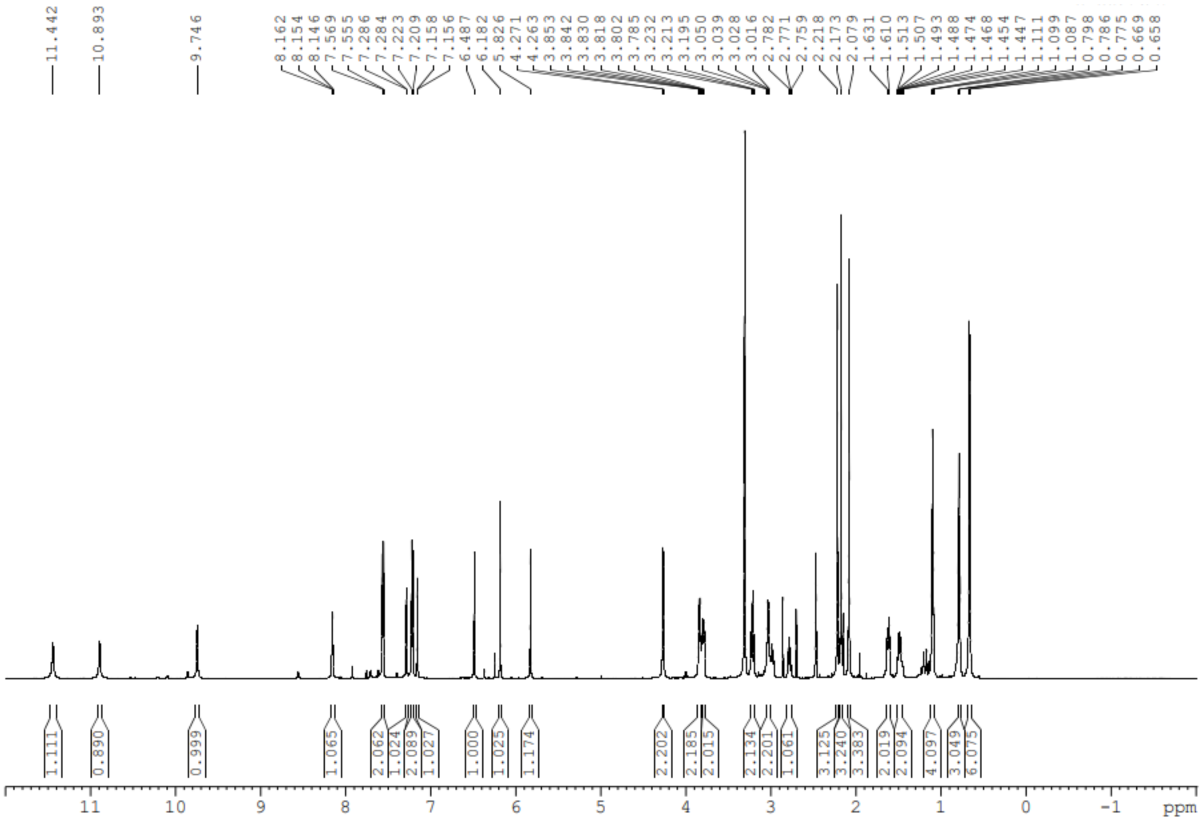

^{1}H NMR of compound 5

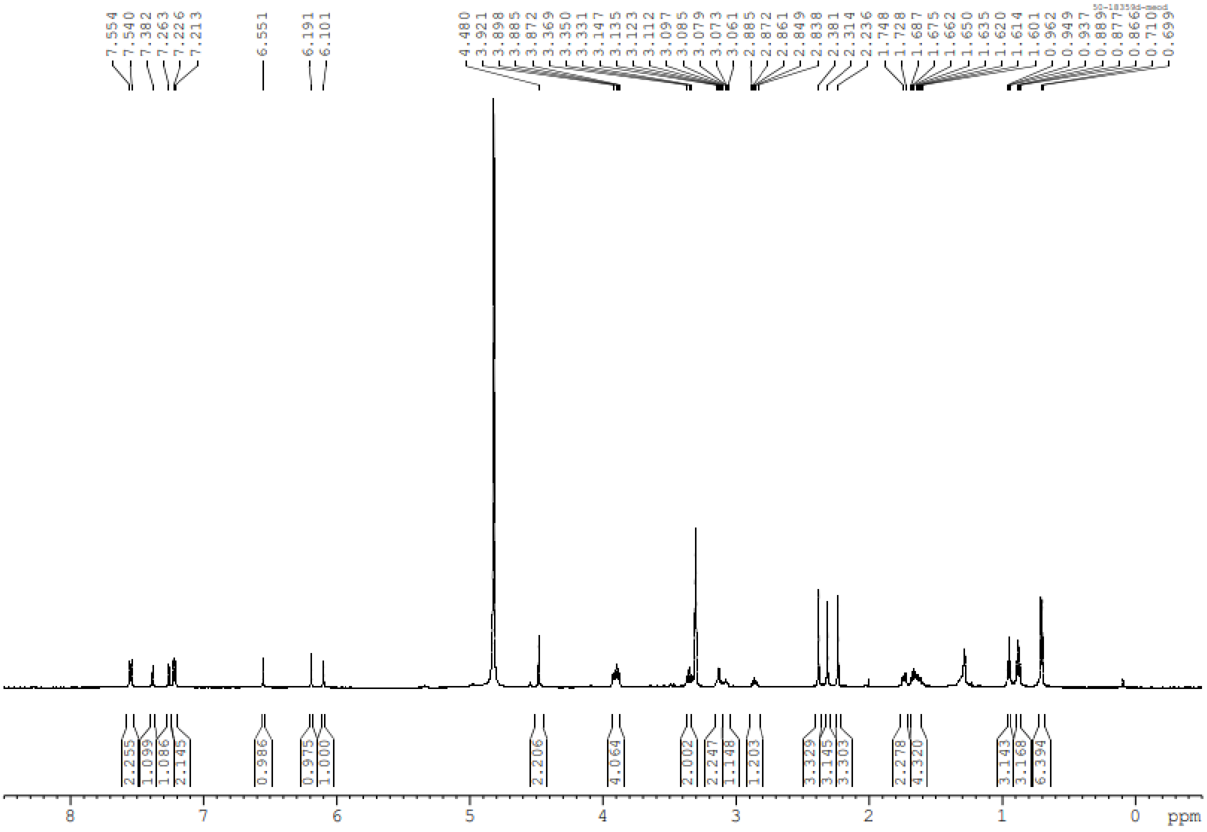

^{1}H NMR of compound 6

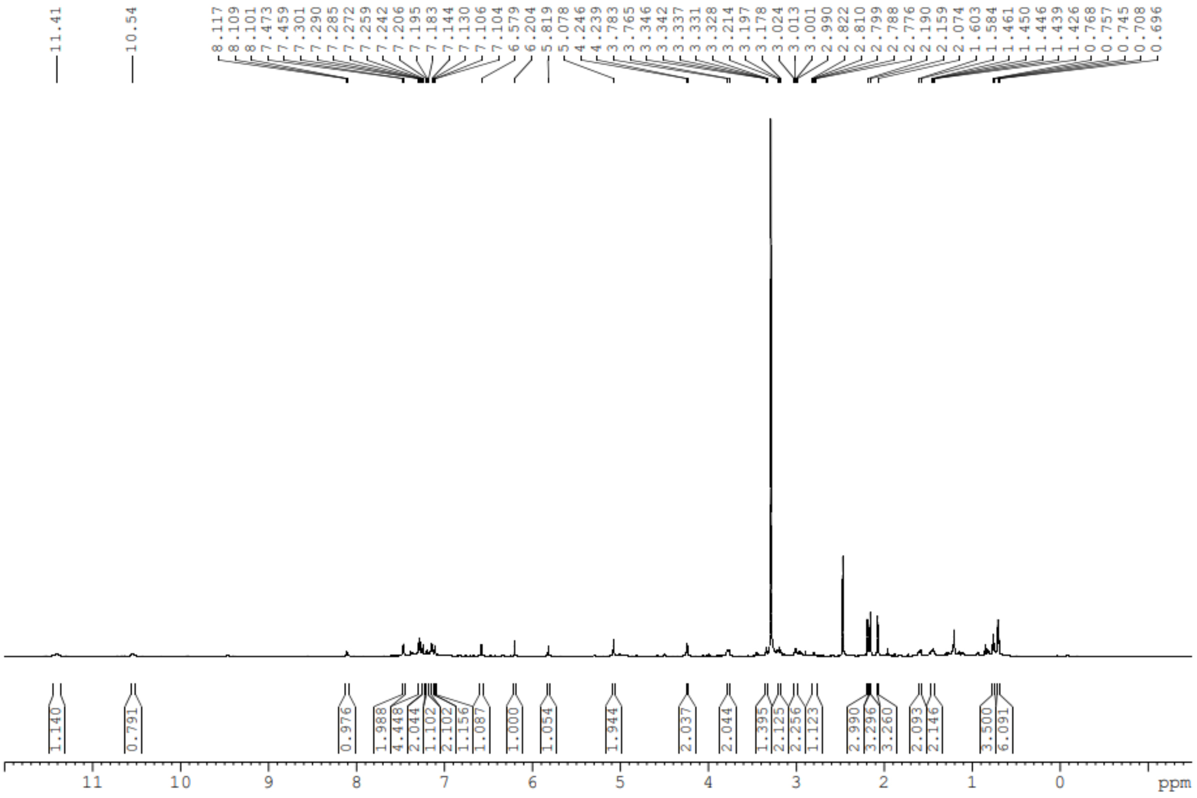

^{1}H NMR of compound 7

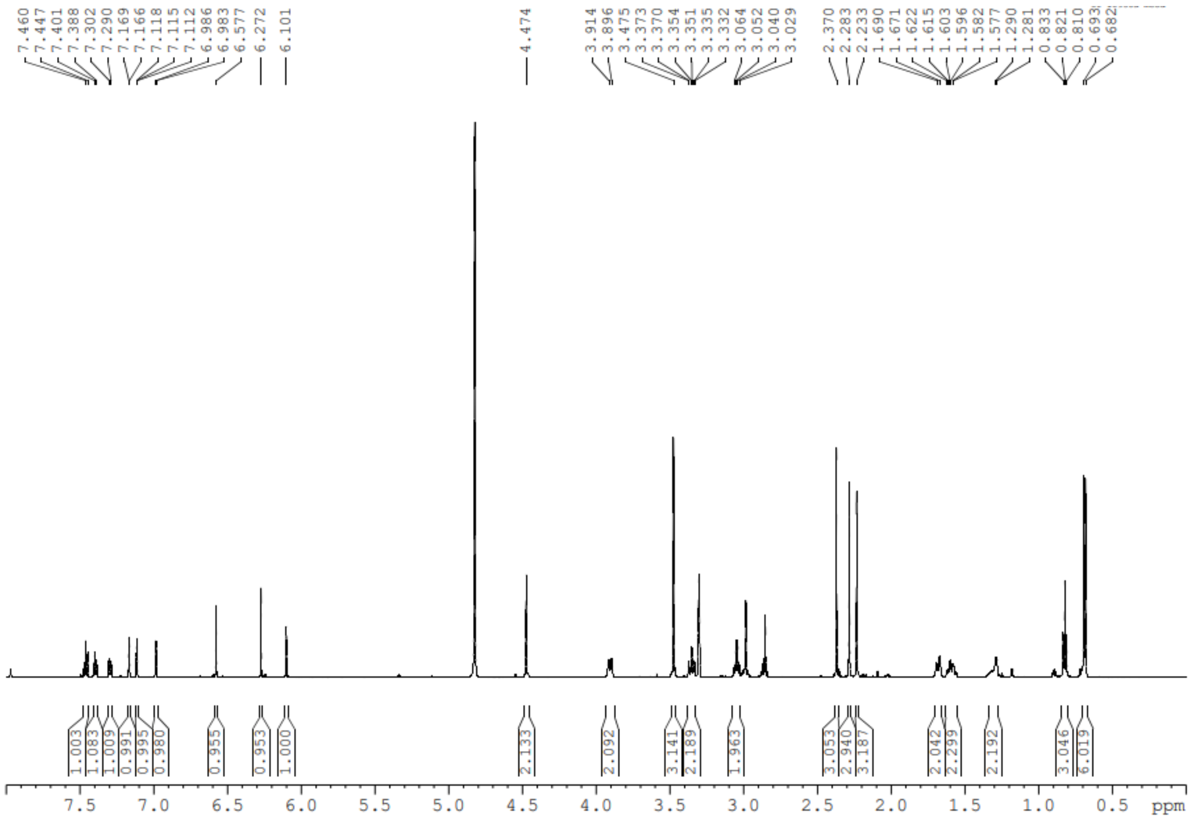

^{1}H NMR of compound 8

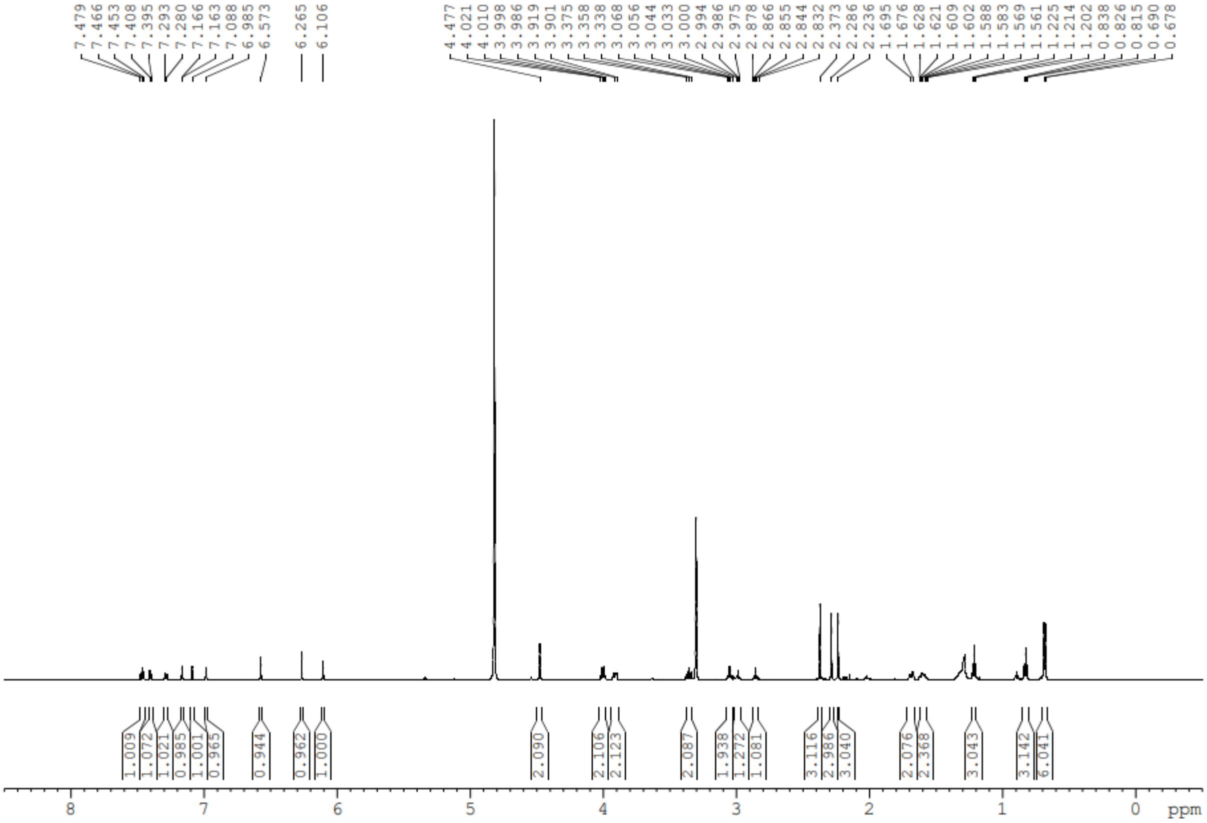

^{1}H NMR of compound 9

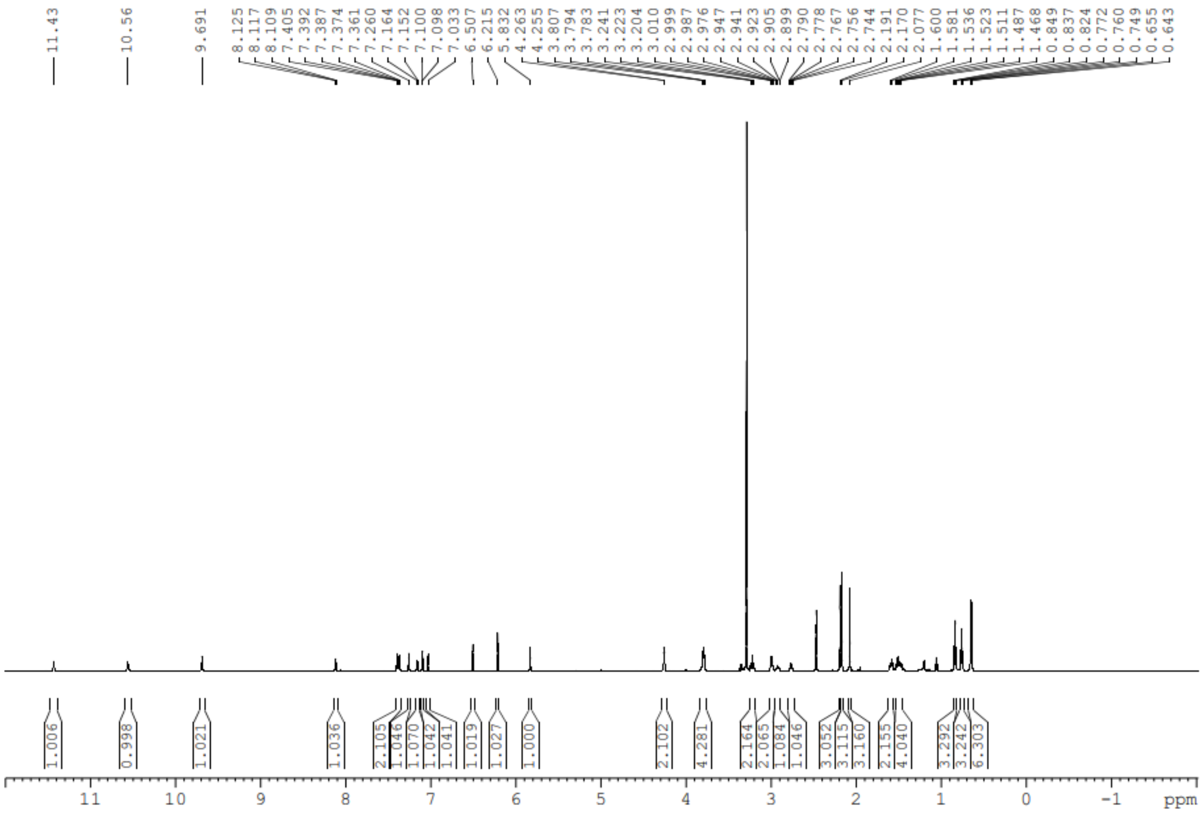

^{1}H NMR of compound 10

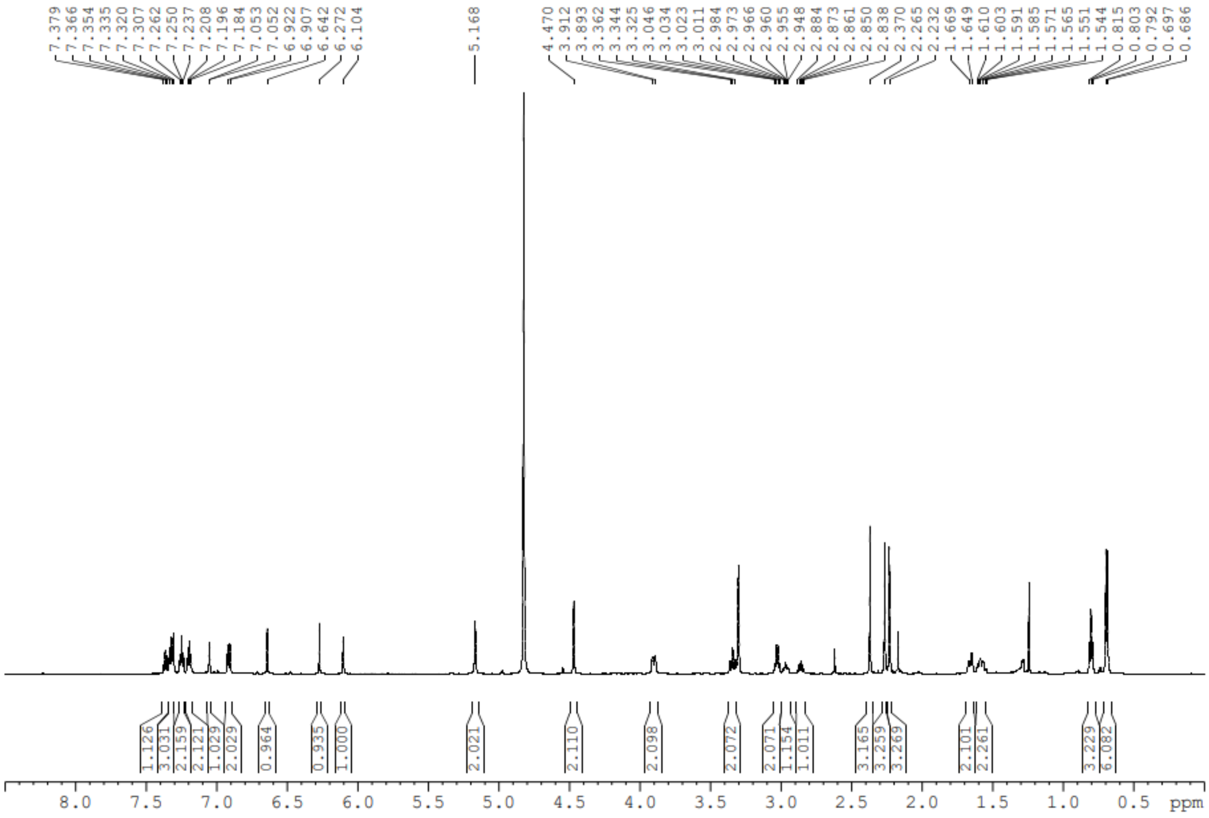

^{13}C NMR of compound 1

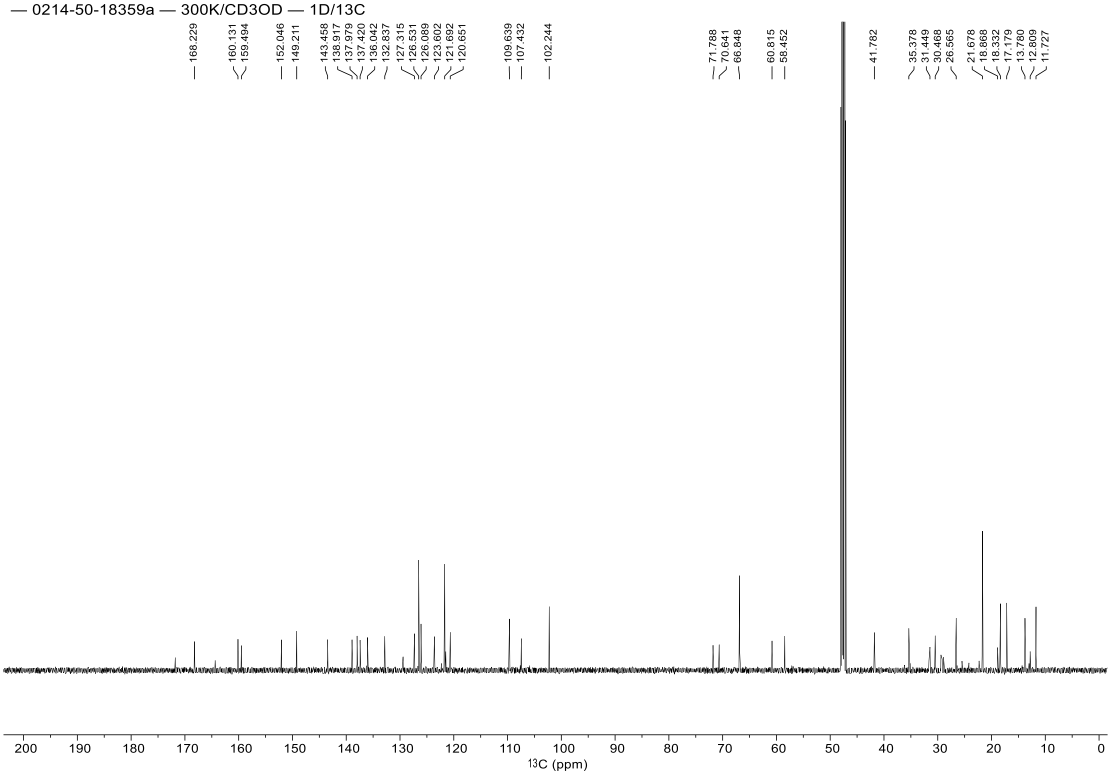

^{13}C NMR of compound 2

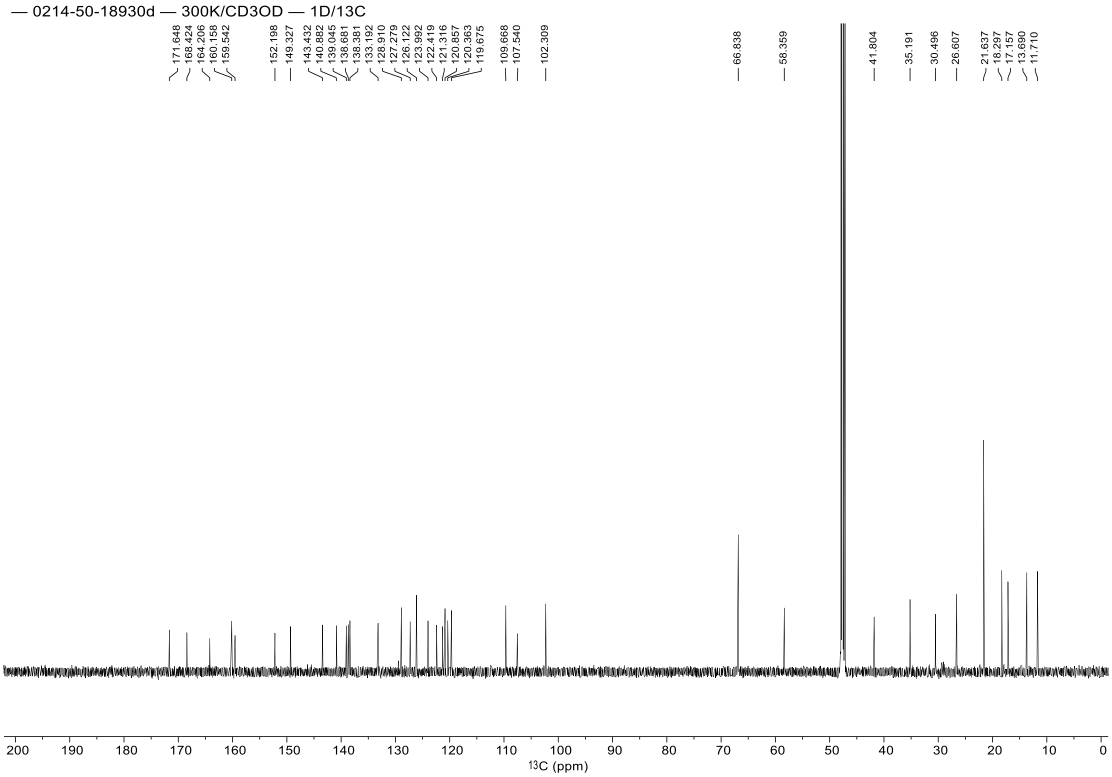

^{13}C NMR of compound 3

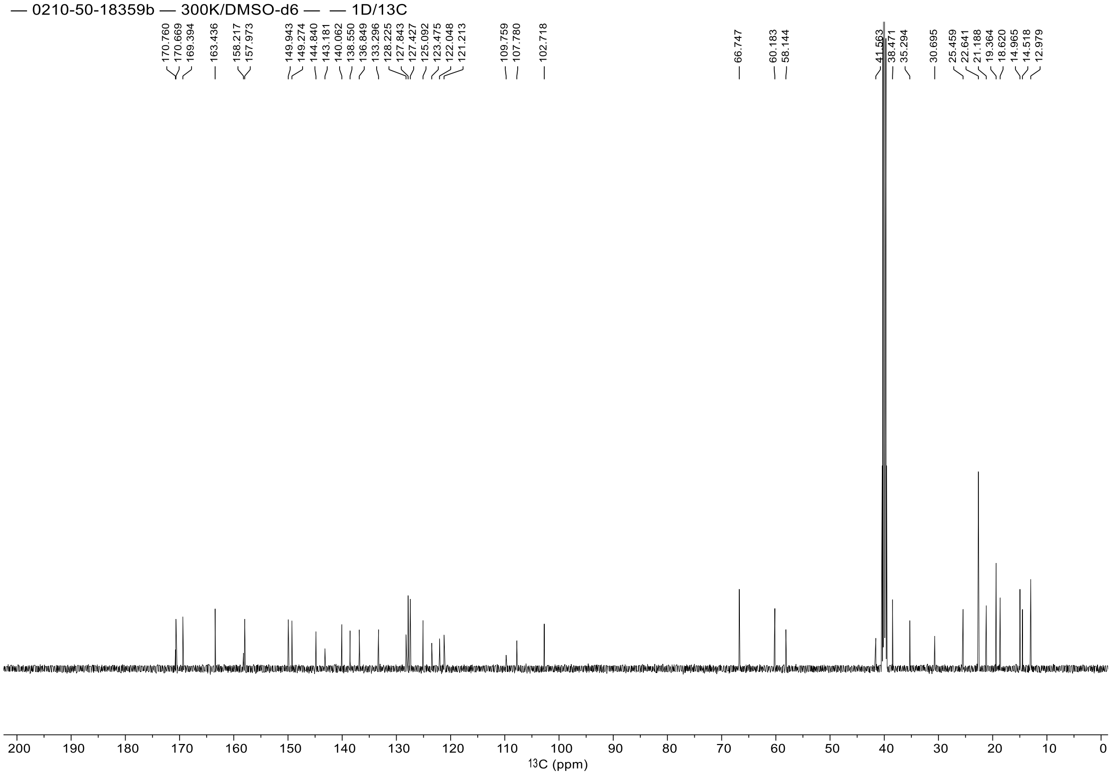

^{13}C NMR of compound 4

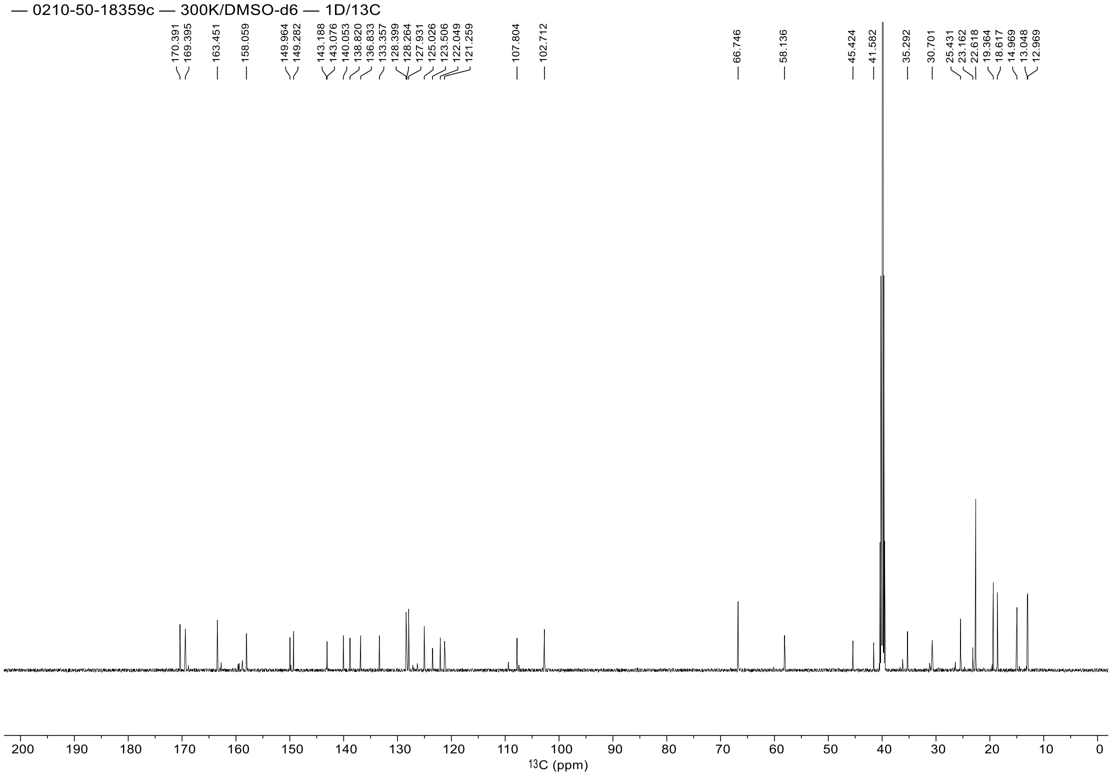

^{13}C NMR of compound 5

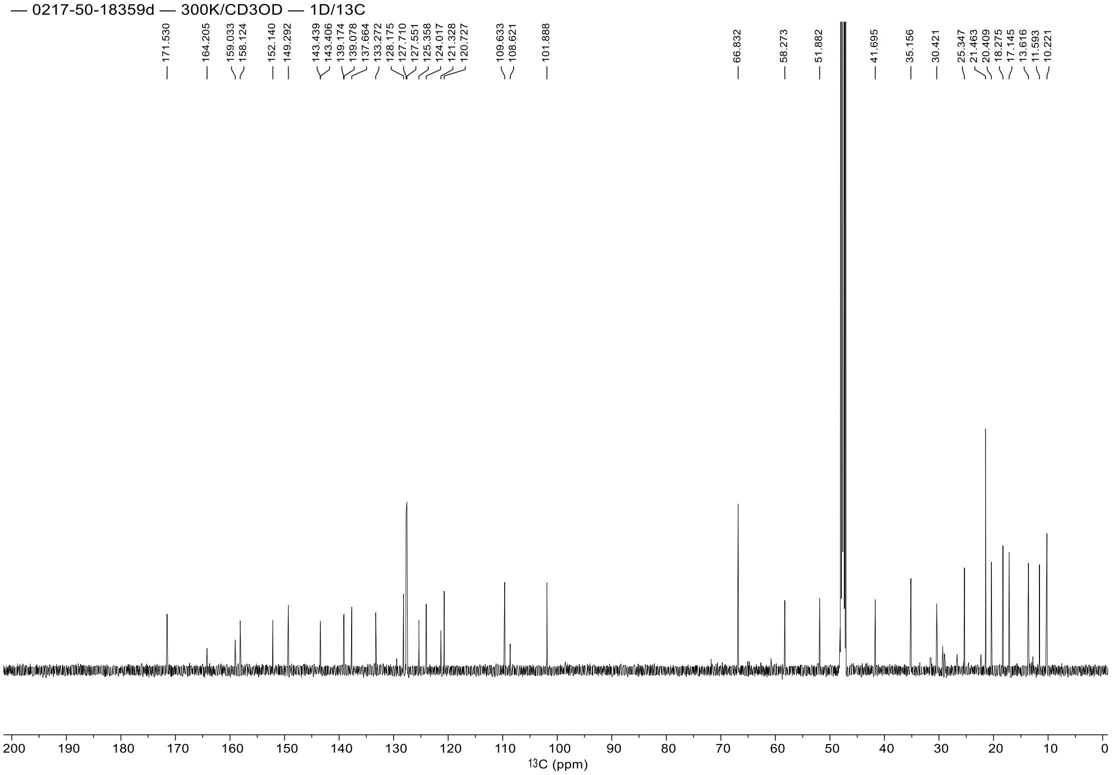

^{13}C NMR of compound 6

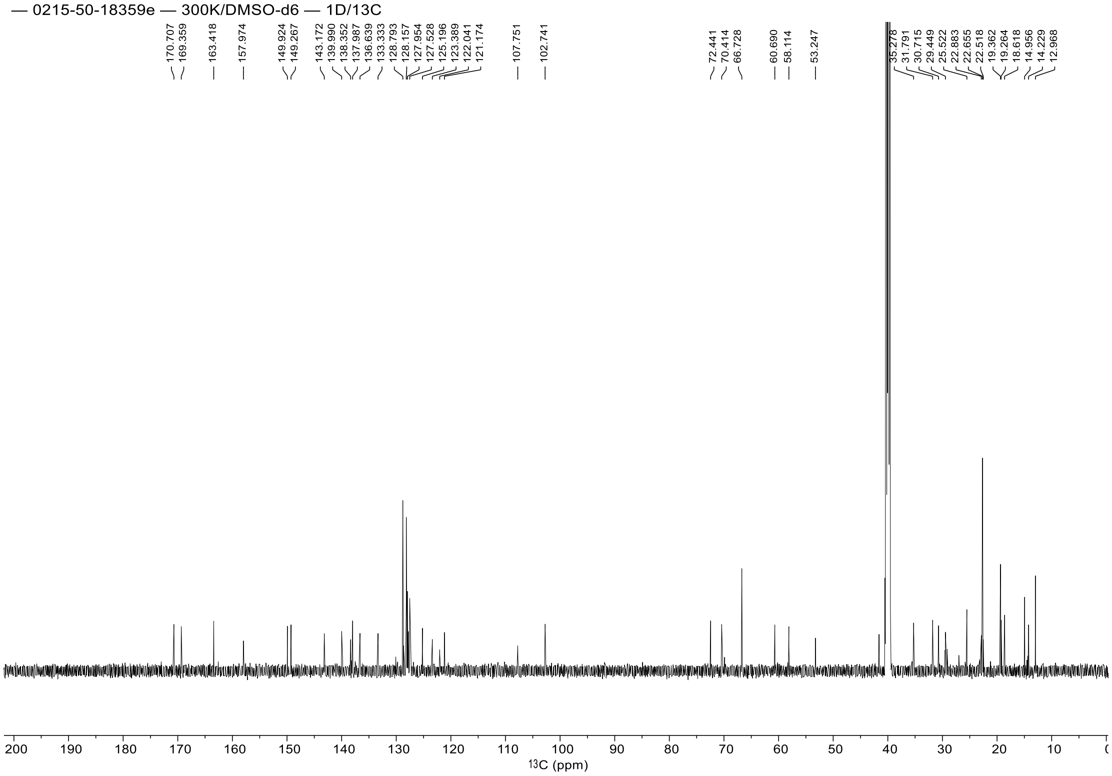

^{13}C NMR of compound 7

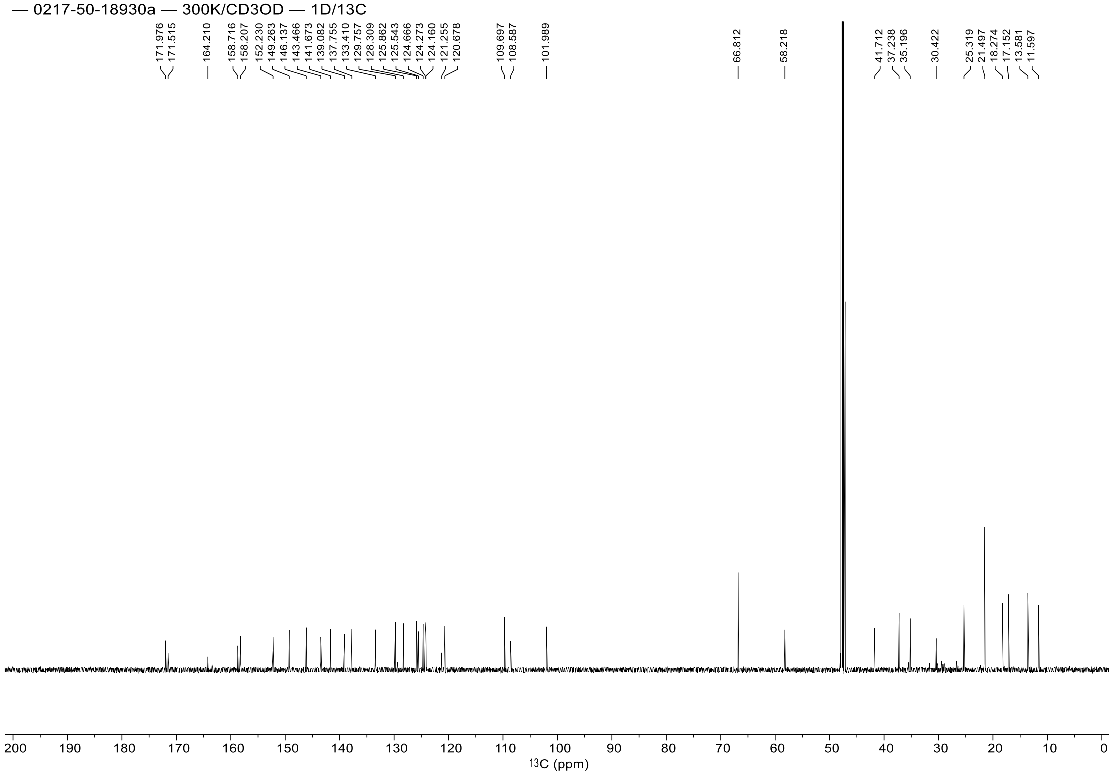

^{13}C NMR OF compound 8

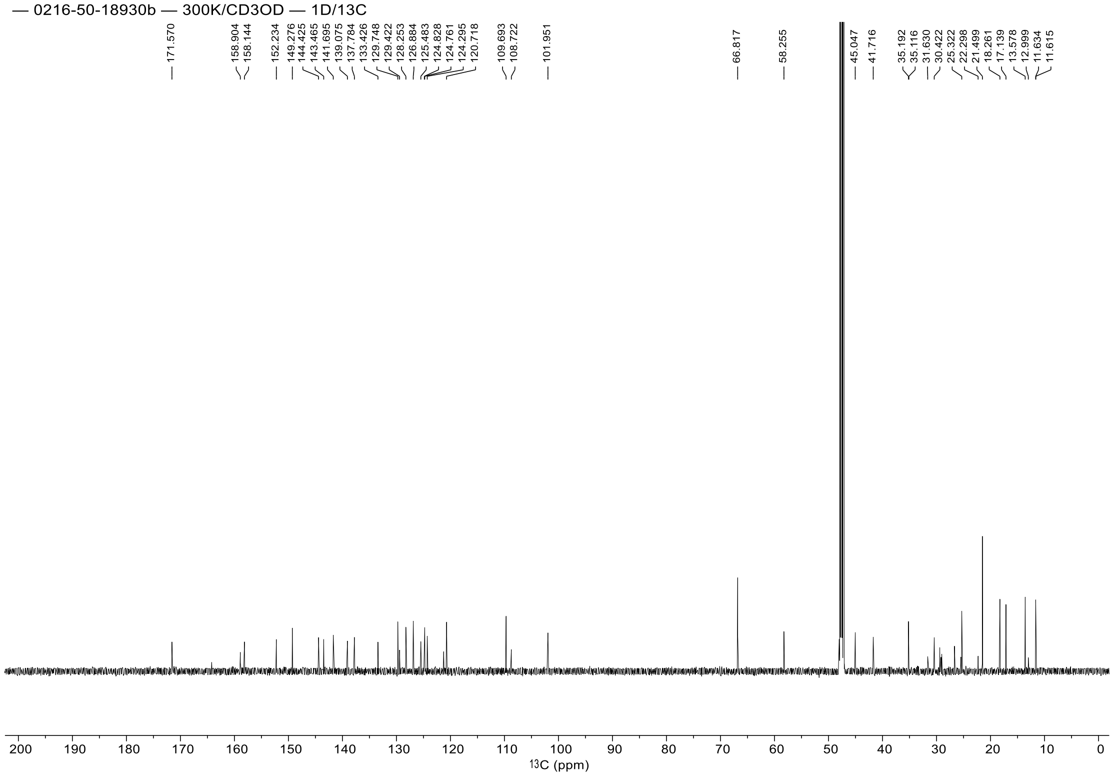

^{13}C NMR of compound 9

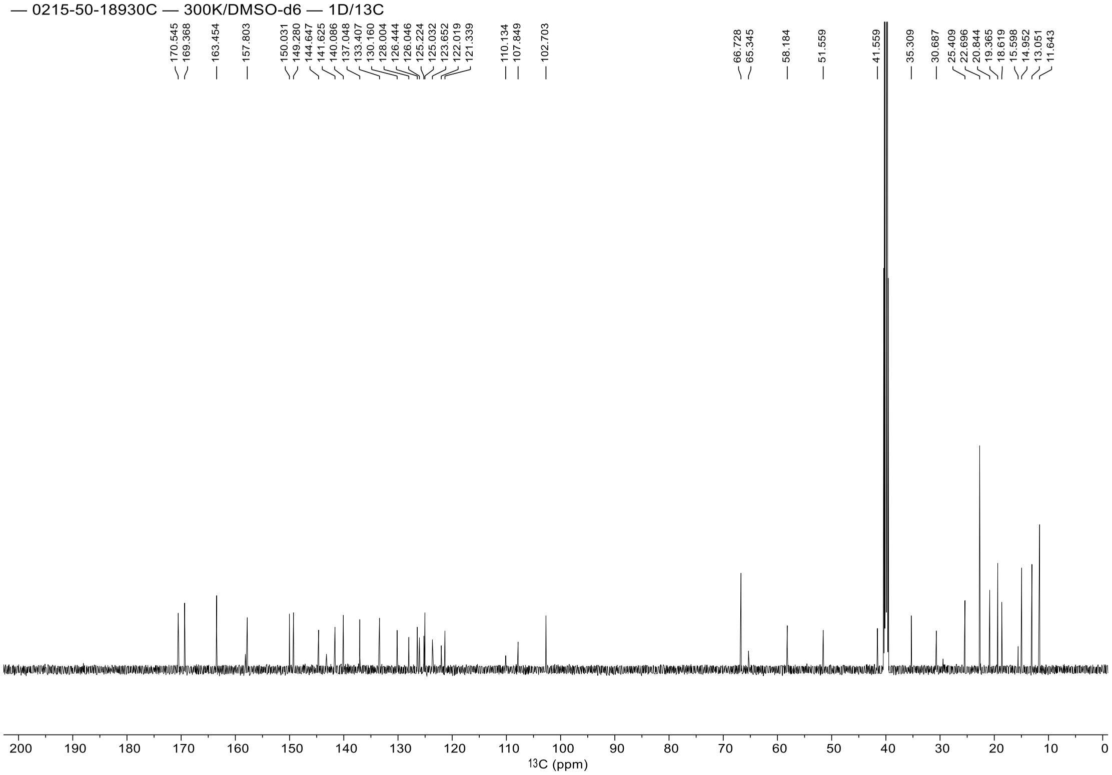

^{13}C NMR OF compound 10

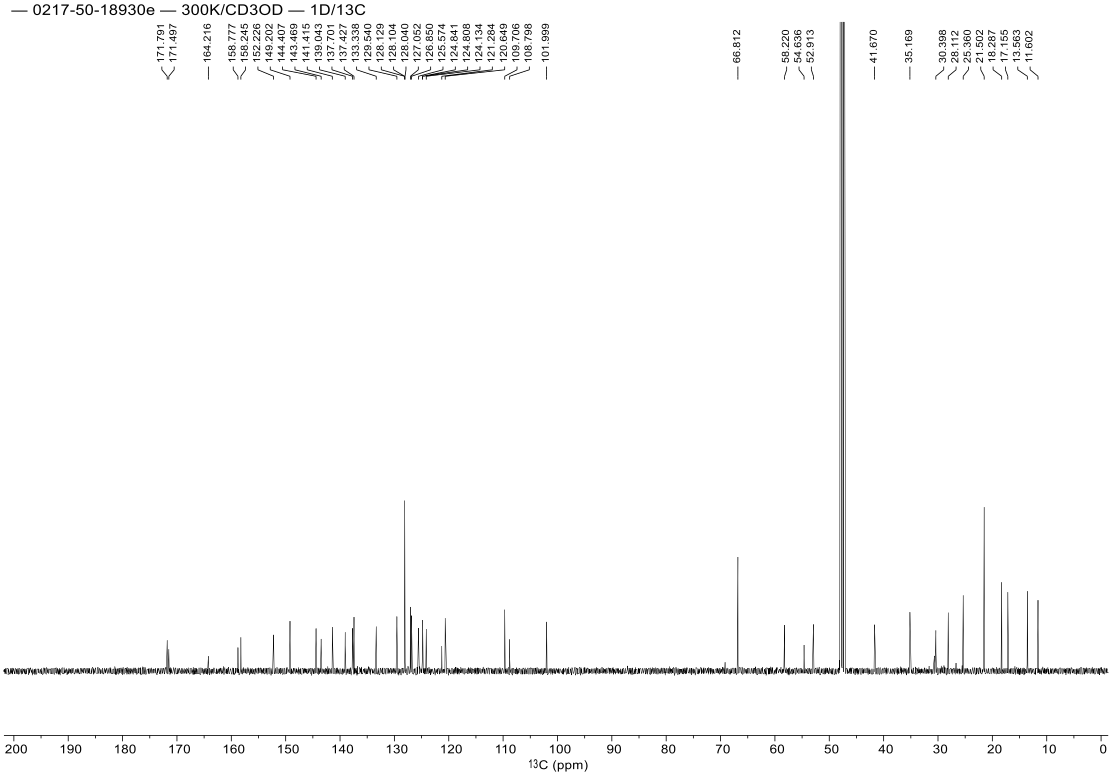

HPLC purity data of compound 1

Analysis Report

<Sample Information>

Sample Name : 50-18359A
Sample ID :
Data Filename : 2023224_50-18359A_003.lcd
Method Filename : NHRI purity test.lcm
Batch Filename : 20230224_hplc.lcb
Vial # : 3-3
Sample Type : Unknown
Injection Volume : 20 uL
Date Acquired : 2023/2/24 07:38:14
Acquired by : System Administrator
Date Processed : 2023/3/1 02:13:37
Processed by : System Administrator

<Chromatogram>

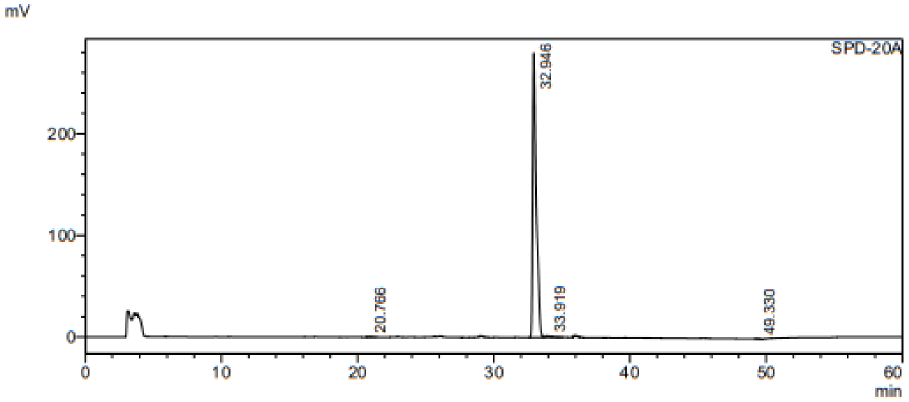

Peak Table

SPD-20A

Peak#	Ret. Time	Area	Height	Conc.	Area%
1	20.766	6284	375	0.121	0.121
2	32.946	5142701	279556	98.734	98.734
3	33.919	52865	1389	1.015	1.015
4	49.330	6804	362	0.131	0.131
Total		5208653	281682		100.000

D:\50\HPLC data 50\2023224_50-18359A_003.lcd

HPLC purity data of compound 2

Analysis Report

<Sample Information>

Sample Name	: 50-18390D		
Sample ID	:		
Data Filename	: 2023222_50-18390D_012.lcd		
Method Filename	: NHRI purity test.lcm		
Batch Filename	: 20230222_hplc.lcb		
Vial #	: 3-12	Sample Type	: Unknown
Injection Volume	: 20 uL		
Date Acquired	: 2023/2/23 04:33:46	Acquired by	: System Administrator
Date Processed	: 2023/2/23 11:28:07	Processed by	: System Administrator

<Chromatogram>

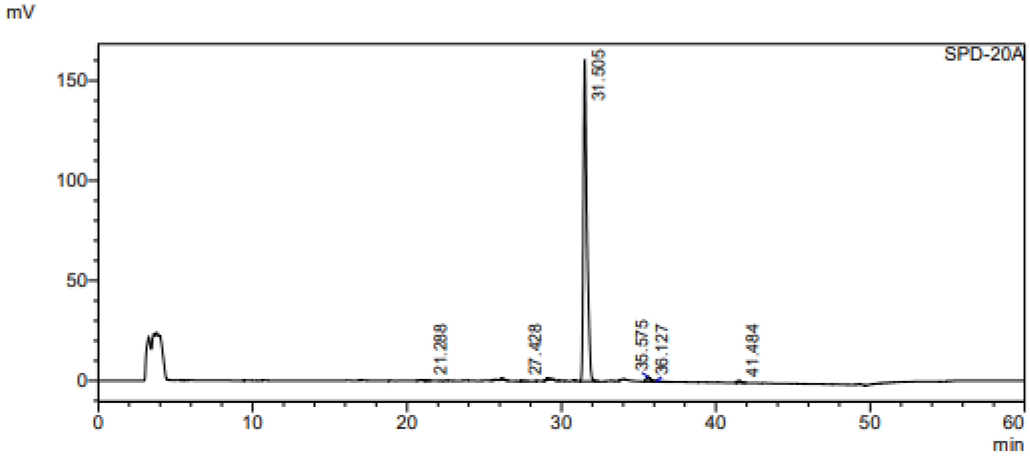

Peak Table

SPD-20A

Peak#	Ret. Time	Area	Height	Conc.	Area%
1	21.288	1670	189	0.064	0.064
2	27.428	2688	259	0.103	0.103
3	31.505	2531788	160303	97.258	97.258
4	35.575	42126	2536	1.618	1.618
5	36.127	10629	522	0.408	0.408
6	41.484	14270	914	0.548	0.548
Total		2603172	164724		100.000

HPLC purity data of compound 3

Analysis Report

<Sample Information>

Sample Name	: 50-18359B		
Sample ID	:		
Data Filename	: 2023222_50-18359B_004.lcd		
Method Filename	: NHRI purity test.lcm		
Batch Filename	: 20230222_hplc.lcb		
Vial #	: 3-4	Sample Type	: Unknown
Injection Volume	: 20 uL		
Date Acquired	: 2023/2/22 08:30:36	Acquired by	: System Administrator
Date Processed	: 2023/2/23 11:33:43	Processed by	: System Administrator

<Chromatogram>

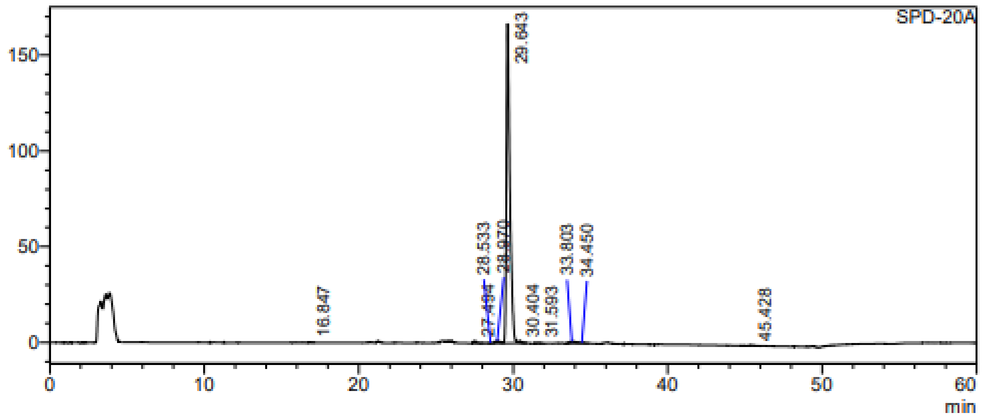

Peak Table

SPD-20A

Peak#	Ret. Time	Area	Height	Conc.	Area%
1	16.847	2668	254	0.098	0.098
2	27.494	15509	1032	0.570	0.570
3	28.533	2881	227	0.106	0.106
4	28.970	26420	1233	0.972	0.972
5	29.643	2614842	166793	96.166	96.166
6	30.404	7178	652	0.264	0.264
7	31.593	5711	341	0.210	0.210
8	33.803	32793	1037	1.206	1.206
9	34.450	7285	452	0.268	0.268
10	45.428	3809	250	0.140	0.140
Total		2719097	172271		100.000

HPLC purity data of compound 4

Analysis Report

<Sample Information>

Sample Name	: 50-18359C		
Sample ID	:		
Data Filename	: 2023224_50-18359C_004.lcd		
Method Filename	: NHRI purity test.lcm		
Batch Filename	: 20230224_hplc.lcb		
Vial #	: 3-4	Sample Type	: Unknown
Injection Volume	: 20 uL		
Date Acquired	: 2023/2/24 08:38:36	Acquired by	: System Administrator
Date Processed	: 2023/2/24 09:38:39	Processed by	: System Administrator

<Chromatogram>

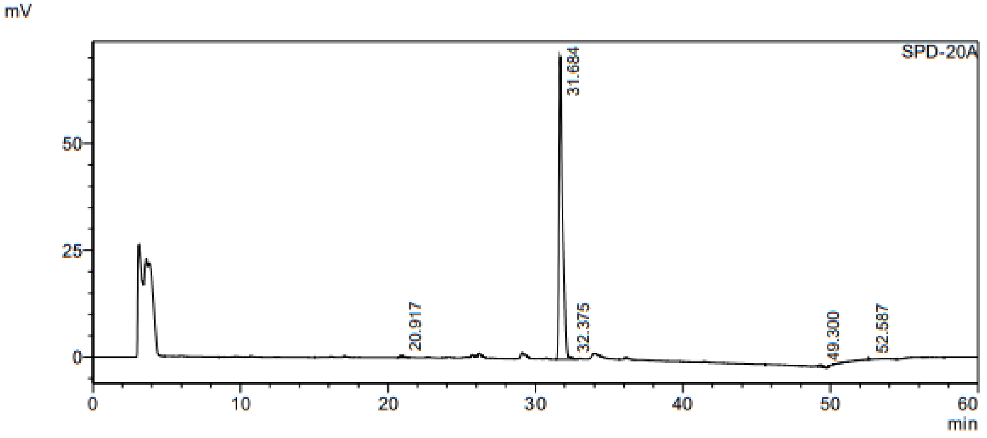

Peak Table

SPD-20A

Peak#	Ret. Time	Area	Height	Conc.	Area%
1	20.917	9206	440	0.752	0.752
2	31.684	1204127	70692	98.385	98.385
3	32.375	1072	133	0.088	0.088
4	49.300	8392	405	0.686	0.686
5	52.587	1097	455	0.090	0.090
Total		1223894	72125		100.000

HPLC purity data of compound 5

Analysis Report

<Sample Information>

Sample Name : 50-18359D
Sample ID :
Data Filename : 2023222_50-18359D_006.lcd
Method Filename : NHRI purity test.lcm
Batch Filename : 20230222_hplc.lcb
Vial # : 3-6
Sample Type : Unknown
Injection Volume : 20 uL
Date Acquired : 2023/2/22 10:31:22
Acquired by : System Administrator
Date Processed : 2023/2/23 11:12:49
Processed by : System Administrator

<Chromatogram>

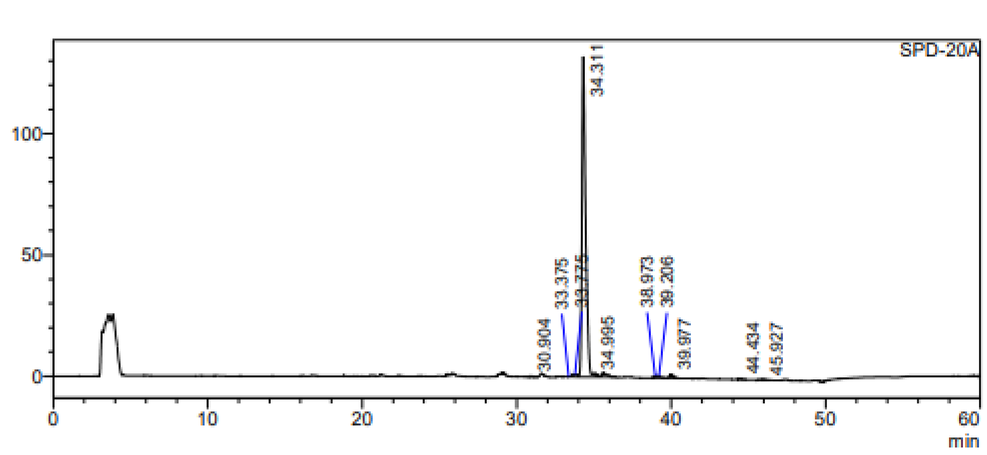

Peak Table

SPD-20A

Peak#	Ret. Time	Area	Height	Conc.	Area%
1	30.904	5169	394	0.221	0.221
2	33.375	1836	171	0.079	0.079
3	33.775	23616	996	1.010	1.010
4	34.311	2238985	132169	95.755	95.755
5	34.995	11218	699	0.480	0.480
6	38.973	11249	908	0.481	0.481
7	39.206	14130	891	0.604	0.604
8	39.977	23386	1409	1.000	1.000
9	44.434	4849	334	0.207	0.207
10	45.927	3794	262	0.162	0.162
Total		2338232	138233		100.000

HPLC purity data of compound 6

Analysis Report

<Sample Information>

Sample Name : 50-18359
Sample ID :
Data Filename : 2023224_50-18359_002.lcd
Method Filename : NHRI purity test.lcm
Batch Filename : 20230224_hplc.lcb
Vial # : 3-2 Sample Type : Unknown
Injection Volume : 20 uL
Date Acquired : 2023/2/24 06:37:52 Acquired by : System Administrator
Date Processed : 2023/3/1 02:11:52 Processed by : System Administrator

<Chromatogram>

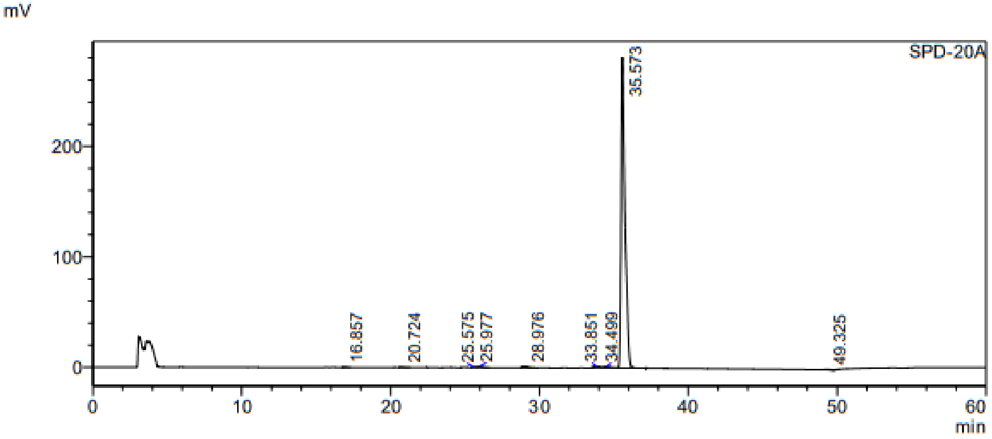

Peak Table

SPD-20A

Peak#	Ret. Time	Area	Height	Conc.	Area%
1	16.857	3884	288	0.076	0.076
2	20.724	6517	373	0.127	0.127
3	25.575	9884	665	0.193	0.193
4	25.977	23021	1154	0.450	0.450
5	28.976	26888	1345	0.525	0.525
6	33.851	39464	1260	0.771	0.771
7	34.499	22958	1108	0.449	0.449
8	35.573	4976317	281310	97.248	97.248
9	49.325	8215	358	0.161	0.161
Total		5117148	287862		100.000

HPLC purity data of compound 7

Analysis Report

<Sample Information>

Sample Name : 50-18390A
Sample ID :
Data Filename : 2023222_50-18390A_009.lcd
Method Filename : NHRI purity test.lcm
Batch Filename : 20230222_hplc.lcb
Vial # : 3-9
Sample Type : Unknown
Injection Volume : 20 uL
Date Acquired : 2023/2/23 01:32:34
Acquired by : System Administrator
Date Processed : 2023/2/23 11:21:21
Processed by : System Administrator

<Chromatogram>

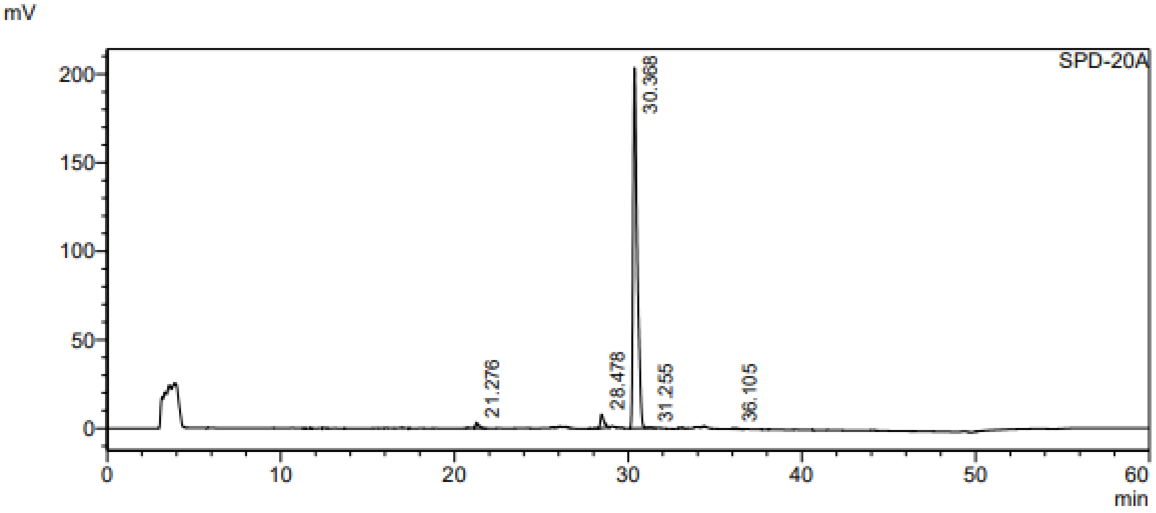

Peak Table

SPD-20A

Peak#	Ret. Time	Area	Height	Conc.	Area%
1	21.276	42484	3084	1.218	1.218
2	28.478	106911	7542	3.064	3.064
3	30.368	3319682	203830	95.148	95.148
4	31.255	10369	592	0.297	0.297
5	36.105	9520	489	0.273	0.273
Total		3488967	215538		100.000

HPLC purity data of compound 8

Analysis Report

<Sample Information>

Sample Name : 50-18390B
Sample ID :
Data Filename : 2023222_50-18390B_010.lcd
Method Filename : NHRI purity test.lcm
Batch Filename : 20230222_hplc.lcb
Vial # : 3-10 Sample Type : Unknown
Injection Volume : 20 uL
Date Acquired : 2023/2/23 02:32:59 Acquired by : System Administrator
Date Processed : 2023/2/23 11:35:04 Processed by : System Administrator

<Chromatogram>

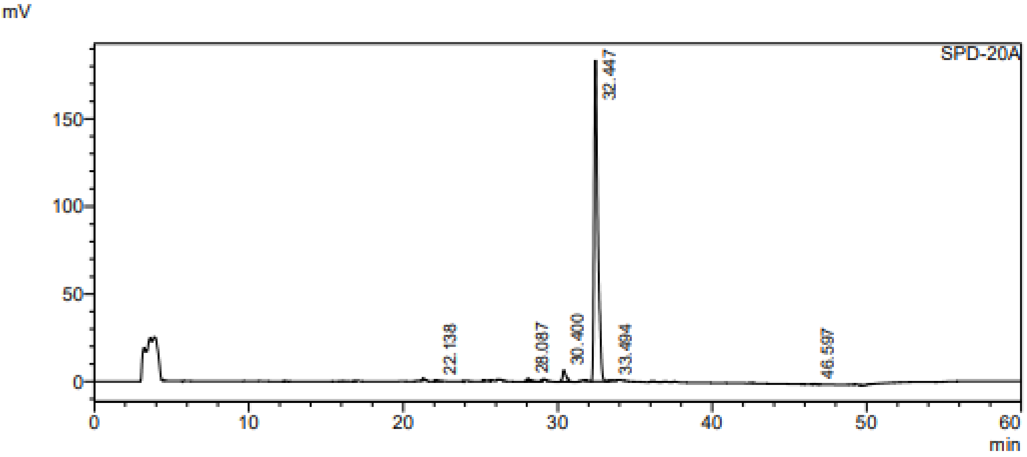

Peak Table

SPD-20A

Peak#	Ret. Time	Area	Height	Conc.	Area%
1	22.138	8913	671	0.279	0.279
2	28.087	28180	1723	0.883	0.883
3	30.400	108517	6675	3.399	3.399
4	32.447	3033882	183761	95.022	95.022
5	33.494	9241	536	0.289	0.289
6	46.597	4076	294	0.128	0.128
Total		3192808	193661		100.000

<u>**HPLC purity data of compound 9**</u>

Analysis Report

<Sample Information>

Sample Name	: 50-18390C		
Sample ID	:		
Data Filename	: 2023222_50-18390C_011.lcd		
Method Filename	: NHRI purity test.lcm		
Batch Filename	: 20230222_hplc.lcb		
Vial #	: 3-11	Sample Type	: Unknown
Injection Volume	: 20 uL		
Date Acquired	: 2023/2/23 03:33:23	Acquired by	: System Administrator
Date Processed	: 2023/2/23 11:26:07	Processed by	: System Administrator

<Chromatogram>

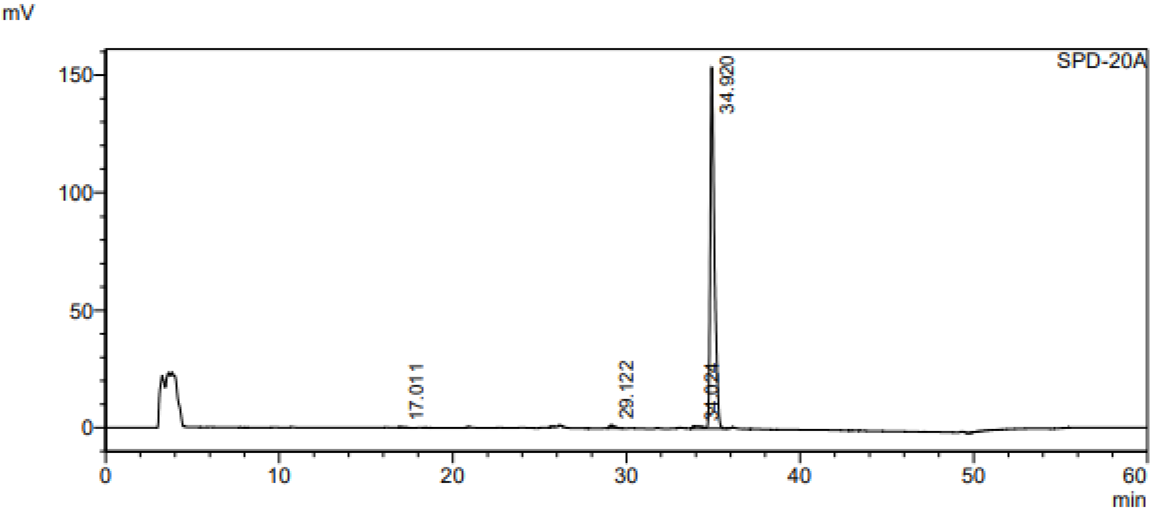

Peak Table

SPD-20A

Peak#	Ret. Time	Area	Height	Conc.	Area%
1	17.011	3373	282	0.127	0.127
2	29.122	25563	1371	0.960	0.960
3	34.024	36999	1039	1.389	1.389
4	34.920	2598225	153585	97.525	97.525
Total		2664160	156276		100.000

HPLC purity data of compound 10

Analysis Report

<Sample Information>

Sample Name	: 50-18930E		
Sample ID	:		
Data Filename	: 2023224_50-18930E_006.lcd		
Method Filename	: NHRI purity test.lcm		
Batch Filename	: 20230224_hplc.lcb		
Vial #	: 3-6	Sample Type	: Unknown
Injection Volume	: 20 uL		
Date Acquired	: 2023/2/24 10:39:22	Acquired by	: System Administrator
Date Processed	: 2023/2/24 11:39:25	Processed by	: System Administrator

<Chromatogram>

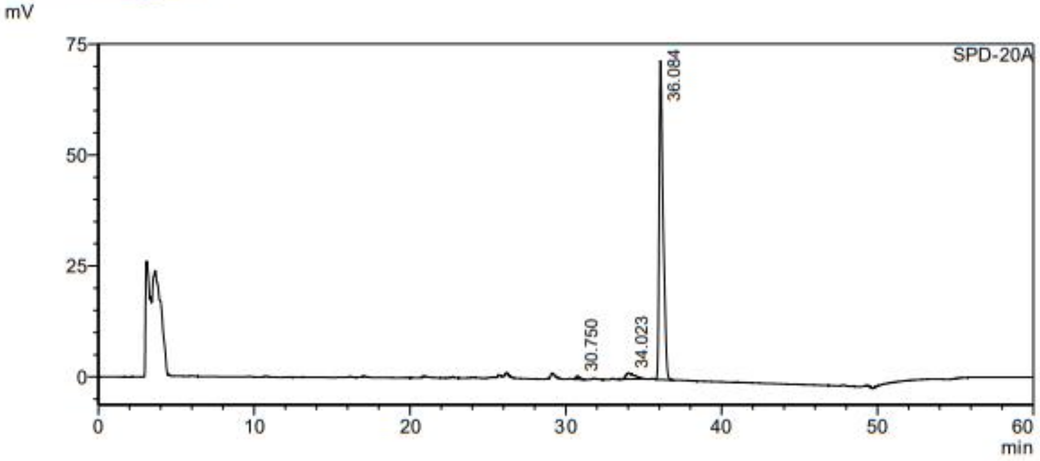

Peak Table

SPD-20A

Peak#	Ret. Time	Area	Height	Conc.	Area%
1	30.750	11021	724	0.830	0.830
2	34.023	51817	1372	3.903	3.903
3	36.084	1264825	71924	95.267	95.267
Total		1327663	74020		100.000

HRMS of compound 1

Elemental Composition Report **Page 1**

Single Mass Analysis
Tolerance = 10.0 mDa / DBE: min = -1.5, max = 50.0
Element prediction: Off
Number of isotope peaks used for i-FIT = 3

Monoisotopic Mass, Even Electron Ions
725 formula(e) evaluated with 11 results within limits (up to 20 closest results for each mass)
Elements Used:
C: 1-100 H: 1-100 N: 1-10 O: 1-10
50-18959a
230313TMU16 256 (2.509) Cm (256:258-(246:252+266:270))

1: TOF MS ES+
8.80e+005

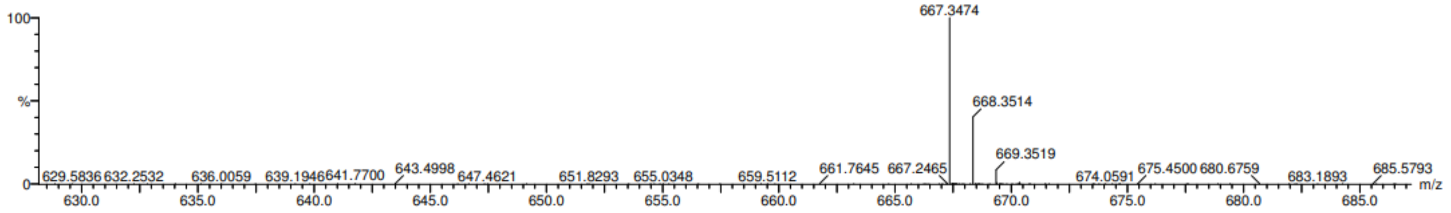

Minimum:				-1.5	
Maximum:		10.0	10.0	50.0	

Mass	Calc. Mass	mDa	PPM	DBE	Formula
667.3474	667.3496	-2.2	-3.3	18.5	C39 H47 N4 O6
	667.3527	-5.3	-7.9	10.5	C28 H47 N10 O9
	667.3455	1.9	2.8	14.5	C34 H47 N6 O8
	667.3568	-9.4	-14.1	14.5	C33 H47 N8 O7
	667.3536	-6.2	-9.3	22.5	C44 H47 N2 O4
	667.3415	5.9	8.8	10.5	C29 H47 N8 O10
	667.3383	9.1	13.6	18.5	C40 H47 N2 O7
	667.3469	0.5	0.7	19.5	C35 H43 N10 O4
	667.3397	7.7	11.5	23.5	C41 H43 N6 O3
	667.3509	-3.5	-5.2	23.5	C40 H43 N8 O2
	667.3437	3.7	5.5	27.5	C46 H43 N4 O

HRMS of compound 2

Elemental Composition Report **Page 1**

Single Mass Analysis
Tolerance = 10.0 mDa / DBE: min = -1.5, max = 50.0
Element prediction: Off
Number of isotope peaks used for i-FIT = 3

Monoisotopic Mass, Even Electron Ions
725 formula(e) evaluated with 10 results within limits (up to 20 closest results for each mass)
Elements Used:
C: 1-100 H: 1-100 N: 1-10 O: 1-10
50-18930d
230313TMU14 262 (2.560) Cm (262:264-(254:257+270:271))

1: TOF MS ES+
4.62e+005

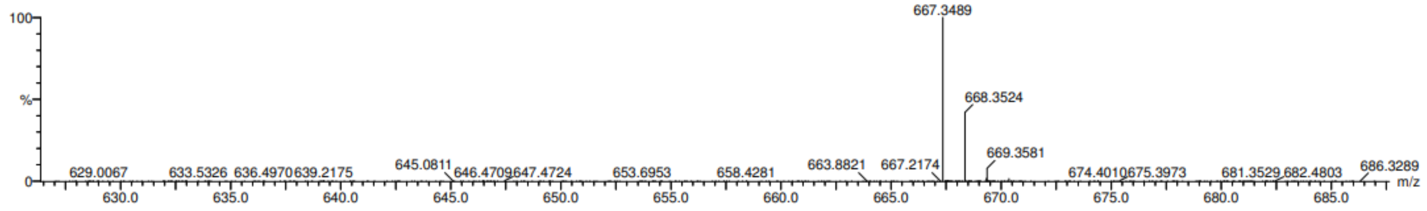

Minimum:				-1.5	
Maximum:		10.0	10.0	50.0	

Mass	Calc. Mass	mDa	PPM	DBE	Formula
667.3489	667.3496	-0.7	-1.0	18.5	C39 H47 N4 O6
	667.3509	-2.0	-3.0	23.5	C40 H43 N8 O2
	667.3469	2.0	3.0	19.5	C35 H43 N10 O4
	667.3455	3.4	5.1	14.5	C34 H47 N6 O8
	667.3527	-3.8	-5.7	10.5	C28 H47 N10 O9
	667.3536	-4.7	-7.0	22.5	C44 H47 N2 O4
	667.3437	5.2	7.8	27.5	C46 H43 N4 O
	667.3415	7.4	11.1	10.5	C29 H47 N8 O10
	667.3568	-7.9	-11.8	14.5	C33 H47 N8 O7
	667.3397	9.2	13.8	23.5	C41 H43 N6 O3

HRMS of compound 3

Elemental Composition Report Page 1

Single Mass Analysis
Tolerance = 5.0 mDa / DBE: min = -1.5, max = 50.0
Element prediction: Off
Number of isotope peaks used for i-FIT = 3

Monoisotopic Mass, Even Electron Ions
742 formula(e) evaluated with 5 results within limits (up to 20 closest results for each mass)
Elements Used:
C: 1-100 H: 1-100 N: 1-10 O: 1-10
50-18959b
230313TMU17 229 (2.238) Cm (228:231-(219:225+235:241)) 1: TOF MS ES+ 2.61e+006

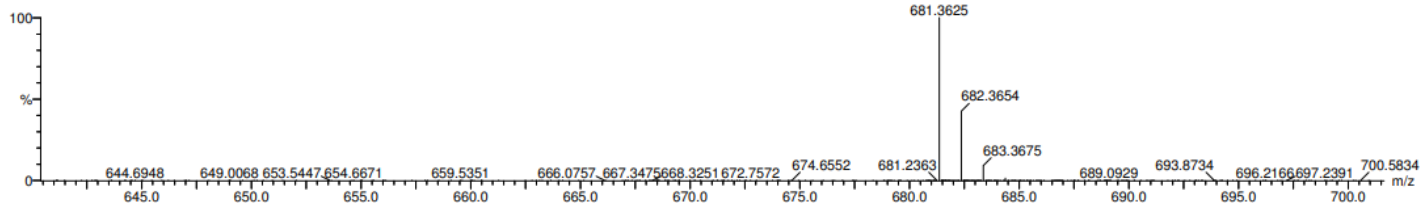

Minimum:				-1.5	
Maximum:		5.0	10.0	50.0	
Mass	Calc. Mass	mDa	PPM	DBE	Formula
681.3625	681.3652	-2.7	-4.0	18.5	C40 H49 N4 O6
	681.3593	3.2	4.7	27.5	C47 H45 N4 O
	681.3612	1.3	1.9	14.5	C35 H49 N6 O8
	681.3665	-4.0	-5.9	23.5	C41 H45 N8 O2
	681.3625	0.0	0.0	19.5	C36 H45 N10 O4

HRMS of compound 4

Elemental Composition Report Page 1

Single Mass Analysis
Tolerance = 10.0 mDa / DBE: min = -1.5, max = 50.0
Element prediction: Off
Number of isotope peaks used for i-FIT = 3

Monoisotopic Mass, Even Electron Ions
758 formula(e) evaluated with 11 results within limits (up to 20 closest results for each mass)
Elements Used:
C: 1-100 H: 1-100 N: 1-10 O: 1-10
50-18959c
230313TMU18 242 (2.369) Cm (240:242-(231:234+250:253)) 1: TOF MS ES+ 1.96e+006

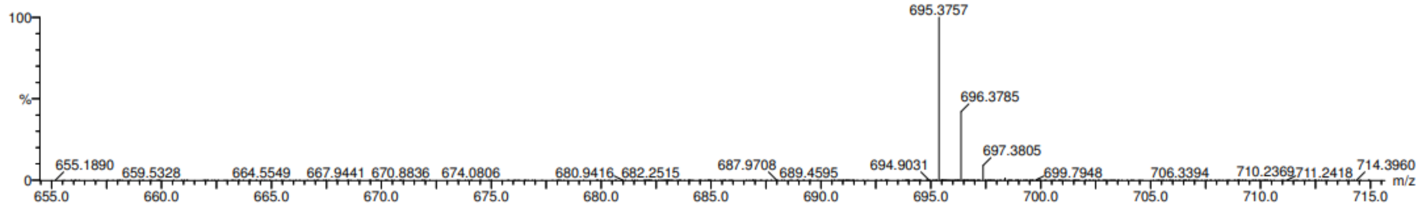

Minimum:				-1.5	
Maximum:		10.0	10.0	50.0	
Mass	Calc. Mass	mDa	PPM	DBE	Formula
695.3757	695.3849	-9.2	-13.2	22.5	C46 H51 N2 O4
	695.3696	6.1	8.8	18.5	C42 H51 N2 O7
	695.3809	-5.2	-7.5	18.5	C41 H51 N4 O6
	695.3750	0.7	1.0	27.5	C48 H47 N4 O
	695.3768	-1.1	-1.6	14.5	C36 H51 N6 O8
	695.3710	4.7	6.8	23.5	C43 H47 N6 O3
	695.3669	8.8	12.7	19.5	C38 H47 N8 O5
	695.3822	-6.5	-9.3	23.5	C42 H47 N8 O2
	695.3728	2.9	4.2	10.5	C31 H51 N8 O10
	695.3840	-8.3	-11.9	10.5	C30 H51 N10 O9
	695.3782	-2.5	-3.6	19.5	C37 H47 N10 O4

HRMS of compound 5

Elemental Composition Report — **Page 1**

Single Mass Analysis
Tolerance = 5.0 mDa / DBE: min = -1.5, max = 50.0
Element prediction: Off
Number of isotope peaks used for i-FIT = 3

Monoisotopic Mass, Even Electron Ions
774 formula(e) evaluated with 5 results within limits (up to 20 closest results for each mass)
Elements Used:
C: 1-100 H: 1-100 N: 1-10 O: 1-10
50-18959d
230313TMU19 254 (2.492) Cm (253:255-(238:246+265:271))

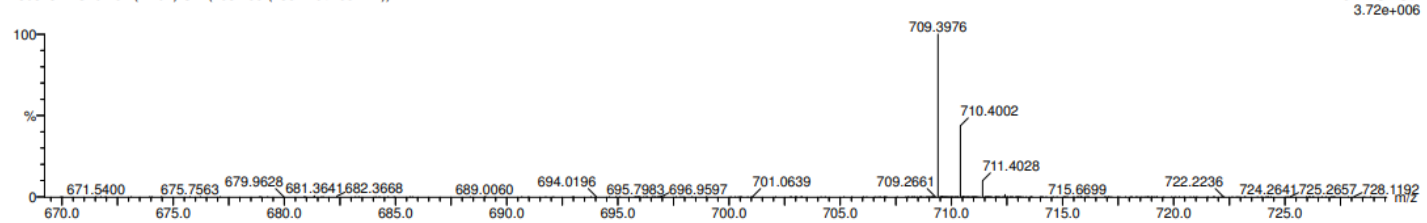

Minimum:				-1.5	
Maximum:		5.0	10.0	50.0	
Mass	Calc. Mass	mDa	PPM	DBE	Formula
709.3976	709.3965	1.1	1.6	18.5	C42 H53 N4 O6
	709.4005	-2.9	-4.1	22.5	C47 H53 N2 O4
	709.3997	-2.1	-3.0	10.5	C31 H53 N10 O9
	709.3978	-0.2	-0.3	23.5	C43 H49 N8 O2
	709.3938	3.8	5.4	19.5	C38 H49 N10 O4

HRMS of compound 6

Elemental Composition Report — **Page 1**

Single Mass Analysis
Tolerance = 10.0 mDa / DBE: min = -1.5, max = 50.0
Element prediction: Off
Number of isotope peaks used for i-FIT = 3

Monoisotopic Mass, Even Electron Ions
812 formula(e) evaluated with 10 results within limits (up to 20 closest results for each mass)
Elements Used:
C: 1-100 H: 1-100 N: 1-10 O: 1-10
50-18959e
230313TMU20 286 (2.786) Cm (285:287-(275:280+296:302))

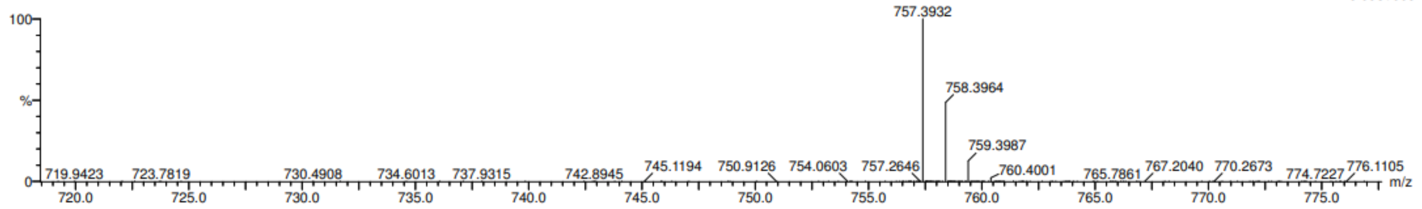

Minimum:				-1.5	
Maximum:		10.0	10.0	50.0	
Mass	Calc. Mass	mDa	PPM	DBE	Formula
757.3932	757.3965	-3.3	-4.4	22.5	C46 H53 N4 O6
	757.3997	-6.5	-8.6	14.5	C35 H53 N10 O9
	757.3925	0.7	0.9	18.5	C41 H53 N6 O8
	757.3853	7.9	10.4	22.5	C47 H53 N2 O7
	757.4005	-7.3	-9.6	26.5	C51 H53 N2 O4
	757.3885	4.7	6.2	14.5	C36 H53 N8 O10
	757.3938	-0.6	-0.8	23.5	C42 H49 N10 O4
	757.3866	6.6	8.7	27.5	C48 H49 N6 O3
	757.3978	-4.6	-6.1	27.5	C47 H49 N8 O2
	757.3906	2.6	3.4	31.5	C53 H49 N4 O

HRMS of compound 7

Elemental Composition Report **Page 1**

Single Mass Analysis
Tolerance = 5.0 mDa / DBE: min = -1.5, max = 50.0
Element prediction: Off
Number of isotope peaks used for i-FIT = 3

Monoisotopic Mass, Even Electron Ions
742 formula(e) evaluated with 5 results within limits (up to 20 closest results for each mass)
Elements Used:
C: 1-100 H: 1-100 N: 1-10 O: 1-10
50-18930a
230313TMU11 264 (2.577) Cm (263:265-(254:258+271:275))

1: TOF MS ES+
3.61e+006

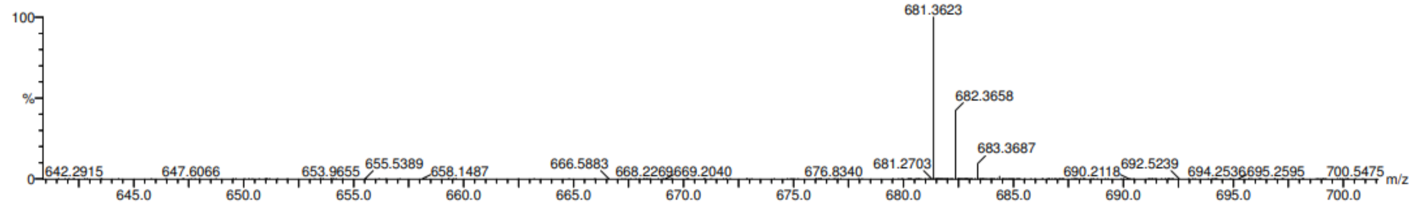

Mass	Calc. Mass	mDa	PPM	DBE	Formula
Minimum:				-1.5	
Maximum:		5.0	10.0	50.0	
681.3623	681.3652	-2.9	-4.3	18.5	C40 H49 N4 O6
	681.3593	3.0	4.4	27.5	C47 H45 N4 O
	681.3612	1.1	1.6	14.5	C35 H49 N6 O8
	681.3665	-4.2	-6.2	23.5	C41 H45 N8 O2
	681.3625	-0.2	-0.3	19.5	C36 H45 N10 O4

HRMS of compound 8

Elemental Composition Report **Page 1**

Single Mass Analysis
Tolerance = 5.0 mDa / DBE: min = -1.5, max = 50.0
Element prediction: Off
Number of isotope peaks used for i-FIT = 3

Monoisotopic Mass, Even Electron Ions
758 formula(e) evaluated with 6 results within limits (up to 20 closest results for each mass)
Elements Used:
C: 1-100 H: 1-100 N: 1-10 O: 1-10
50-18930b
230313TMU12-2 249 (2.429) Cm (248:250-(239:243+255:260))

1: TOF MS ES+
6.41e+006

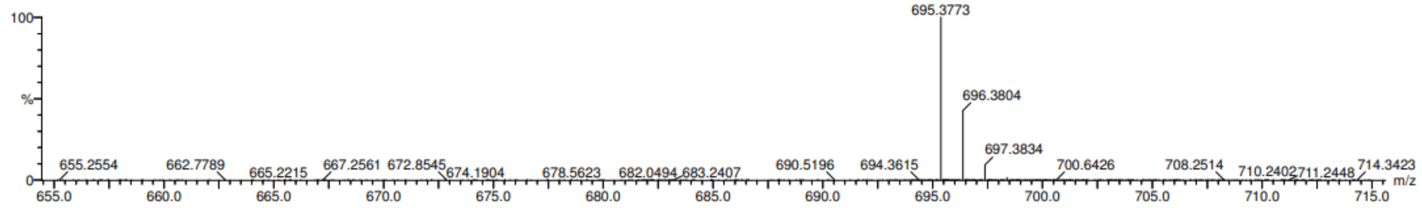

Mass	Calc. Mass	mDa	PPM	DBE	Formula
Minimum:				-1.5	
Maximum:		5.0	10.0	50.0	
695.3773	695.3809	-3.6	-5.2	18.5	C41 H51 N4 O6
	695.3750	2.3	3.3	27.5	C48 H47 N4 O
	695.3768	0.5	0.7	14.5	C36 H51 N6 O8
	695.3728	4.5	6.5	10.5	C31 H51 N8 O10
	695.3822	-4.9	-7.0	23.5	C42 H47 N8 O2
	695.3782	-0.9	-1.3	19.5	C37 H47 N10 O4

HRMS of compound 9

Elemental Composition Report **Page 1**

Single Mass Analysis
Tolerance = 10.0 mDa / DBE: min = -1.5, max = 50.0
Element prediction: Off
Number of isotope peaks used for i-FIT = 3

Monoisotopic Mass, Even Electron Ions
774 formula(e) evaluated with 10 results within limits (up to 20 closest results for each mass)
Elements Used:
C: 1-100 H: 1-100 N: 1-10 O: 1-10
50-18930c
230313TMU13 286 (2.786) Cm (285:287-(272:279+292:299)) 1: TOF MS ES+ 4.42e+006

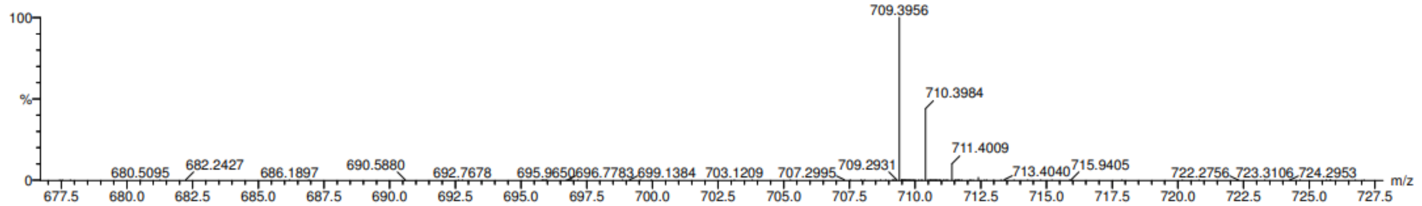

Minimum:				-1.5	
Maximum:		10.0	10.0	50.0	
Mass	Calc. Mass	mDa	PPM	DBE	Formula
709.3956	709.3965	-0.9	-1.3	18.5	C42 H53 N4 O6
	709.3938	1.8	2.5	19.5	C38 H49 N10 O4
	709.3978	-2.2	-3.1	23.5	C43 H49 N8 O2
	709.3925	3.1	4.4	14.5	C37 H53 N6 O8
	709.3997	-4.1	-5.8	10.5	C31 H53 N10 O9
	709.4005	-4.9	-6.9	22.5	C47 H53 N2 O4
	709.3906	5.0	7.0	27.5	C49 H49 N4 O
	709.3885	7.1	10.0	10.5	C32 H53 N8 O10
	709.4037	-8.1	-11.4	14.5	C36 H53 N8 O7
	709.3866	9.0	12.7	23.5	C44 H49 N6 O3

HRMS of compound 10

Elemental Composition Report **Page 1**

Single Mass Analysis
Tolerance = 5.0 mDa / DBE: min = -1.5, max = 50.0
Element prediction: Off
Number of isotope peaks used for i-FIT = 3

Monoisotopic Mass, Even Electron Ions
812 formula(e) evaluated with 5 results within limits (up to 20 closest results for each mass)
Elements Used:
C: 1-100 H: 1-100 N: 1-10 O: 1-10
50-18930e
230313TMU15 269 (2.620) Cm (268:270-(259:263+274:279)) 1: TOF MS ES+ 3.16e+006

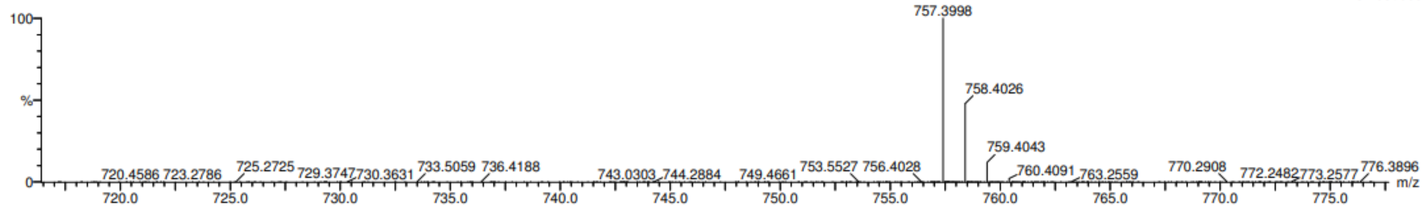

Minimum:				-1.5	
Maximum:		5.0	10.0	50.0	
Mass	Calc. Mass	mDa	PPM	DBE	Formula
757.3998	757.4005	-0.7	-0.9	26.5	C51 H53 N2 O4
	757.3965	3.3	4.4	22.5	C46 H53 N4 O6
	757.4037	-3.9	-5.1	18.5	C40 H53 N8 O7
	757.3978	2.0	2.6	27.5	C47 H49 N8 O2
	757.3997	0.1	0.1	14.5	C35 H53 N10 O9

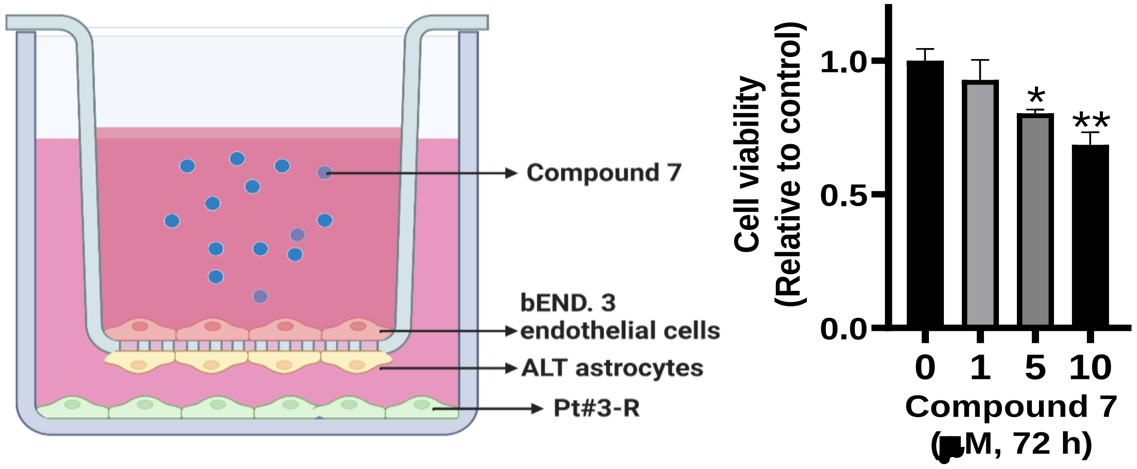

Fig. S1 Compound **7** was added in the upper chamber coated with endothelial cells and astrocytes, and incubated for 72 h. Subsequently, the viability of Pt#3-R in the lower chamber was analyzed by the MTT assay. As shown in Figure B, Compound was able to penetrate BBB to inhibit the proliferation of Pt#3-R cells.

Table S1: Pharmacokinetic data of compound 7

Compound	Rat IV (dose: 2 mg/kg)				Rat PO (dose: 20 mg/kg)				
	$T_{1/2}$ (hr)	CL (ml/min/kg)	V_{ss} (l/kg)	$AUC_{(0-inf)}$ (ng/mL*hr)	$T_{1/2}$ (hr)	C_{max} (ng/ml)	T_{max} (hr)	$AUC_{(0-inf)}$ (ng/mL*hr)	F (%)
**MPT1A059**	0.8 ±0.0	81.8 ± 5.8	3.0 ± 0.5	470 ±32	1.8 ± 0.5	23.8 ± 8.9	1 ± 0	106 ± 62	2.3%

Drug Conc. (ng/mL) Sample	Time (hr)	Avg.	S.D.
IV	0.03	2793	527
	0.08	929	100
	0.25	406	7.6
	0.5	218	11.0
	1	76.6	4.8
	2	30.5	3.5
	4	5.0	1.9
	6	1.1	0.3
	8	ND	NA
	24	ND	ND

Drug Conc. (ng/mL) Sample	Time (hr)	Avg.	S.D.
PO	0.25	23.0	31.6
	0.5	11.1	2.9
	1	23.8	8.9
	2	15.3	8.9
	4	18.1	19.1
	6	4.0	3.3
	8	2.9	2.4
	24	ND	NA
